# Synthesis of l-β-(6-azulenyl)alanine and the fluorescent actin disruptor (6-azuleno)chalasin H†

**DOI:** 10.1039/d5ra04702a

**Published:** 2025-07-22

**Authors:** Maurice Hauser, Katharina Schmidt, Leonard Beiderwieden, Cheng Yi, Kjeld Gerdes, Markus Kalesse, Jennifer Gerke, Theresia E. Stradal, Russell J. Cox

**Affiliations:** a Institute for Organic Chemistry and BMWZ, Leibniz University of Hannover Schneiderberg 38 30167 Hannover Germany russell.cox@oci.uni-hannover.de; b Department of Cell Biology, Helmholtz Centre for Infection Research Inhoffenstraße 7 38124 Braunschweig Germany

## Abstract

l-β-(6-Azulenyl)alanine was synthesised for the first time. Supplementation of this compound to *Pyricularia grisea* Δ*pyiA* led to the biosynthesis of the unnatural (6-azuleno)chalasin H and its 1′-bromo congener that have unprecedented natural product skeletons and that are both fluorescent and highly cytotoxic, with IC_50_ of 0.18 μg mL^−1^*vs.* L929 cells *in vitro*. Actin staining showed that both compounds are potent, but partially reversible, actin disruptors.

## Abstract 2

l-β-(6-Azulenyl)alanine was synthesised for the first time.

## Graphical abstract

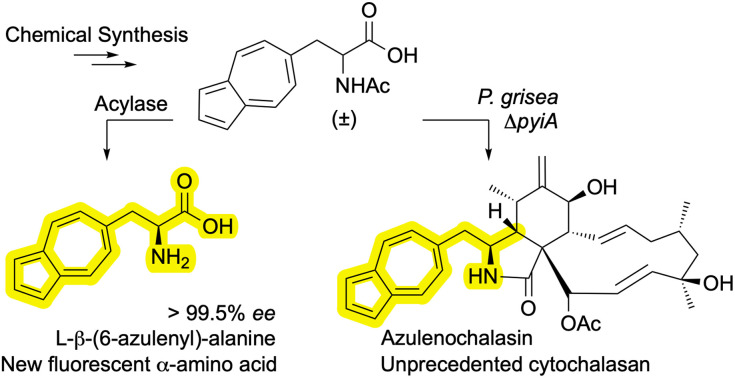

Cytochalasans, such as pyrichalasin H 1a, are a large family of fungal specialized metabolites that are known to bind strongly to actin.^1–3^ This activity prevents elongation of actin filaments and gives cytochalasans corresponding potent bioactivities. Pyrichalasin H 1a is biosynthesised by a hybrid iterative polyketide synthase non-ribosomal peptide synthetase (iPKS-NRPS, Scheme 1) known as PyiS.^4^ This multi-functional enzyme produces a specific β-keto polyketide that is attached to an acyl carrier protein (ACP). The adenylation (A) domain of the PyiS NRPS activates and selects *O*-methyl tyrosine 2b and covalently links it to the downstream thiolation (T) domain. The polyketide is then transferred to the amine of the amino acid by the condensation (C) domain to create an enzyme-bound acylaminothiolester 3. The thiolester is reduced to release an aldehyde as the first enzyme-free intermediate,^5^ and Knoevenagel^6^ and Diels Alder^7^ enzymes then catalyse two cyclisations to afford the distinctive hexahydroisoindolone core of the unfunctionalised pyrichalasin V 4. Finally, tailoring enzymes (PyiBDGH) create the mature pyrichalasin H 1a.

**Scheme 1:**
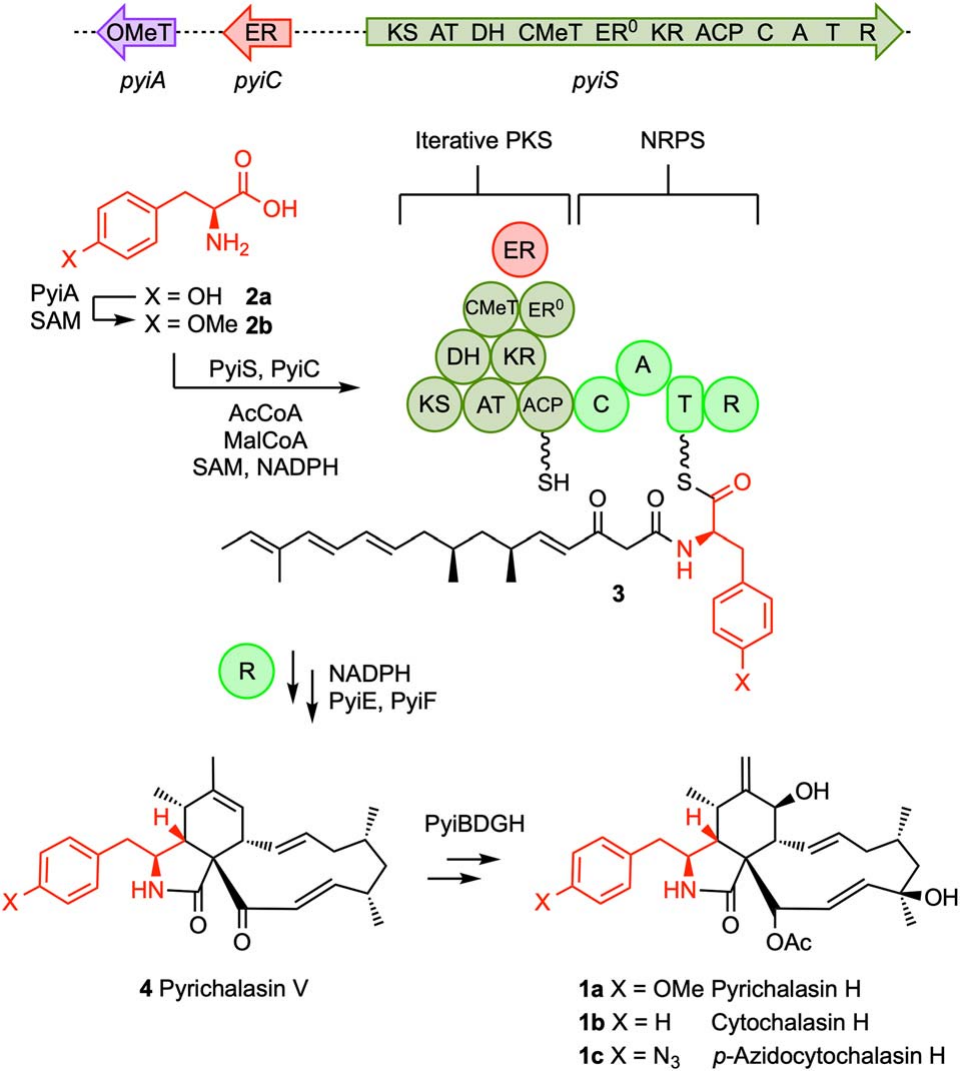
Biosynthesis and mutasynthesis of cytochalasans.

Other cytochalasans also incorporate aromatic amino acids. For example, cytochalasin H 1b is formed from phenylalanine, while the related chaetoglobosins (*vide infra*) are built from tryptophan and a longer polyketide. In previous work we prevented the biosynthesis of *O*-methyl tyrosine in the pyrichalasin H 1a producing fungus *Pyricularia grisea* by knocking out the *pyiA* gene encoding an *O*-methyltransferase (*O*-MeT) from the *pyi* biosynthetic gene cluster (BGC).^8^

In this mutant, endogenous phenylalanine can weakly complement the missing *O*-methyl tyrosine 2b to produce low titres of cytochalasin H 1b. But, when supplemented by various *p*-substituted phenylalanines, good titres of the corresponding *p*-substituted cytochalasins can be produced in a process known as mutasynthesis.^9^ For example, *p*-azidophenylalanine leads to good production of *p*-azidocytochalasin H 1c. These observations indicate that the A-domain of the pyrichalasin NRPS can accept a wide range of *p*-substituted phenylalanines and that the reductive release (R), cyclisation (PyiE, PyiF) and late-acting tailoring enzymes (PyiBDGH) are unaffected by changes to the amino acid component.

Recent interest in the area of aromatic amino acids has shown that they can often be replaced by corresponding azulenylalanines. Azulene has proven itself to be a useful molecular probe because of its inherent fluorescence.^10,11^ For example, l-β-(1-azulenyl)alanine 5 has been used to replace tryptophan in peptides without significant structural perturbations. Its fluorescence can be quenched by unprotonated histidine, providing a sensitive tool for the measurement of histidine p*K*_a_ values in proteins.^12^ Kalesse and coworkers have built azulene into the structure of the natural product argyrin.^13^

l-β-(1-Azulenyl)alanine 5 is a carbocyclic analog of l-tryptophan. From our experiments in the area of pyrichalasin H biosynthesis we already know that tryptophan cannot replace *O*-methyl tyrosine 2b during the biosynthesis of pyrichalasin H 1a.^7^ However, we reasoned that the isomeric β-(6-azulenyl)alanine 6a might mimic *O*-methyl tyrosine 2b and be incorporated in its place *via* mutasynthesis (Scheme 1A), providing an inherently fluorescent cytochalasin. But, while β-(1-azulenyl)alanine 5 is known,^14^ β-(6-azulenyl)alanine 6a has not been reported. We therefore set out to synthesise this unusual amino acid and determine if it can be used to produce new members of the cytochalasan family.

The synthesis starts with reaction of *N*-butyl-4-methyl pyridinium bromide 7 with cyclopentadiene under basic conditions with microwave irradiation to give the known 6-methylazulene 8 (Scheme 2B). Treatment of 8 with *N*,*N*-dimethylformamide dimethylacetal (DMF-DMA) in refluxing DMF then afforded the known enamine 9. Oxidative cleavage to the aldehyde 10a (ref. 15) was followed by borohydride reduction to the corresponding primary alcohol 11a. Treatment of 11a with tosylchloride in the presence of Et_3_N afforded the chloride 12a rather than the expected tosylate, but 12a reacted in the next step without problem. Thus, 12a was treated with the sodium salt of diethyl acetamidomalonate^16^ in DMF to afford the fully protected amino acid 13a. Ester hydrolysis and decarboxylation then gave the racemic *N*-acetyl amino acid 14a. Finally, treatment of 14a with porcine kidney acylase in aqueous buffer at 37 °C for 3 days gave the free amino acid 6a. Marfey's analysis showed this to be the expected l-enantiomer formed in >99.5% enantiomeric excess (see ESI, Fig. S24†).

**Scheme 2:**
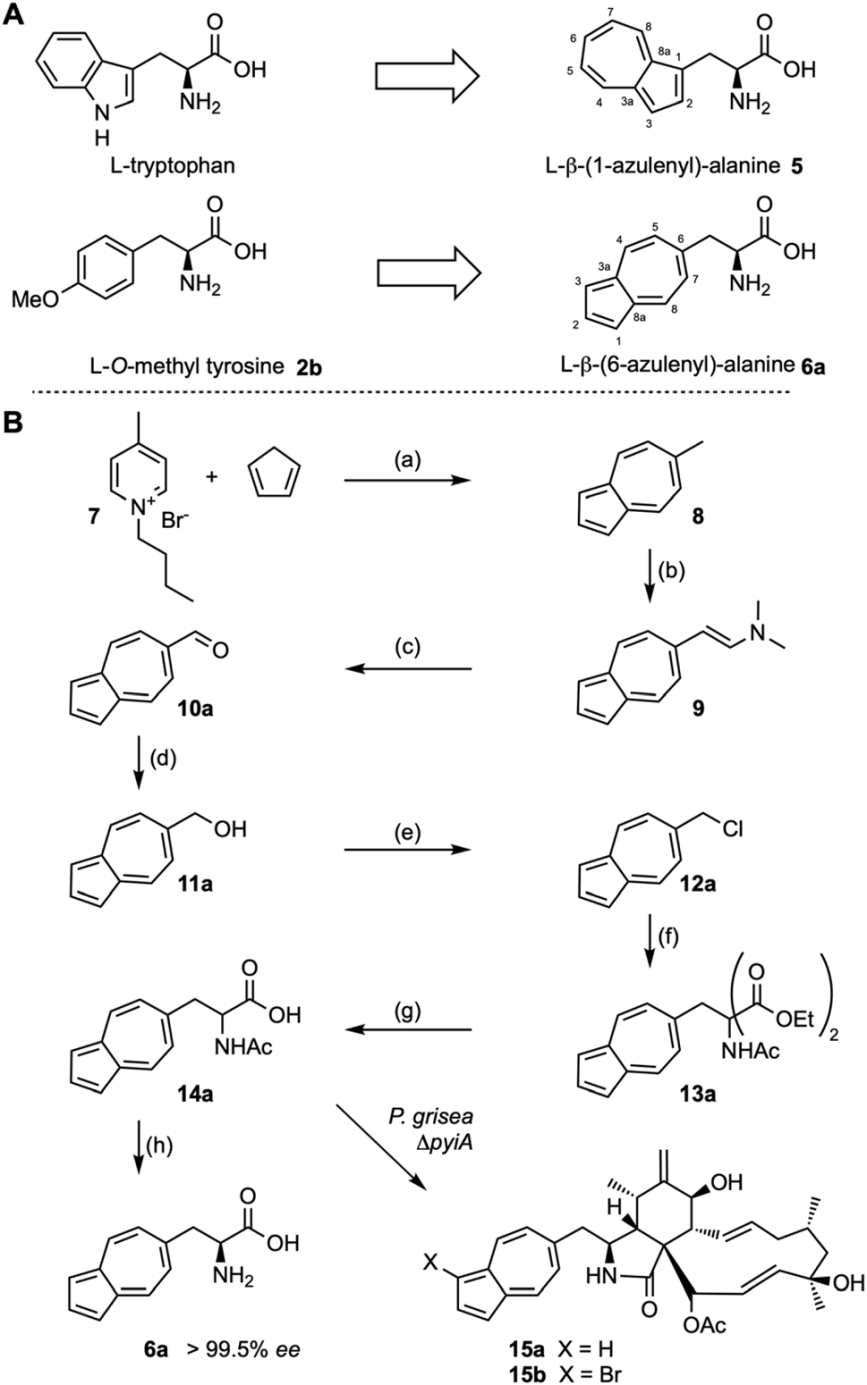
(A) Comparison of structures of tryptophan and *O*-methyl tyrosine with l-β-(1- and l-β-(6-azulenyl)alanine respectively; (B) synthesis of l-β-(6-azulenyl)alanine and azulenochalasins. Reagents and conditions: (a) NaH, DMF, microwave, 200 °C, 15 min, 76%; (b) DMF-DMA, DMF, 140 °C, 7 h; (c) NaIO_4_, THF/H_2_O, RT, 1 h, 69% over two steps; (d) NaBH_4_, CH_2_Cl_2_/MeOH, 0 °C, 30 min, 93%; (e) TsCl, Et_3_N, CH_2_Cl_2_, RT, 16 h; (f) diethyl acetamidomalonate, NaH, DMF, RT, 4 h, 67% over two steps; (g) LiOH·H_2_O, THF/H_2_O, 50 °C, 2 d, 93%; (h) acylase, phosphate buffer, 37 °C, 3 d, 41%.

We cultivated *P. grisea* Δ*pyiA* in liquid media for 7 days. Pulsed supplementation of the culture with 6a over the first 4 days was followed by a further 3 days of growth. Extraction of the fermentation broth with EtOAc gave an extract that was analysed by LCMS. This showed the presence of a new compound corresponding to 15a (calc. for [C_34_H_41_NO_5_]H^+^ 544.3063, obs. 544.3050) with the distinctive UV absorption spectrum of azulene at 278 and 284 nm, albeit in low titre. Repeat of this experiment using the azulenylalanine *N*-acetate 14a led to better titres of the new compound (Scheme 1B and see ESI Fig. S25–S29† for details). However, feeding with the fully protected diester 13a was unsuccessful. Purification of the new compound was achieved using mass-directed HPLC-fractionation from the feeding experiment with 14a to give 1.3 mg L^−1^ of 15a as a blue solid.

Full characterisation of 15a by NMR confirmed the expected structure as (6-azuleno)chalasin H. In particular, the ^1^H NMR displayed almost identical resonances for the macrolide and hexahydroisoindolone core as pyrichalasin H 1a, but the distinctive seven aromatic protons of azulene between 7.0 and 8.5 ppm replaced the *p*-methoxyphenyl signals of 1a (Fig. S39†).

The compound showed a typical maximum UV absorption of 340 nm and fluorescence emission at 380 nm. In an attempt to increase the titre of 15a, the fermentation was repeated using baffled flasks that have the effect of improving oxygenation and general fungal growth characteristics. Surprisingly, under these conditions, a new compound 15b was observed (see ESI Fig. S30–S32† for details). The mass of the new compound corresponds to a single bromination of 15a, (calc. for [C_34_H_40_^79^BrNO_5_–H]^−^ 620.2012, obs. 620.1989) and this was confirmed by ^1^H NMR and MS analysis of the purified compound.

Bromination was determined at the 1′-position of azulene by NMR, and consistent with an observed shift in the UV spectrum from 278 and 284 nm to 284 and 295 nm; and fluorescence absorption and emission shifts to 350 nm and 390 nm, respectively (see ESI† for details).^17^

Encouraged by these results, we attempted to broaden the synthetic scope. First we synthesised *N*-acetyl β-(6-(1-methyl)azulenyl)alanine 14b using a parallel synthetic route starting from 1-methylcyclopentadiene (Scheme 3, see ESI† for details). This was supplemented to *P. grisea* Δ*pyiA*, but no incorporation into a new cytochalasan skeleton was observed. In a second experiment we supplemented β-(1-azulenyl)alanine 5 (ref. 13) to a growing culture of *Chaetomium globosum* that produces the tryptophan-derived chaetoglobosin A 16 (Scheme 3).^18^ In this case the supplemented β-(1-azulenyl)alanine 5 must compete with endogenous tryptophan for incorporation by the chaetoglobosin PKS-NRPS. LCMS and HRMS analysis of extracts from this experiment showed the clear production of a new compound corresponding to the expected (1-azuleno)globosin A 17 (*e.g.* calc for [C_34_H_37_NO_5_]Na^+^ 562.2569, obs. 562.2594, *λ*_max_ 278 nm, see ESI Fig. S33–S36† for details). But, purification led to extensive decomposition, and inability to fully characterise this material.

**Scheme 3:**
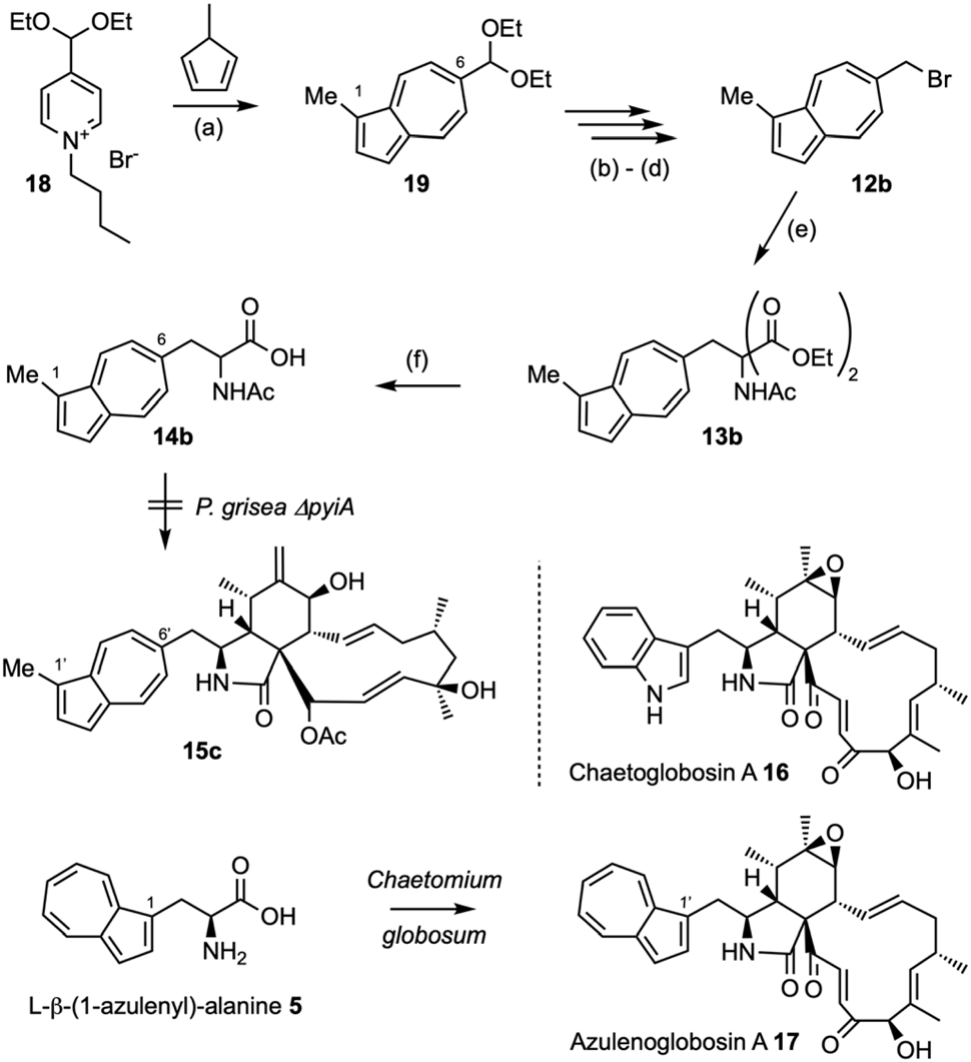
Synthesis of *N*-acetyl-β-(6-(1-methylazulenyl)alanine) 14b and incorporation of l-β-(1-azulenyl)alanine 5 into the chaetoglobosin A skeleton. Reagents and conditions: (a) DMF, NaH, 44%; (b) aq. HCl, 81%; (c) NaBH_4_, MeOH/CH_2_Cl_2_, 92%; (d) CBr_4_, PPh_3_, CH_2_Cl_2_; (e) NaH, DMF, diethyl acetamidomalonate, 42% over two steps; (f) LiOH·H_2_O, H_2_O/THF, 95%.

Simulated docking of 6-azulenochalasin H 15a*vs.* an actin monomer structure (PDB: 3eku, Fig. 1A) using DiffDock-L^19^ showed that azulenochalasin can bind in the barbed end pocket. Purified 15a and 15b were tested for cytotoxicity in mouse fibroblast L929 cells. Titration studies showed IC_50_ values of 0.18 μg mL^−1^ in L929 for both 15a and 15b.

**Fig. 1:**
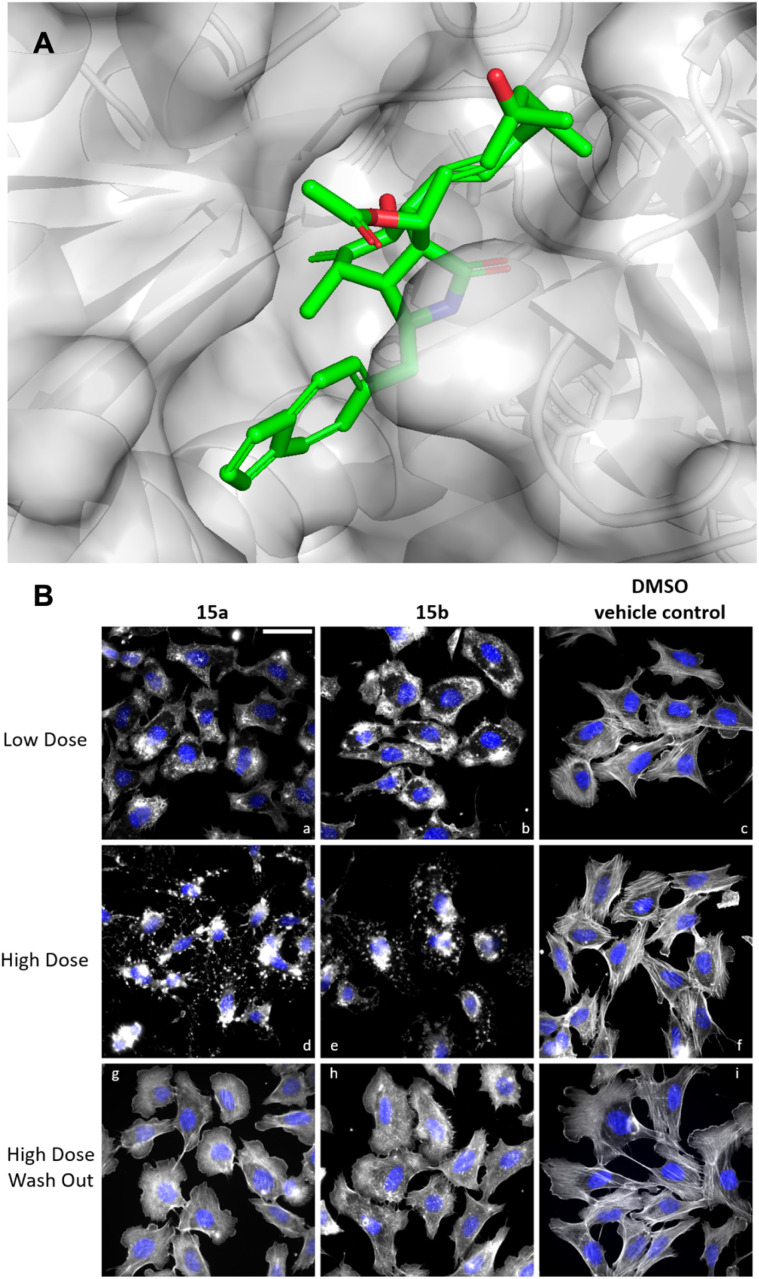
Biological activity of azulenochalasins. (A) Simulated docking of azulenochalasin 15a onto an actin monomer (PDB: 3eku) showing binding at the barbed end. (B) *in cellulo* activity of azulenochalasins 15a and 15b on the actin cytoskeleton of human osteosarcoma (U-2OS) cells upon low dose (0.18 μg mL^−1^) and high dose (0.9 μg mL^−1^) treatment as well as high dose treatment followed by wash out and 1 h cultivation in fresh culture medium. Filamentous actin (F-actin) was stained using Atto488-phalloidin (greyscales) and nuclear DNA using DAPI (pseudo coloured in blue). Scale bar in (a) corresponds to 50 μm.

Compound 15a was also tested in KB3.1 cells showing cytotoxicity of 0.029 μg mL^−1^. Actin disruption activity was tested in human osteosarcoma (U-2OS) cells. Cells were seeded on fibronectin-coated cover slips and treated with low dose (0.18 μg mL^−1^) and high-dose (0.9 μg mL^−1^) concentrations of respective cytochalasin. After 1 h incubation, cells were fixed with 4% pre-warmed paraformaldehyde and permealized using 0.1% Triton X-100. Cellular filamentous actin (F-actin) was stained using Atto488-phalloidin. Azulenochalasin 15a and its brominated congener 15b showed significant activity on cellular actin structures, ranging from larger F-actin accumulations up to complete disruption of the network with increasing compound concentration. Notably, the actin disruption observed was not entirely reversible after 1 h recovery period. The cells still lacked pronounced stress fibres after 15a-treatment, and those previously exposed to 15b continued to exhibit F-actin accumulation, as well as reduced stress fibres and lamellipodia, as compared to the DMSO control.

## Conclusions

Azulenylalanines are inherently fluorescent analogs of aromatic amino acids. Here we have synthesised l-β-(6-azulenyl)alanine 6a for the first time and in high *ee*. Compound 6a proves to be an analog of *O*-methyl tyrosine 2b and is incorporated by the pyrichalasin H synthetase A-domain to create a new cytochalasan skeleton 15a. Although 6a was fed to, and incorporated by, *P. grisea*, the *N*-acetate 14a was a better precursor. Presumably 14a is more easily imported by *P. grisea* than 6a, and then hydrolysed *in vivo* to release 6a as the actual precursor of 15a. (6-Azuleno)chalasin H 15a itself then appears to be brominated at the 1-position by, most likely, an unknown halogenase to create 15b.

Remarkably, the new azulenochalasins 15a and 15b retain the potent actin-disruption properties of native cytochalasans, while possessing inherent fluorescence. The results also indicate that the adenylation (A) domain of the pyrichalasin H PKS-NRPS possesses a remarkable ability to recognise varied aromatic amino acids including azulenes. However, the more-bulky 1-methyl analog, was not incorporated suggesting that the formation of the observed (6-(1-bromo)azuleno)chalasin H 15b most-likely arises by adventitious bromination of 15a itself, rather than by bromination of 6a before its incorporation by the PyiS A-domain. Contrastingly, the chaetoglobosin synthetase is known to accept brominated tryptophan as a substrate during the biosynthesis of chaetoglobosin.^18^ Attempts to extend this work to incorporate the tryptophan analog l-β-(1-azulenyl)alanine 5 into chaetoglobosin A was partially successful, also suggesting that the chaetoglobosin A-domain possesses usefully broad substrate selectivity. Detection of the expected compound 17 by LCMS shows that the chaetoglobosin PKS-NRPS A-domain must recognise and activate 5, but because of competition between 5 and endogenous tryptophan in this experiment, the titre of 17 was very low and insufficient material could be obtained for biological testing or full characterisation. However these results show the remarkable potential of the azulenoalanines as replacements for aromatic amino acids in fungal natural products.

## Author contributions

The project was devised by RJC, JG and MH. RJC, JG, MK and TES supervised the projects. All synthesis and characterisation work was done by MH, KG, CY and LB. KS performed the biological testing and microscopy. RJC performed the simulated docking. The MS was prepared by RJC and polished and approved by all authors.

## Conflicts of interest

RJC is joint editor-in-chief of *RSC Advances*.

## Supplementary Material

RA-015-D5RA04702A-s001

## Data Availability

The data supporting this article have been included as part of the ESI.†
