# Supplementary material for: Synthesis of l-β-(6-azulenyl)alanine and the fluorescent actin disruptor (6-azuleno)chalasin H
Source: RSC Adv. 2025 Jul 22;15(32):26048–51. doi: 10.1039/d5ra04702a (PMC12281303; doi:10.1039/d5ra04702a)
Supplement: RA-015-D5RA04702A-s001 [file RA-015-D5RA04702A-s001.pdf]

Supplementary Information (SI) for RSC Advances.
This journal is © The Royal Society of Chemistry 2025

Synthesis of L-β-(6-Azulenyl)alanine and the Fluorescent Actin Disruptor (6-Azuleno)chalasin H

Electronic Supplementary Information

Maurice Hauser, Katharina Schmidt, Leonard Beiderwieden, Cheng Yi, Kjeld Gerdes, Markus Kalesse, Jennifer Gerke, Theresia E. Stradal and Russell J. Cox*

Contents

1.	General Methods	1
2.	Synthesis and Characterisation	2
3.	Biological Testing	40
4.	References	41

1. General Methods

NMR

Bruker Ultrashield 400 equipped with with Avance-I console and a DuL ^{1}H, ^{13}C probe at 400 MHz (^{1}H)/101 MHz (^{13}C) Bruker Avance 500 (equipped with a cryo-cooled probe) at 500 MHz (^{1}H)/125 MHz (^{13}C)) and 600 MHz (^{1}H)/150 MHz (^{13}C) spectrometers were used for all NMR analysis. Standard parameters were used for the collection of 2D spectra (^{1}H, ^{1}H-correlation spectroscopy [COSY], heteronuclear single-quantum coherence [HSQC] and Heteronuclear Multiple Bond Correlation (HMBC) spectra in the indicated solvents. ^{1}H and ^{13}C spectra are referenced relative to residual protonated solvents. All δ values are quoted in ppm and all *J* values in Hz.

Analytical LCMS and Low Resolution MS

LCMS data were obtained using a Waters LCMS system comprising of a Waters 2767 autosampler, Waters 2545 pump system and a Phenomenex Kinetex column (2.6 µ, C_{18}, 100 Å, 4.6 × 100 mm) equipped with a Phenomenex Security Guard precolumn (Luna C_5 300 Å) eluted at 1 mL/min. Detection was performed by Waters 2998 diode array detector between 200 and 600 nm; Waters 2424 ELSD and Waters SQD-2 mass detector operating simultaneously in ES^+ and ES^- modes between 100 *m/z* and 650 *m/z*. Solvents were A, HPLC-grade H_2O containg 0.05% formic acid; and B, HPLC-grade CH_3CN containing 0.045% formic acid. Gradients were as follows: Method 1 (optimised for non-polar compounds): 0 min, 10% B; 10 min 90% B; 12 min, 90% B; 13 min, 10% B and 15 min, 10% B. Method 2 (optimised for polar compounds): 0 min, 10% B; 10 min 40% B; 12 min, 90% B; 13 min, 10% B and 15 min, 10% B.

High Resolution MS

HRMS was obtained using a UPLC system (Waters Acquity Ultraperformance, running the same method and column as above) connected to a Q-TOF Premier mass spectrometer.

Preparative LCMS

Compounds were purified using a Waters mass-directed autopurification system consisting of a Waters 2545 pump and Waters 2767 autosampler. The chromatography column was a Phenomenex Kinetex Axia column (5µ, C_{18}, 100 Å, 21.2 × 250 mm) fitted with a Luna C_5 300 Å Phenomenex Security Guard precolumn. The column was eluted at 20 mL/min at 22 °C. Solvents used were A, H_2O + 0.05% formic acid; and B, CH_3CN + 0.045% formic acid. All solvents were HPLC grade. The column outlet was split (100:1) and the minority flow was supplemented with HPLC-grade MeOH + 0.045% formic acid to 1000 µL/min and diverted for interrogation by diode array (Waters 2998) and evaporative light-scattering (Waters 2424) detectors. The flow was also analysed by mass spectrometry (Waters SQD-2 in ES^+ and ES^- modes). Desired compounds were collected into glass test tubes. Combined fractions were evaporated *in vacuo*, then dissolved directly in HPLC-grade MeOH to make the final concentration 10 mg / mL, 300 µl solution was loaded into the column for purification.

2. Synthesis and Characterisation

All reagents and anhydrous solvents were purchased from commercial suppliers and used without further purification unless otherwise stated. All reactions were carried out in oven-dried glassware equipped with a magnetic stirrer and under a positive pressure of N_2 using standard Schlenk-line techniques. Reactions requiring cooling to 0 °C were conducted in an ice bath and equipped with a thermometer. Heating of reactions was achieved in a silicone oil bath equipped with a contact thermometer. Removal of organic solvents after each reaction was performed under reduced pressure using a rotary evaporator with a water bath temperature of 40 °C, unless otherwise stated.

Synthetic procedures that required microwave heating were conducted using the Biotage® Initiator+ (EU). Prior to irradiation, the vials were sealed under a N_2 atmosphere. The microwave settings were then based on vial size and solvent. Generally, without pre-stirring, the vial type 20 mL, and the absorption level was set to normal based on the solvent used (DMF).

Thin-layer chromatography (TLC) was performed on pre-coated TLC sheets ALUGRAM® Xtra SIL G/UV 254 from Macherey-Nagel (0.2 mm layer thickness, median pore size 60 Å, and with a fluorescent indicator). Generally, the azulene derivatives didn't need any additional visualisation method. For purification purposes additional staining with $KMnO_4$ or Cerium (IV) diving baths and subsequent heating with a heating gun might be required.

Flash chromatography for purification was performed using silica gel from Macherey-Nagel (silica 60 M, particle diameter 40 – 63 μM), and eluents are given in brackets. Column size and filling level were adapted to the purification.

8 6-methylazulene.[1]

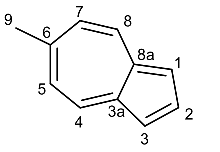

Chemical Formula: $C_{11}H_{10}$
Exact Mass: 142.0783

Cyclopentadiene (0.41 mL, 5.00 mmol) was added to a suspension of NaH (161.30 mg, 4.2 mmol, 60 % in mineral oil) in DMF (10 mL) in a dry 20 mL microwave vial. Cyclopentadiene was prepared freshly by distilling dicyclopentadiene at 200 °C. After stirring for 15 min at room temperature (RT) a solution of 1-butyl-4-methylpyridinium bromide (967 mg, 4.2 mmol) in DMF (7 mL) was added and stirred for 5 min until the solution turned dark red. The vial was sealed, placed in the microwave and heated to 200 °C for 15 min. The resulting dark-green solution was extracted with hexane (6 × 30 mL) to give a blue solution, which was washed with equal volumes of water (2 ×) and brine (1 ×), dried over Na_2SO_4, filtered and the solvent was removed *in vacuo*. The resulting black residue was purified by column chromatography (100 % petroleum ether) to give 6-methylazulene as dark blue crystals (454 mg, 76 %). $\mathbf{R_f}$ (petroleum ether): 0.35; $\mathbf{UV_{\lambda max}}$ (CH_3CN:H_2O): 233, 276, 282, 343 nm; **^{1}H-NMR** (400 MHz, $CDCl_3$): δ = 8.22 (d, *J* = 10.0 Hz, 2H, H-4,8), 7.79 (t, *J* = 3.7 Hz, 1H, H-2), 7.33 (d, *J* = 3.7 Hz, 2H, H-1,3), 7.09 (d, *J* = 10.0 Hz, 1H, H-5,7), 2.65 (s, 3H, H-9); **^{13}C-NMR** (101 MHz, $CDCl_3$): δ = 148.8 (C-3a,8a), 138.7 (C-6), 135.7 (C-4,8), 135.5 (C-2), 124.2 (C-5,7), 117.9 (C-1,3), 28.1 (C-9); **ES-MS (ES+)**: calculated for $[M+H]^+$:143.1, found at $[C_{11}H_{10}H]^+$: 143.1

9 1-(6-azulenyl)-2-dimethylamino-ethene.[1]

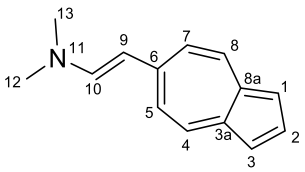

Chemical Formula: $C_{14}H_{15}N$
Exact Mass: 197.1204

To a solution of 6-methylazulene **8** (200 mg, 1.41 mmol) in DMF (1.5 mL) was added *N,N* - Dimethylformamide dimethyl acetal (0.24 mL, 1.83 mmol) and the mixture was stirred at 140 °C for 6 h. To the resulting brown mixture was added EtOAc (30 mL) and the organic phases were washed with water (5 × 20 mL), dried over $MgSO_4$, filtered and evaporated to dryness. The obtained grey solid (277 mg, quant.) was used without further purification. **^{1}H-NMR** (400 MHz, $CDCl_3$): δ = 8.05 (d, *J* = 10.8 Hz, 2H, H-4,8), 7.51 (t, *J* = 3.7 Hz, 1H, H-2), 7.14 (t, *J* = 13.5 Hz, 1H, H-10), 7.14 (d, *J* = 3.6 Hz, 2H, H-1,3), 7.00 (d, *J* = 10.5 Hz, 2H, H-5,7), 5.33 (d, *J* = 13.4 Hz, 1H, H-9), 2.96 (s, 6 H, H-12,13); **^{13}C-NMR** (101 MHz, $CDCl_3$): δ = 151.0 (C-6), 143.9 (C-3a,8a), 136.3 (C-2), 135.9 (C-4,8), 131.7 (C-10), 118.5 (C-5,7), 117.4 (C-1,3), 102.2 (C-9), 40.8 (C-12,13); ESMS (ES^-) calculated for $[M-H]^-$: 196.12, found at $[C_{14}H_{14}N]^-$: 196.0.

10a 6-formylazulene.[1]

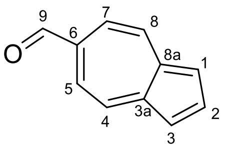

Chemical Formula: $C_{11}H_8O$
Exact Mass: 156.0575

To a solution of compound **9** (250 mg, 1.27 mmol) in THF (3 mL) and water (3 mL) was added $NaIO_4$ (813 mg, 3.80 mmol) and the mixture was stirred vigorously at RT for 1 h. The resulting green solution was filtered and EtOAc was added (20 mL). The organic phase was washed with sat. aqueous $NaHCO_3$ (3 × 10 mL), dried over $MgSO_4$ and filtered. The solvent was removed in *vacuo*. The obtained green solid was purified by column chromatography (petroleum ether:ethyl acetate 10:1) to give the pure product **10a** (138 mg, 69 %) as green crystals. **R_f** (petroleum ether:ethyl acetate 20:1): 0.28; **$UV_{\lambda max}$** (CH_3CN:H_2O): 233, 276, 282, 343 nm; **^{1}H-NMR** (400 MHz, $CDCl_3$): δ = 10.11 (s, 1H, H-9), 8.53 (dd, *J* = 10.2, 1.3 Hz, 2H, H-4,8), 8.11 (t, *J* = 3.8 Hz, 1H, H-2), 7.73 (dd, *J* = 10.2, 1.3 Hz, 2H, H-5,7), 7.51 (d, *J* = 3.8 Hz, 2H, H-1,3); **^{13}C-NMR** (101 MHz, $CDCl_3$): δ = 194.9 (C-9), 141.7 (C-6), 141.5 (C-3a,8a), 140.3 (C-2), 135.4 (C-5,7), 123.9 (C-4,8), 119.9 (C-1,3); **ES-MS (ES+):** calculated for $[M+H]^+$: 157.06, found at $[C_{11}H_8OH]^+$: 157.1.

11a 6-(hydroxymethyl)azulene.[1]

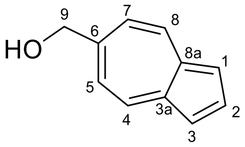

Chemical Formula: $C_{11}H_{10}O$
Exact Mass: 158.0732

To a solution of $NaBH_4$ (58.1 mg, 1.54 mmol) in methanol (1.5 mL) was added compound **10a** (120 mg, 0.77 mmol) in CH_2Cl_2 (3 mL) at 0 °C. The reaction was stirred vigorously for 30 min, quenched with aqueous sat. $NaHCO_3$ (5 mL) and extracted with CH_2Cl_2 (3 × 25 mL). The combined blue organic phases were dried over $MgSO_4$, filtered and evaporated to give pure compound **11a** (121 mg, 92 %) as a blue solid. **R_f** (petroleum ether:ethyl acetate 10:1): 0.13; **$UV_{\lambda max}$** (CH_3CN:H_2O): 228, 276, 282, 343 nm; **^{1}H-NMR** (400 MHz, $CDCl_3$): δ = 8.33 (d, *J* = 9.9 Hz, 2H, H-4,8), 7.90 (t, *J* = 3.7 Hz, 1H, H-2), 7.39 (d, *J* = 3.8 Hz, 2H, H-1,3), 7.22 (d, *J* = 9.9 Hz, 2H, H-5,7), 4.83 (s, 2H, H-9); **^{13}C-NMR** (101 MHz, $CDCl_3$): δ = 149.7 (C-6), 139.4 (C-2), 136.8 (C-3a,8a), 136.0 (C-5,7), 121.2 (C-4,8), 118.3 (C-1,3), 68.9 (C-9); **ES-MS (ES+):** calculated for $[M+H]^+$: 159.07, found at $[C_{11}H_{10}OH]^+$: 159.2.

12a 6-(chloromethyl)azulene.

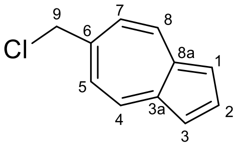

Chemical Formula: $C_{11}H_9Cl$
Exact Mass: 176.0393

A solution of alcohol **11a** (1.1 g, 6.95 mmol) in CH_2Cl_2 (10 mL) and Et_3N (1.16 mL, 8.34 mmol) was cooled to 0 °C. After cooling TsCl (1.46 g, 7.65 mmol) dissolved in CH_2Cl_2 (10 mL) was added dropwise and allowed to warm to rt. After stirring overnight the reaction mixture was quenched with cold H_2O and extracted with CH_2Cl_2 (3 × 20mL). The combined blue organic phases were washed with a sat. $NaHCO_3$ solution, dried with $MgSO_4$ and carefully evaporated *in vacuo* to give the chlorinated compound **12a** as a dark blue solid/oil. Due to instability of this compound it was used without further purification. **R_f** (petroleum ether): 0.23; **$UV_{\lambda max}$** (CH_3CN:H_2O): 233, 278, 282, 343 nm; **^{1}H-NMR** (400 MHz, $CDCl_3$): δ = 8.33 (d, *J* = 10.1 Hz, 2H, H-4,8), 7.90 (t, *J* = 3.7 Hz, 1H, H-2), 7.41 (d, *J* = 3.7 Hz, 2H, H-1,3), 7.24 (d, *J* = 10.1 Hz, 2H, H-5,7), 4.72 (s, 2H, H-9); **^{13}C-NMR** (101 MHz, $CDCl_3$): δ = 145.9 (C-6), 139.8 (C-2), 138.0 (C-3a,8a), 135.9 (C-5,7), 123.6 (C-4,8), 119.0 (C-1,3), 51.5 (C-9); **ES-MS (ES+):** calculated for $[M+H]^+$: 177.05, found at $[C_{11}H_9ClH]^+$: 177.2.

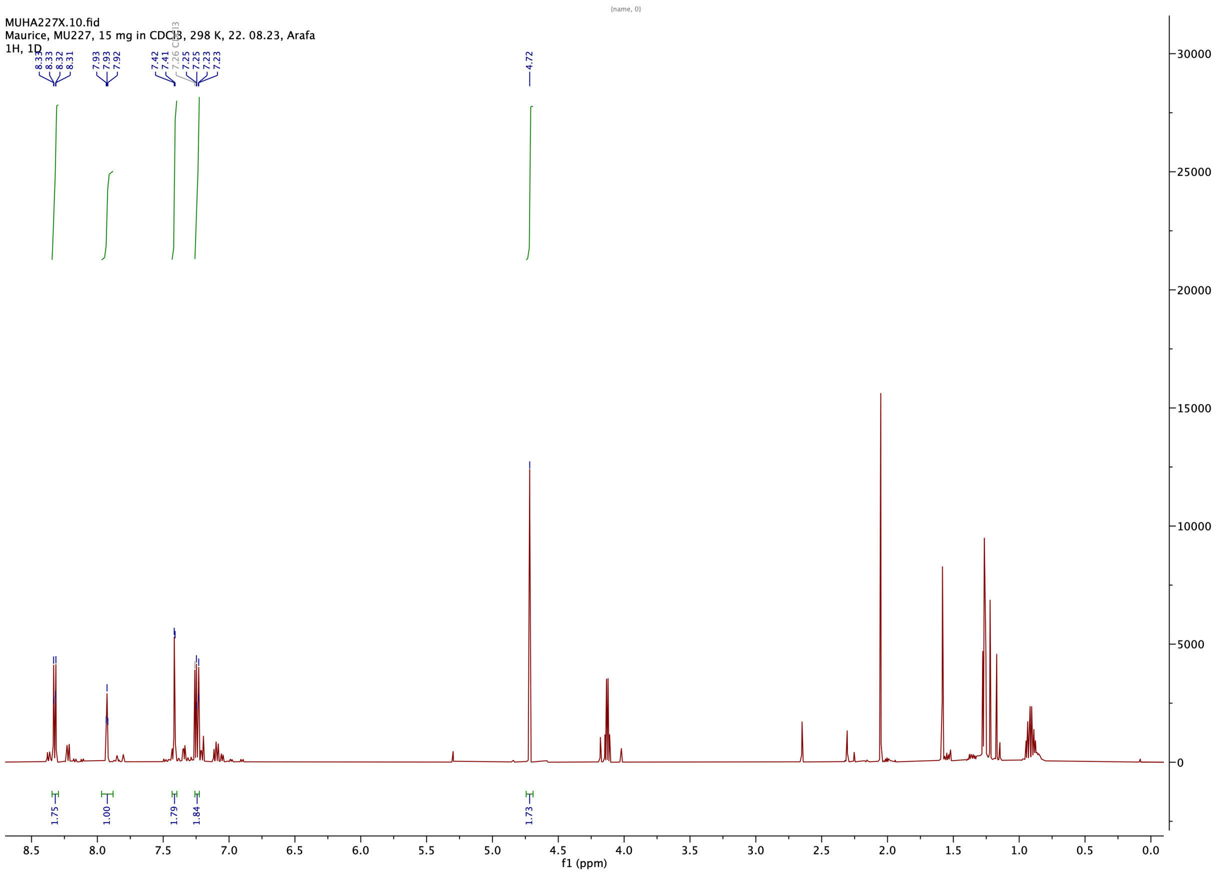

Figure S1. ^{1}H NMR of **12a** ($CDCl_3$).

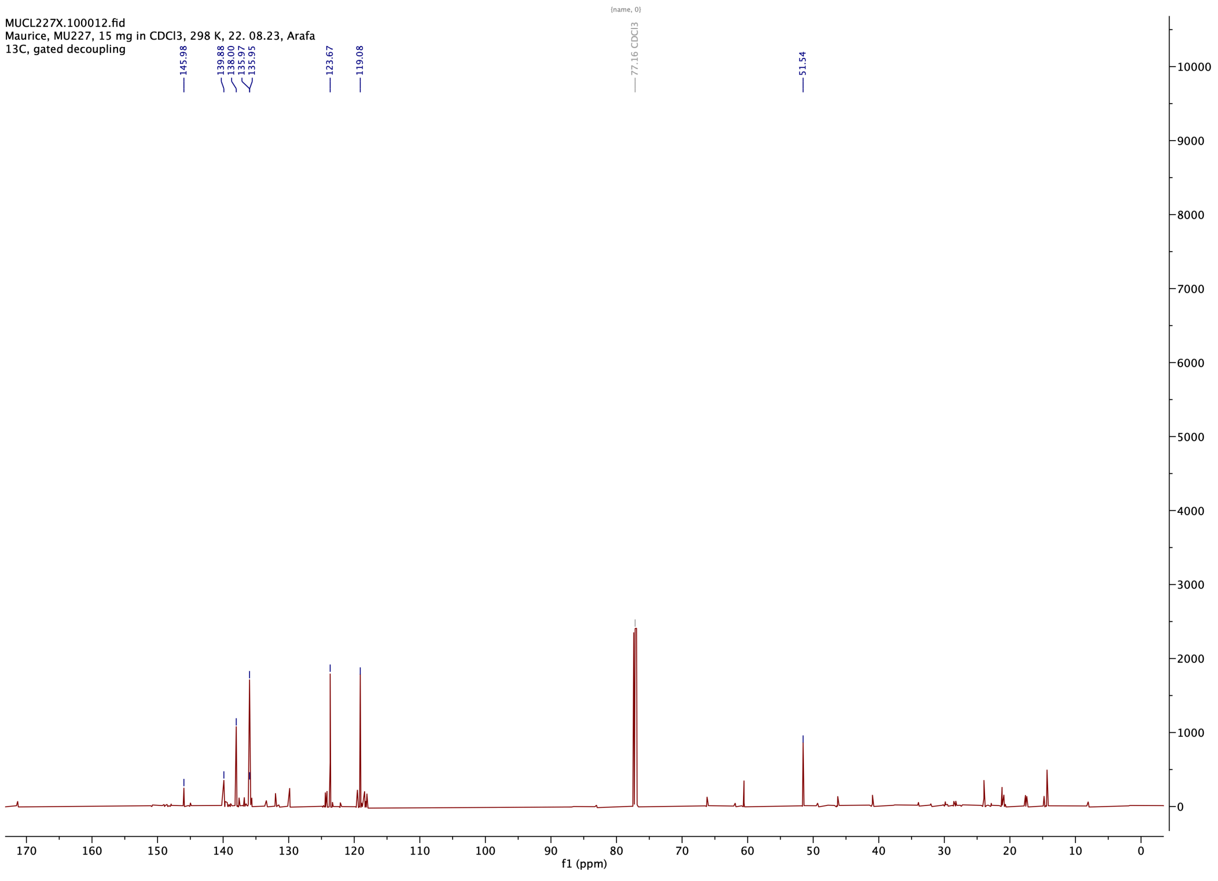

Figure S2. ^{13}C NMR of **12a** ($CDCl_3$).

13a Diethyl 2-acetamido-2-(azulen-6-ylmethyl)malonate.

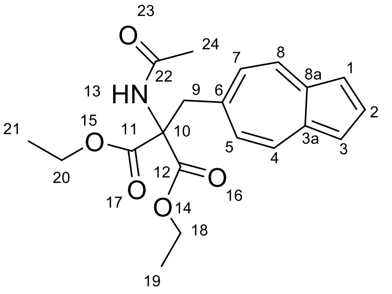

Chemical Formula: $C_{20}H_{23}NO_5$
Exact Mass: 357.1576

To a solution of NaH (60 % in mineral oil; 16.5 mg, 0.41 mmol) in DMF (4 mL) were added diethyl acetamidomalonate (89.7 mg, 0.41 mmol) in DMF (2 mL) and compound **12a** (80 mg, 0.36 mmol) in DMF (2 mL) successively. After stirring for 4 h at rt, the solvent was removed under reduced pressure and the residue was dissolved in CH_2Cl_2 (20 mL). The organic phase was washed with water (20 mL). The resulting aqueous phase was extracted with CH_2Cl_2 (10 mL) and the combined organic phases were dried over $MgSO_4$ and filtered. After removing the solvent in *vacuo*, the residue was subjected to column chromatography (petroleum ether:ethyl acetate 2:1 → 100 % ethyl acetate) to yield title compound **13a** (70 mg, 67 % over two steps). **R$_f$** (petroleum ether:ethyl acetate 2:1): 0.60; **UV$_{\lambda max}$** (CH_3CN:H_2O): 233, 276, 282, 343 nm; **^{1}H-NMR** (400 MHz, $CDCl_3$): δ = 8.20 (d, *J* = 10.5 Hz, 2H, H-4,8), 7.88 (t, *J* = 3.8 Hz, 1H, H-2), 7.36 (d, *J* = 3.8 Hz, 2H, H-1,3), 6.87 (d, *J* = 10.5 Hz, 2H, H-5,7), 6.53 (s, 1H, H-13), 4.31 (q, *J* = 7.1 Hz, 4 H, H-18,20), 3.85 (s, 2H, H-9), 2.03 (s, 3H, H-24), 1.33 (t, *J* = 7.1 Hz, 6 H, H-19,21); **^{13}C-NMR** (101 MHz, $CDCl_3$): δ = 169.3 (C-22), 167.3 (C-11,12), 145.3 (C-6), 139.2 (C-2), 136.9 (C-3a,8a), 135.2 (C-4,8), 124.8 (C-5,7), 118.2 (C-1,3), 67.5 (C-10), 62.8 (C-18,20), 43.2 (C-9), 23.0 (C-24), 14.0 (C-19,21); **ES-MS (ES+):** calculated for $[M+H]^+$: 358.41, found at $[C_{20}H_{23}NO_5H]^+$: 358.3; **HRMS (ESI):** calculated for $C_{20}H_{24}NO_5^+$ $[M+H]^+$: 358.1654; found: 358.1657.

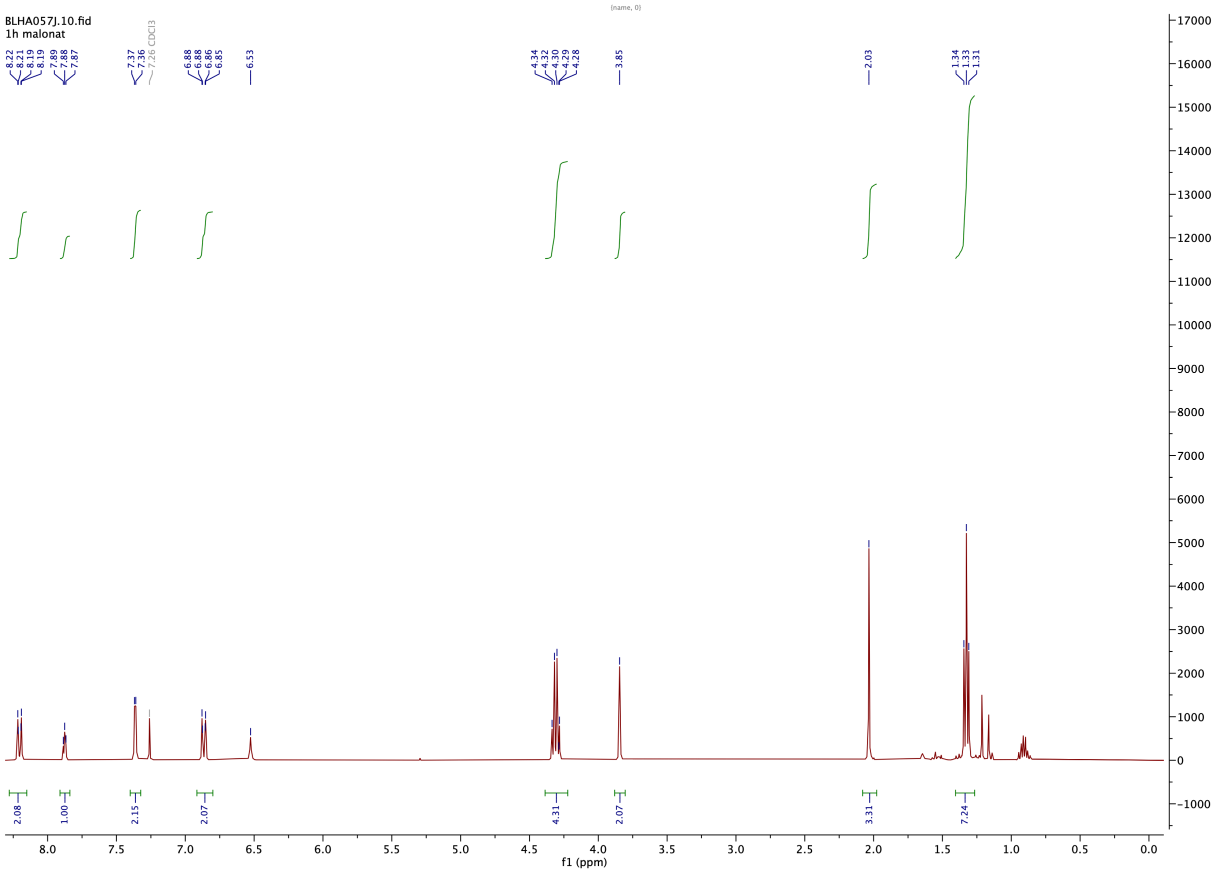

Figure S3. ^{1}H NMR of **13a** ($CDCl_3$).

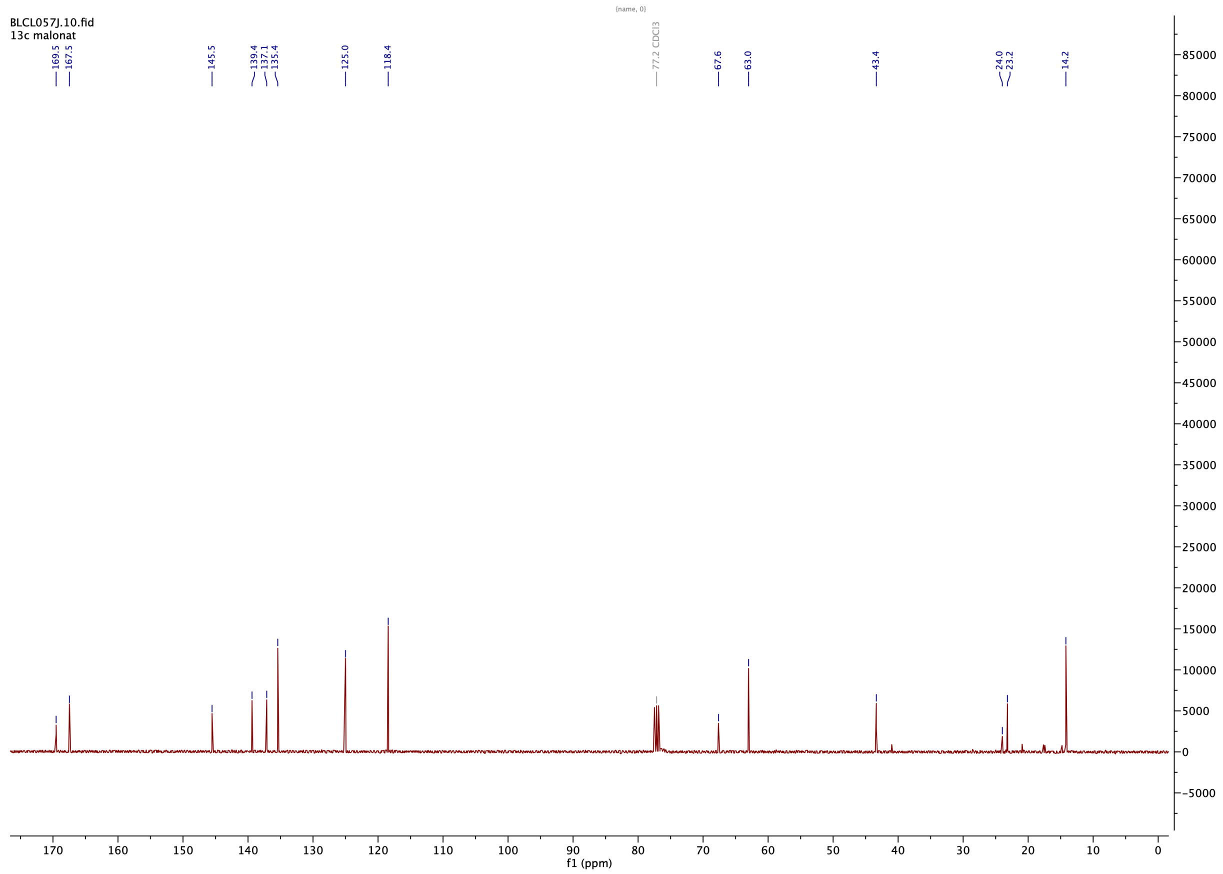

Figure S4. ^{13}C NMR of **13a** ($CDCl_3$).

14a *N*-acetyl-β-(6-azulenyl)alanine.

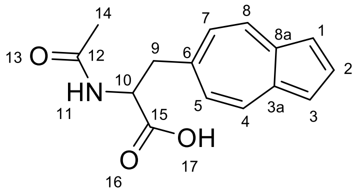

Chemical Formula: $C_{15}H_{15}NO_3$
Exact Mass: 257.1052

To a solution of compound **13a** (200 mg, 0.78 mmol) in THF/water (3.5 mL/1 mL) was added $LiOH{\cdot}H_2O$ (130 mg, 3.1 mmol) at rt. The resulting purple solution was stirred at 50 °C for 36h and the THF was removed under reduced pressure. The blue residue was dissolved in water (10 mL) acidified to pH 3, extracted with ethyl acetate (3 × 20 mL) and dried with $MgSO_4$. The solvent was evaporated, and the residue subjected to column chromatography (petroleum ether:ethyl acetate 3:1 → 1:1 with all solvents containing 0.5 % formic acid) to yield title compound **14a** (135 mg, 93 %); **R$_f$** (petroleum ether:ethyl acetate 1:1 + 1 % FA): 0.16; **UV$_{\lambda max}$** (CH_3CN:H_2O): 233, 276, 282, 343 nm; **^{1}H-NMR** (400 MHz, $CDCl_3$): δ = 8.32 (d, *J* = 10.4 Hz, 2H, H-4,8), 7.81 (t, *J* = 3.7 Hz, 1H, H-2), 7.35 (d, *J* = 3.8 Hz, 2H, H-1,3), 7.20 (d, *J* = 10.0 Hz, 2H, H-5,7), 4.83 (t, *J* = 5.5 Hz, 1H, H-10), 3.40 (dd, *J* = 5.6 Hz, 1H, H-9a), 3.20 (dd, *J* = 8.3 Hz, 1H, H-9b), 1.87 (s, 3 H, H-15); **^{13}C-NMR** (101 MHz, $CDCl_3$): δ = 170.9 (C-11), 168.3 (C-13), 146.9 (C-6), 138.3 (C-2), 135.1 (C-3a,8a), 134.5 (C-5,7), 123.6 (C-4,8), 116.9 (C-1,3), 53.0 (C-10), 42.0 (C-9), 20.7 (C-15). **ES-MS (ES+):** calculated for $[M+H]^+$: 258.11, found at $[C_{15}H_{15}NO_3H]^+$: 258.2; **HRMS (ESI):** calculated for $C_{15}H_{14}NO_3^-$ $[M-H]^-$: 256.0974; found: 256.0975.

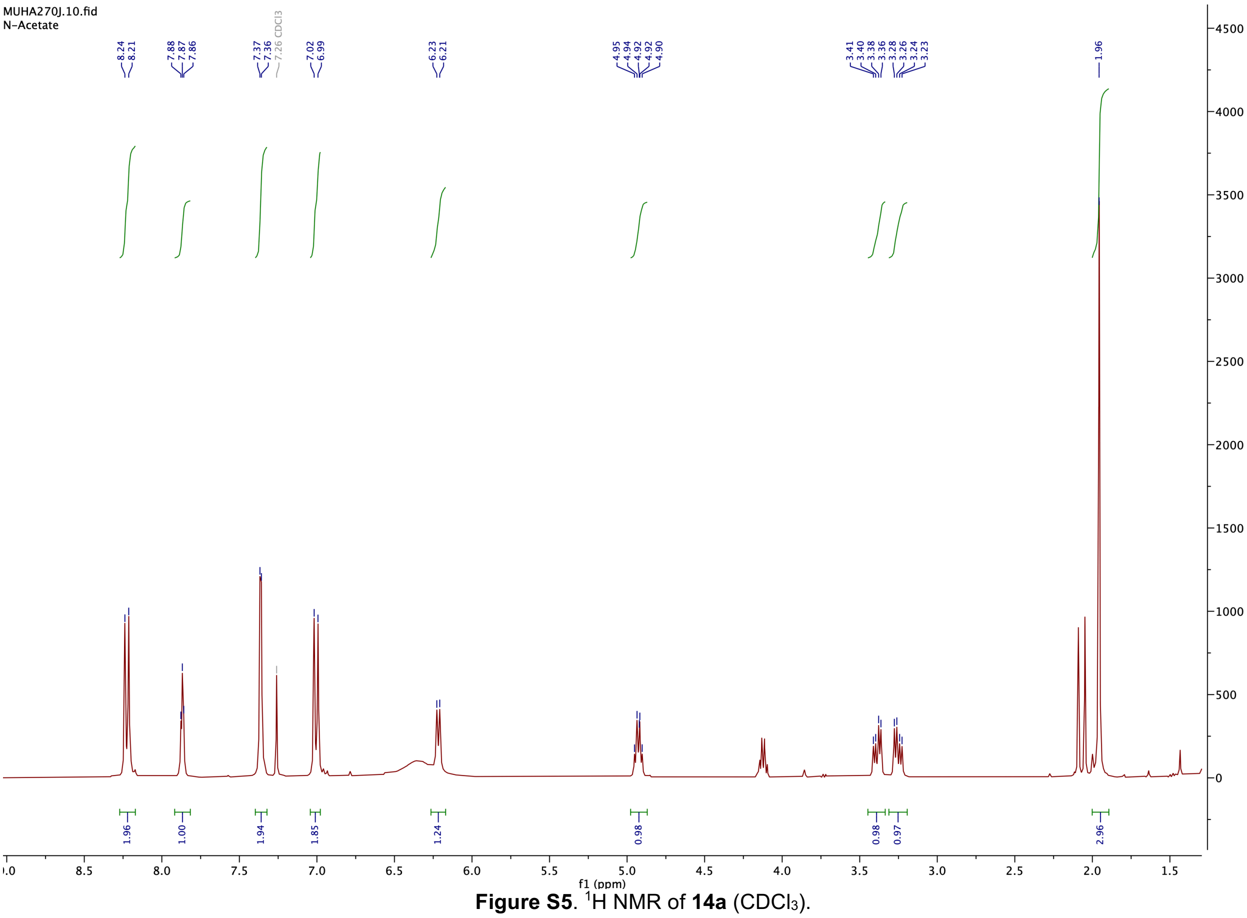

Figure S5. ^{1}H NMR of **14a** ($CDCl_3$).

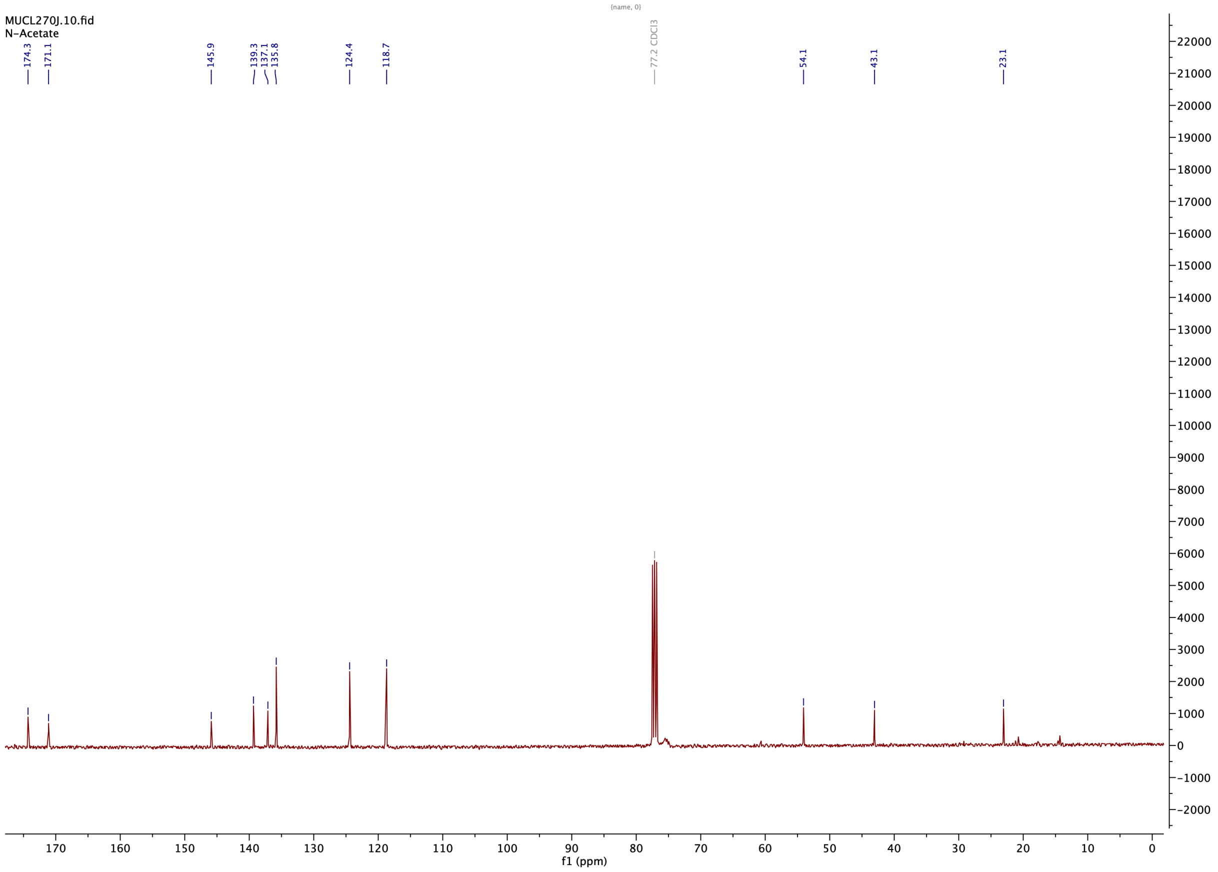

Figure S6. ^{13}C NMR of **14a** ($CDCl_3$).

6a *L*-β-(6-azulenyl)alanine

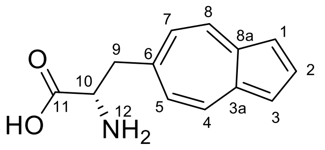

Chemical Formula: $C_{13}H_{13}NO_2$
Exact Mass: 215.0946

To a solution of compound **14a** (50 mg, 0.19 mmol) in potassium phosphate buffer (0.1M, pH 7.5, 13.5 mL) was added porcine kidney acylase (4 mg, 0.41 mmol; Activity: 258 untis per mg) and the resulting blue mixture was stirred for five days at 37 °C. The solution was acidified to pH 3 and filtered into a separating funnel. The aqueous solution was extracted with CH_2Cl_2 (4 × 20 mL) to remove unreacted starting material. The aqueous phase was then neutralised and subjected to HPLC purification to give a blue solid (16 mg, 41%). **UV$_{\lambda max}$** (MeOH): 233, 276, 282, 343 nm; **^{1}H-NMR** (600 MHz, DMSO-d$_6$): δ = 8.31 (d, *J* = 9.9 Hz, 2H, H-4,8), 7.81 (t, *J* = 3.7 Hz, 1H, H-2), 7.34 (d, *J* = 3.7 Hz, 2H, H-5,7), 7.20 (d, *J* = 9.9 Hz, 2H, H-1,3), 3.45 (dd, *J* = 8.8, 4.5 Hz, 1H, H-10), 3.39 (m, 1H, H-9a) 2.98 (dd, *J* = 13.6, 8.7 Hz, 1H, H-9b); **^{13}C-NMR** (150 MHz, DMSO-d$_6$): δ = 169.1 (C-11), 149.1 (C-6), 138.7 (C-2), 136.0 (C-3a,8a), 135.8 (C-5,7), 124.7 (C-4,8), 117.6 (C-1,3), 56.6 (C-10), 43.2 (C-9); **ES-MS (ES+):** calculated for $[M+H]^+$: 216.09, found at $[C_{13}H_{13}NO_2H]^+$: 216.2; **HRMS (ESI):** calculated for $C_{13}H_{12}NO_2^-$ $[M-H]^-$: 214.0868; found: 214.0861

To access a racemic mixture of (6-azuleno)alanine, *N*-acetyl-β-(6-azuleno)alanine **13a** (20 mg, 0.078 mmol) was dissolved in 2M H_2SO_4 (1mL) and heated to 100 °C for 30 min. After completion, the crude mixture was filtered through celite and directly submitted to HPLC purification to give a blue/brownish solid (< 1 mg). This was used for the *ee* determination without further characterisation.

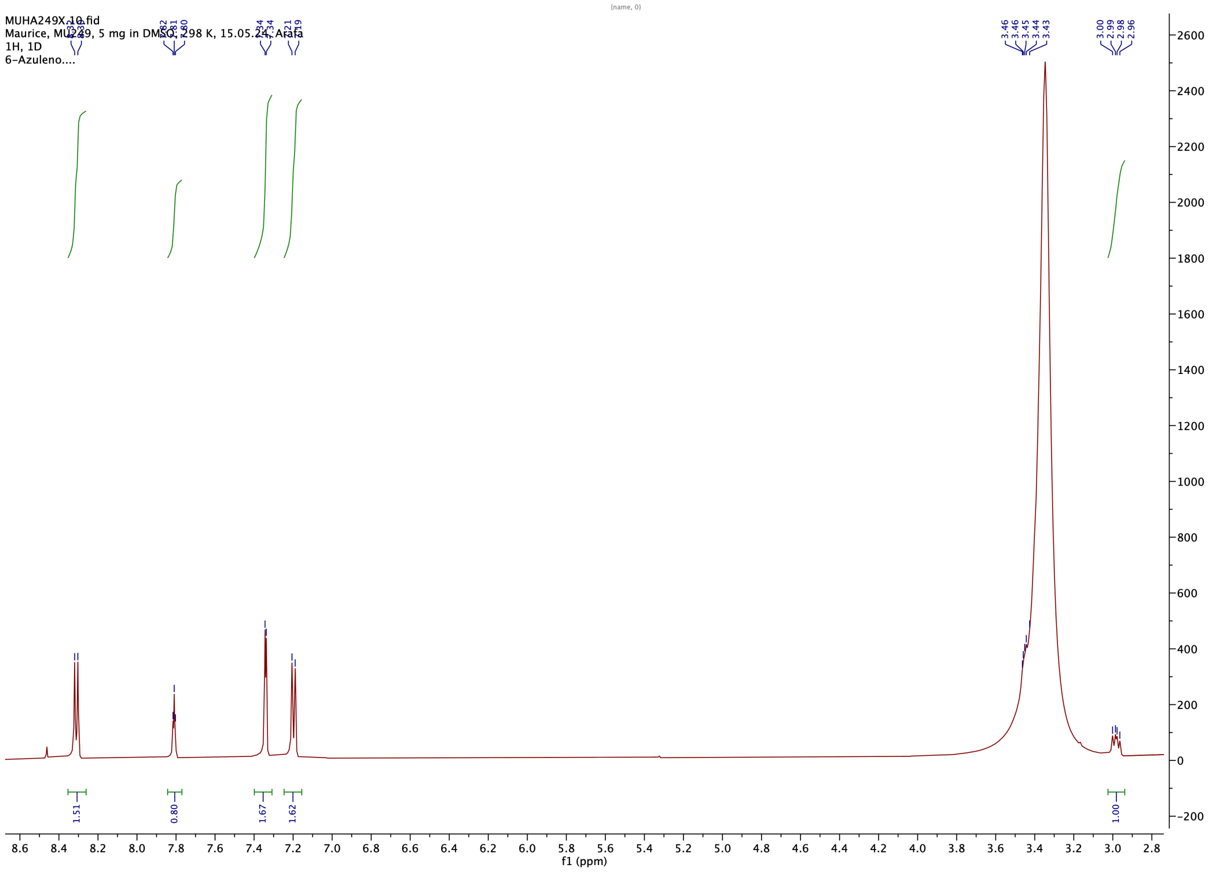

Figure S7. ^{1}H NMR of **6a** (DMSO-d$_6$).

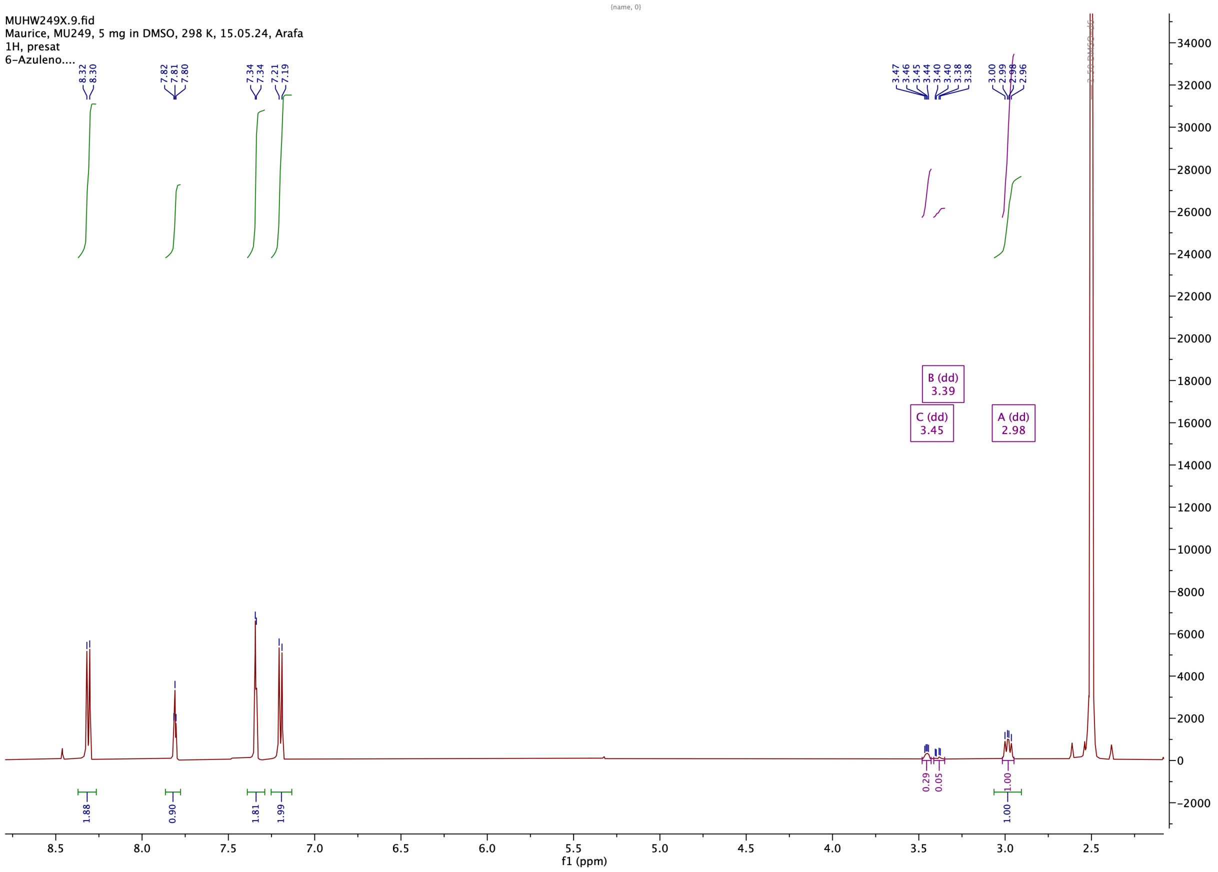

Figure S8. ^{1}H NMR of **6a** (DMSO-d$_6$, water supression).

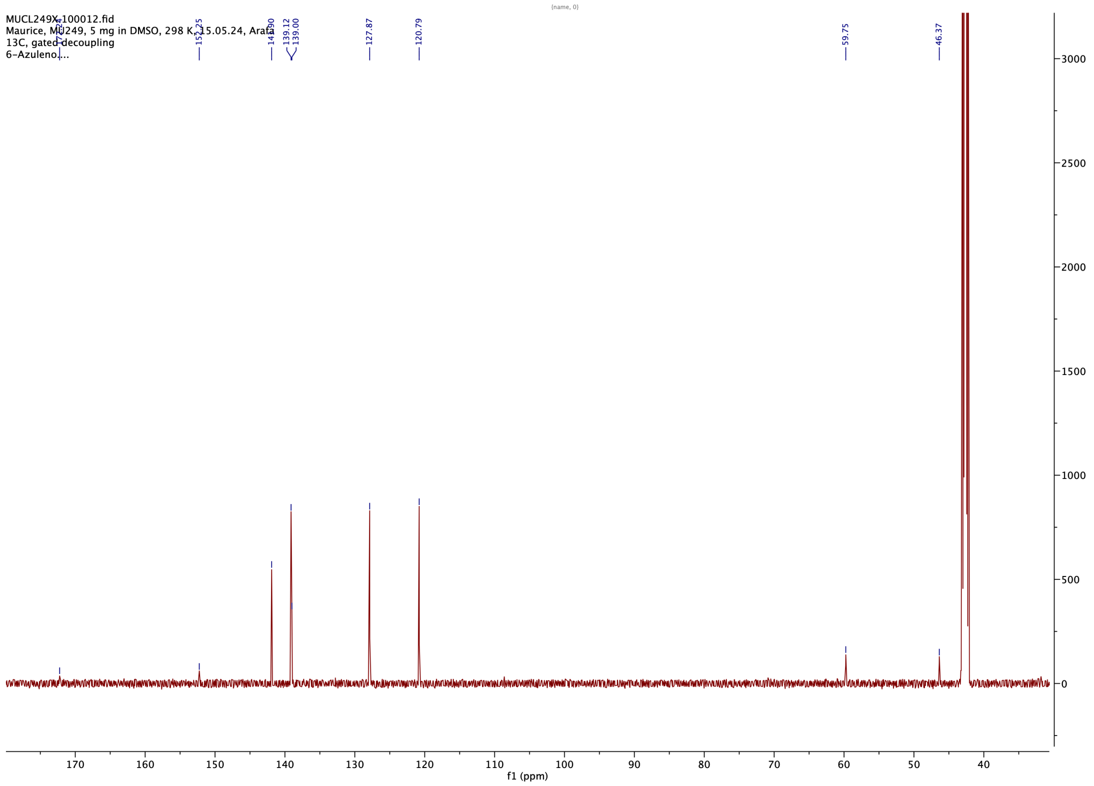

Figure S9. ^{13}C NMR of **6a** (DMSO-d$_6$).

4-(Diethoxymethyl)pyridine[2]

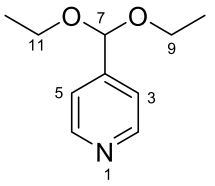

At room temperature, 4-pyridinecarboxaldehyde (2.1 g, 20 mmol), triethyl orthoformate (6 g, 40 mmol), and TsOH·H_2O (76 mg, 2 mol %) were dissolved in anhydrous ethanol (20 mL). The reaction mixture was refluxed in ethanol for 6 h in an oil bath. After removal of the solvent, the residue was chromatographed on a silica gel column eluting with a mixture of petroleum ether and ethyl acetate (PE/EA = 10:1) to give 4-(diethoxymethyl)pyridine as colorless oil (3.37 g, 93%). **^{1}H NMR** (400 MHz, $CDCl_3$) δ 8.61 (d, *J* = 6.0 Hz, 2H, H-2, 6), 7.39 (d, *J* = 5.4 Hz, 2H, H-3, 5), 5.49 (s, 1H, H-7), 3.66 – 3.48 (m, 4H, H-9, 11), 1.24 (t, *J* = 7.1 Hz, 6H, H-10, 12).

18 *N*-butyl-4-(diethoxymethyl)pyridinium bromide[2]

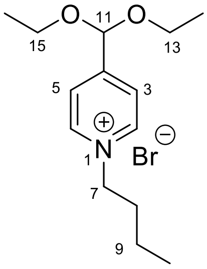

4-(Diethoxymethyl)pyridine (4.0 g, 22.0 mmol) was dissolved in dry ethanol (10 ml) and 1-bromobutane (4.56 g, 33.0 mmol) The mixture was heated at reflux for 16 h. The ethanol excess 1-bromobutane were evaporated under reduced pressure. The resulting yellow oil was left under high vacuum to remove any remaining solvent to give *N*-butyl-4-(diethoxymethyl)pyridinium bromide **18**, (6.0 g, 85%). **^{1}H NMR** (400 MHz, $CDCl_3$) δ 9.60 (d, *J* = 6.5 Hz, 2H, H-2, 6), 8.09 (d, *J* = 6.3 Hz, 2H, H-3, 5), 5.62 (s, 1H, H-11), 5.02 (t, *J* = 7.4 Hz, 2H, H-7), 3.59 (qd, *J* = 7.0, 2.3 Hz, 4H, H-13, 15), 2.08 – 1.96 (m, 2H, H-8), 1.47 – 1.33 (m, 2H, H-9), 1.23 (td, *J* = 7.0, 3.2 Hz, 6H, H-14, 16), 0.97 – 0.81 (t, 3H, H-10).

19 6-(diethoxymethyl)-1-methylazulene

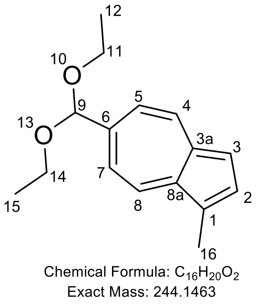

Freshly distilled 2-methylcyclopenta-1,3-diene (2.42 g, 40 mmol) was added dropwise over 30 min to NaH (1.6 g, 42 mmol, 60 % in mineral oil) in THF (70 ml) at 0 °C and then allowed to warm to 20 °C. *N*-butyl-4-(diethoxymethyl)pyridinium bromide **18** (17.5 g, 55.0 mmol) dissolved in THF (50 ml) was then added to the pinkish solution which went dark red and then brown. The mixture was heated at reflux for 3 h, a blue spot being indicated by TLC. THF was evaporated under reduced pressure to leave a dark green oil that was purified by column chromatography using petrol ether (bp 40–60°C) as eluent gave 6-(diethoxymethyl)-1-methylazulene **19** as a blue oil (4.3 g, 44%). **R_f** (petroleum ether:ethyl acetate 10:1): 0.60; **$UV_{\lambda max}$** (CH_3CN:H_2O): 229, 281, 286, 351 nm; **^{1}H NMR** (400 MHz, $CDCl_3$) δ 8.33 (dd, *J* = 10.0, 1.3 Hz, 2H, H-5, 7), 7.83 (d, *J* = 3.7 Hz, 1H, H-2), 7.42 – 7.32 (overlapped, 3H, H-3, 4, 8), 5.55 (d, 1H, H-2), 3.81 – 3.62 (m, 4H, H-11, 14), 2.76 (s, 3H, H-16), 1.36 (t, *J* = 7.1 Hz, 6H, H-12, 15); **^{13}C NMR** (101 MHz, $CDCl_3$) δ 12.8 (C-16), 15.3 (C-12,15), 61.9 (C-11, 14), 104.7 (C-9), 116.7 (C-3), 119.9 (C-7), 120.6 (C-5), 126.5 (C-1), 133.1 (C-8), 135.5 (C-4), 136.1 (C-8a), 138.5 (C-2), 140.4 (C-3a), 147.2 (C-6); **ES-MS (ES+):** calculated for $[M+H]^+$: 245.33, found at $[C_{16}H_{20}O_2H]^+$: 245.2; **HRMS (ESI):** calculated for $C_{16}H_{20}O_2^+$ $[M+H]^+$: 245.1536; found: 245.1619.

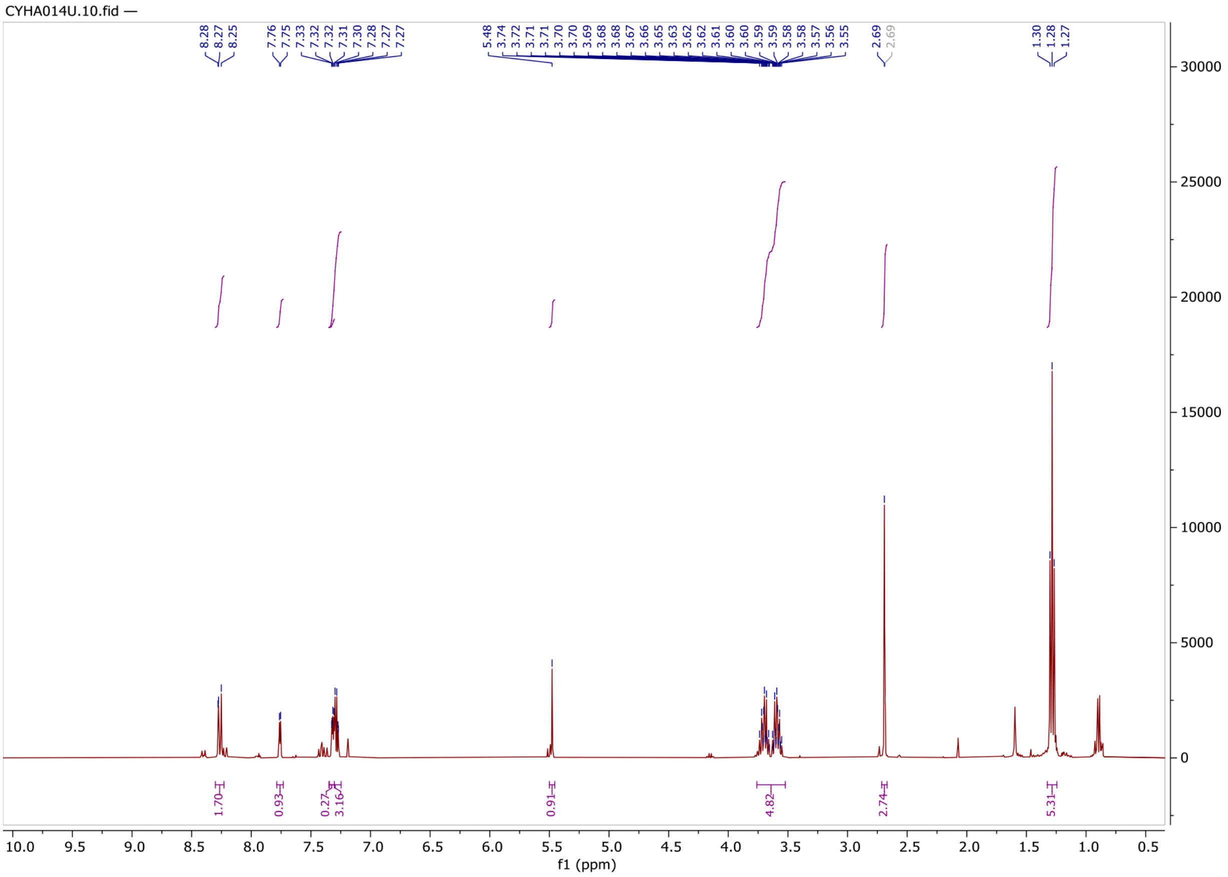

Figure S10. ^{1}H NMR of **19** ($CDCl_3$).

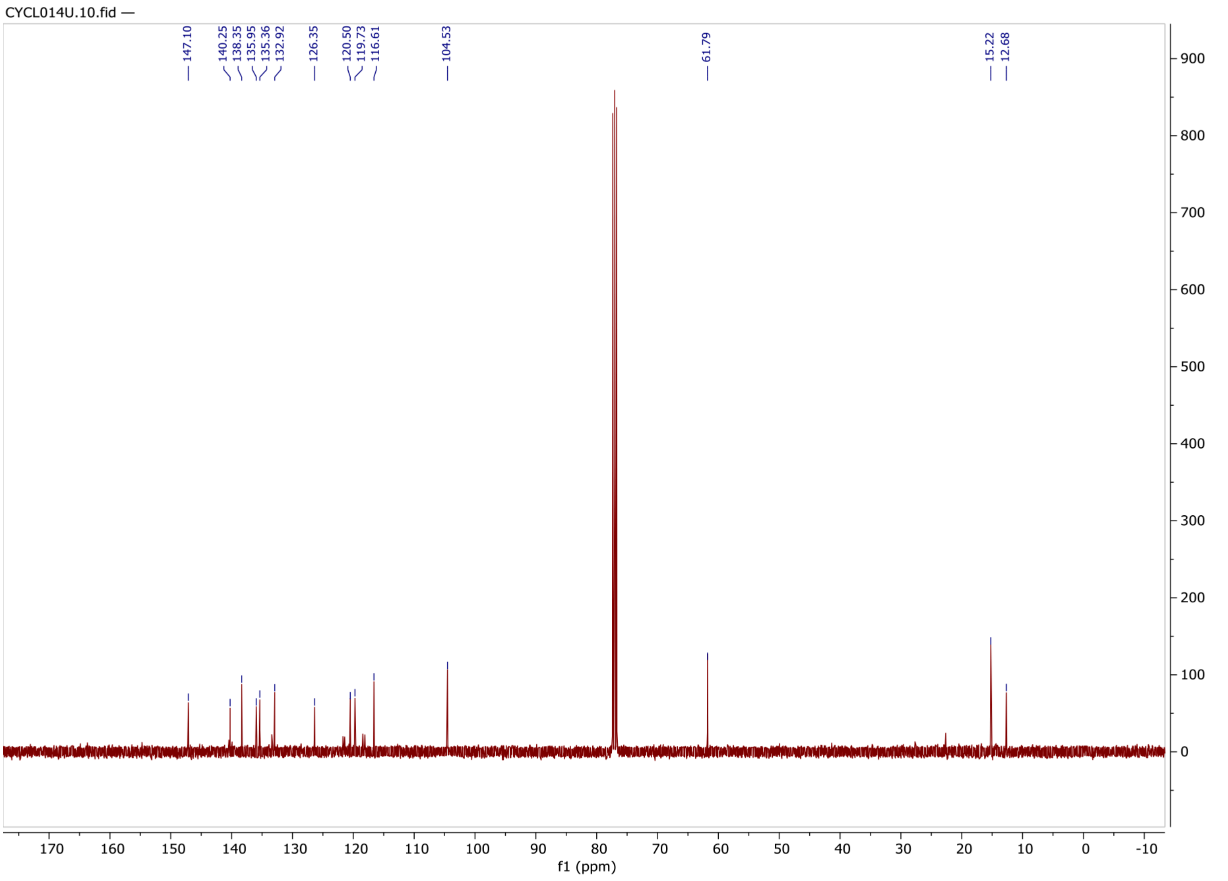

Figure S11. ^{13}C NMR of **19** ($CDCl_3$).

10b 6-formyl-1-methylazulene

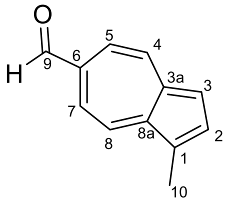

Chemical Formula: $C_{12}H_{10}O$
Exact Mass: 170.0732

To a solution of Compond **19** (2.45g, 10 mmol) in methanol (10 mL) was added 1M HCl (10 mL) at 0 °C. The reaction was stirred for 30 min, quenched with 5 M $NaHCO_3$ and extracted with CH_2Cl_2 (3 × 25 mL). The combined blue organic phases were dried with $MgSO_4$. After removal of the solvent, the residue was chromatographed on a silica gel column eluting with a mixture of petroleum ether and ethyl acetate (PE/EA = 5:1) to give compound **10b** (1.4g, 81%) as a green oil. **R$_f$** (petroleum ether:ethyl acetate 10:1): 0.45; **UV$_{λmax}$** (CH_3CN:H_2O): 230, 281, 286, 350 nm; **^{1}H NMR** (400 MHz, MeOD) δ 9.98 (s, 1H, H-9), 8.36 (d, *J* = 2.3 Hz, 1H, H-5), 8.33 (d, *J* = 2.0 Hz, 1H, H-7), 7.88 (d, *J* = 3.8 Hz, 1H, H-2), 7.55 (d, *J* = 3.1 Hz, 1H, H-4), 7.57 – 7.50 (m, 1H, H-8), 7.37 (d, *J* = 3.8 Hz, 1H, H-3), 2.63 (s, 3H, H-10); **^{13}C NMR** (101 MHz, MeOD) δ 12.6 (C-10), 119.5 (C-3), 120.8 (C-7), 121.2 (C-5), 122.4 (C-7), 124.4 (C-8), 125.0 (C-4), 133.3 (C-8a), 133.7 (C-2), 135.6 (C-3a), 143.2 (C-6), 196.6 (C-9); **ES-MS (ES+):** calculated for $[M+H]^+$: 171.1, found at $[C_{12}H_{10}OH]^+$: 171.3; **HRMS (ESI):** calculated for $C_{12}H_{10}O^+$ $[M+H]^+$: 171.0804; found: 171.1643.

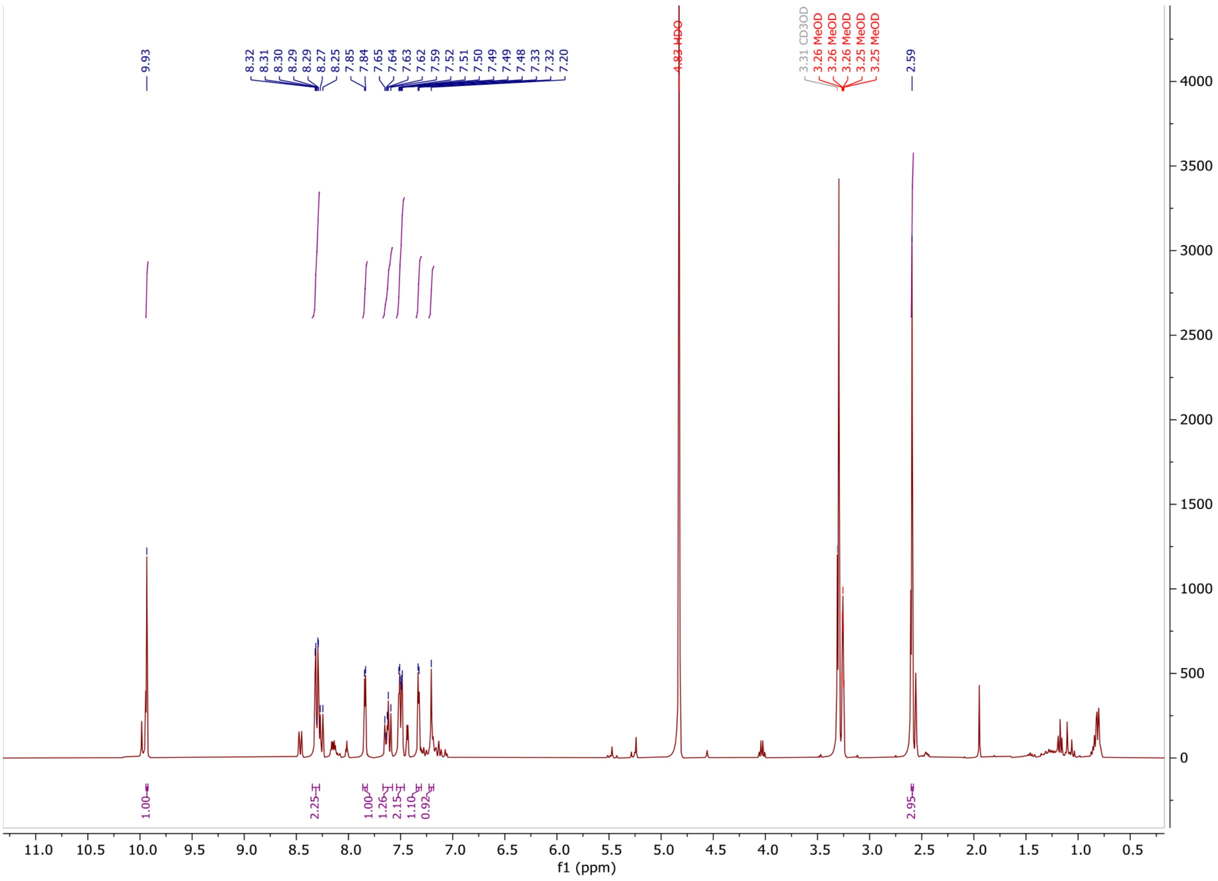

Figure S12. ^{1}H NMR of **10b** (methanol-d$_4$).

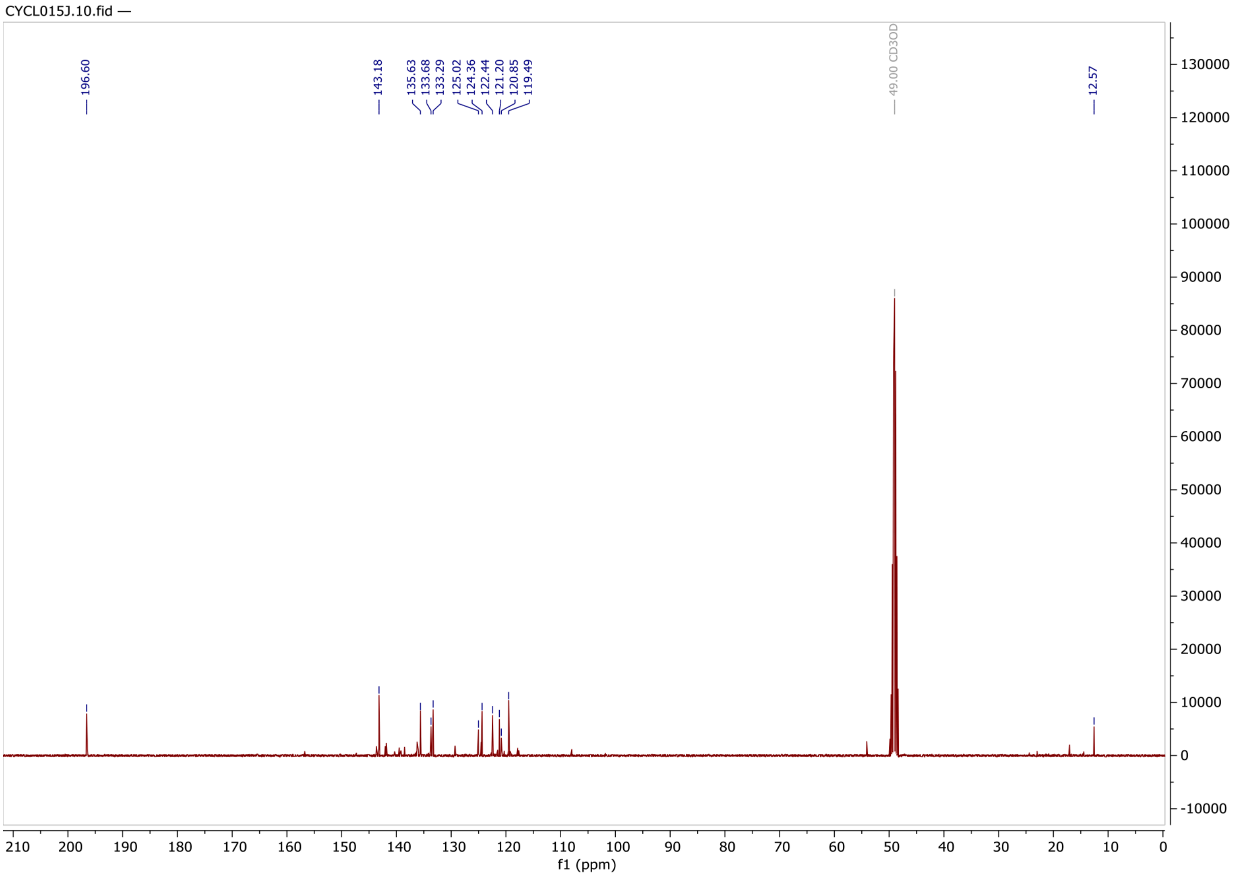

Figure S13. ^{13}C NMR of **10b** (methanol-d$_4$).

11b 6-(hydroxymethyl)-1-methylazulene

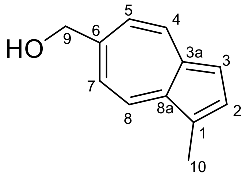

Chemical Formula: $C_{12}H_{12}O$
Exact Mass: 172.0888

To a solution of $NaBH_4$ (75.5 mg, 2.0 mmol) in methanol (1.5 mL) was added compound **10b** (170 mg, 1.0 mmol) in CH_2Cl_2 (3 mL) at 0 °C. The reaction was stirred vigorously for 30 min, quenched with 5 mL of $NaHCO_3$ and extracted with CH_2Cl_2 (3 × 25 mL). The combined blue organic phases were dried with $MgSO_4$ and evaporated to give pure compound **11b** (154.6 mg, 92 %) as a blue solid. **R$_f$** (petroleum ether:ethyl acetate 5:1): 0.35; **UV$_{\lambda max}$** (CH_3CN:H_2O): 229, 281, 286, 350 nm; **R$_f$** (petroleum ether:ethyl acetate 5:1): 0.35; **UV$_{\lambda max}$** (CH_3CN:H_2O): 229, 281, 286, 350 nm; **^{1}H NMR** (600 MHz, $CDCl_3$) δ 8.21 (dd, *J* = 9.7, 1.4 Hz, 2H, H-5, 7), 7.71 (d, *J* = 3.7 Hz, 1H, H-2), 7.29 (t, *J* = 2.9 Hz, 1H, H-3), 7.12 (t, *J* = 11.0 Hz, 2H, H-4, 8), 4.82 (d, *J* = 4.3 Hz, 2H, H-9), 2.67 (s, 3H, H-10), 1.91 (d, *J* = 6.5 Hz, 1H, H-11); **^{13}C NMR** (150 MHz, $CDCl_3$) δ 12.8 (C-10), 69.2 (C-9), 117.1 (C-3), 119.8 (C-7), 120.5 (C-5), 120.6 (C-1), 121.9 (C-8), 133.3 (C-4), 133.8 (C-8a), 135.7 (C-2), 138.0 (C-3a), 138.7 (C-6); **ES-MS (ES+):** calculated for $[M+H]^+$: 173.22, found at $[C_{12}H_{12}OH]^+$: 173.2; **HRMS (ESI):** calculated for $C_{12}H_{12}O^+$ $[M+H]^+$: 173.0961; found: 173.0911.

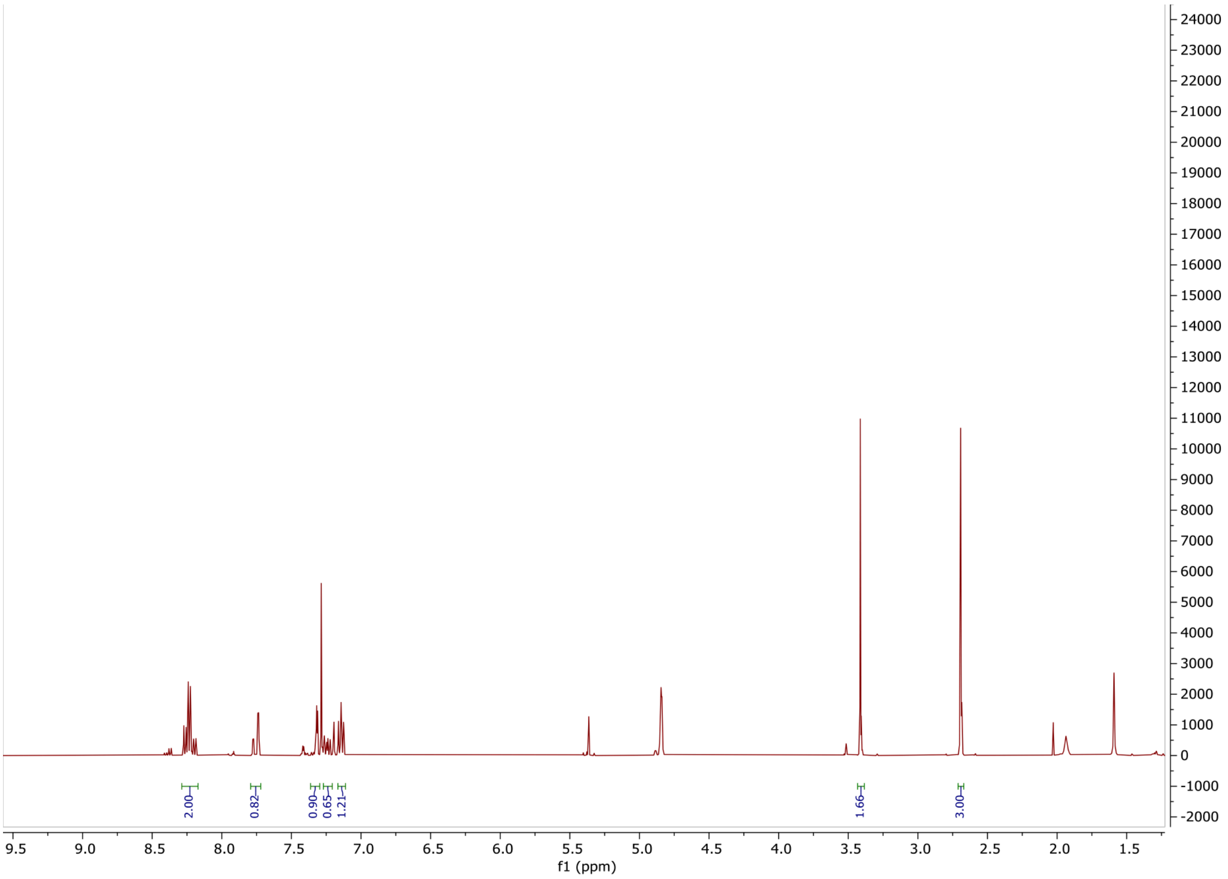

Figure S14. ^{1}H NMR of **11b** ($CDCl_3$).

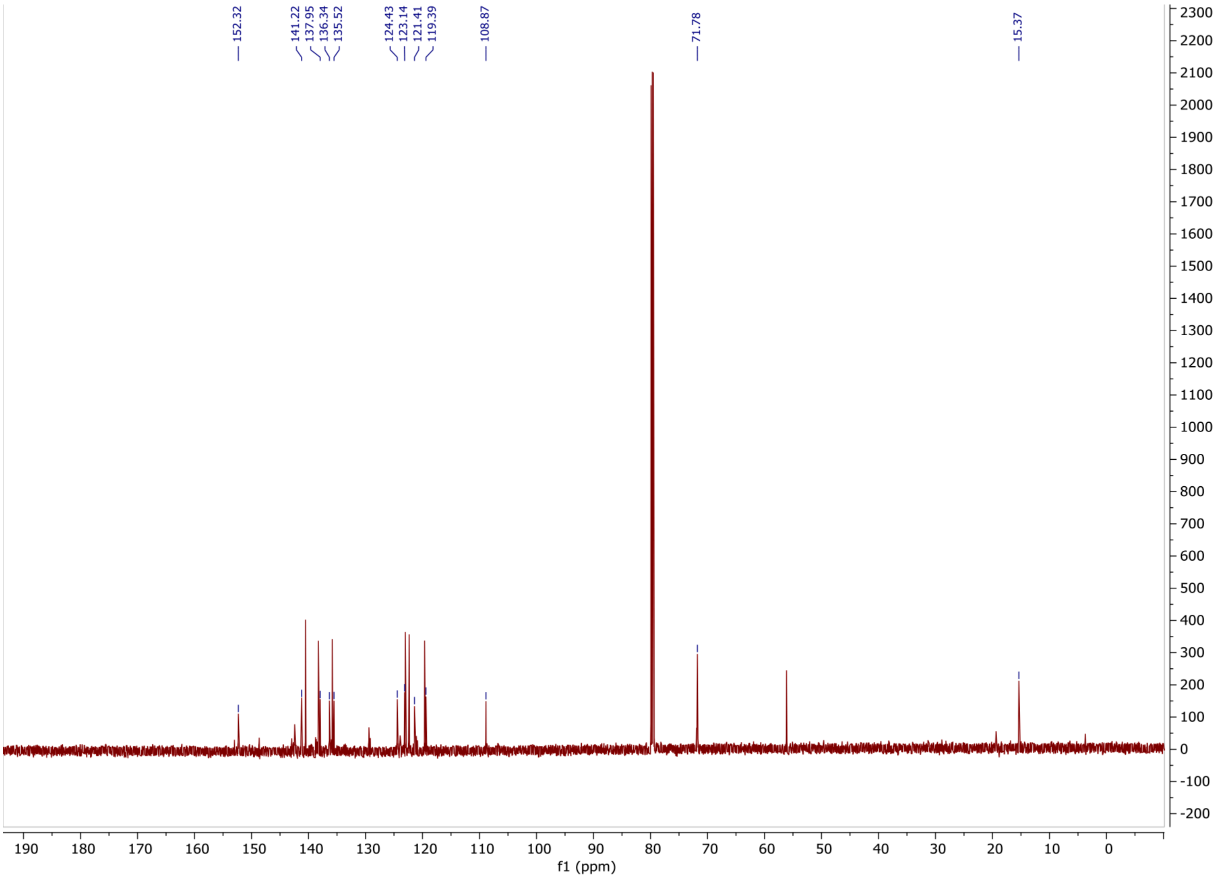

Figure S15. ^{13}C NMR of **11b** ($CDCl_3$).

12b **6-(bromomethyl)-1-methylazulene**

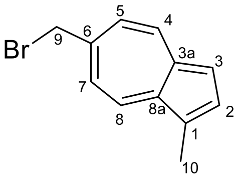

Bromination was achieved using the method of Takayusu and Nitta.[3] To a stirred solution of compound **11b** (100 mg, 0.6 mmol) and PPh_3 (262 mg, 1.0 mmol) in dry CH_2Cl_2 (10 mL) was added CBr_4 (265 mg, 0.80 mmol) at rt, and the mixture was stirred vigorously for 30 min. The reaction mixture was then chromatographed on flash column using pure CH_2Cl_2 as eluent to give compound **12b** as dark blue oil that was used without further purification. **R$_f$** (DCM): 0.75; **UV$_{\lambda max}$** (CH_3CN:H_2O): 223, 281, 287, 349 nm; **^{1}H NMR** (600 MHz, $CDCl_3$) δ 8.16 (dd, *J* = 9.8, 6.2 Hz, 2H, H-5,7), 7.73 (d, 1H, H-2), 7.26 – 7.18 (d, 1H, H-3), 7.11 (ddd, *J* = 16.0, 9.7, 1.6 Hz, 2H, H-4, 8), 4.61 (s, 2H, H-9), 2.64 (s, 3H, H-10); **^{13}C NMR** (150 MHz, $CDCl_3$) δ 12.8 (C-10), 51.1 (C-9), 117.6 (C-3), 119.4 (C-7), 122.4 (C-5), 123.2 (C-1), 124.4 (C-8), 133.0 (C-4), 133.4 (C-8a), 135.4 (C-2), 139.1 (C-3a), 140.4 (C-6); **ES-MS (ES+):** calculated for $[M+H]^+$: 235.12, found at $[C_{12}H_{11}BrH]^+$: 235.1.

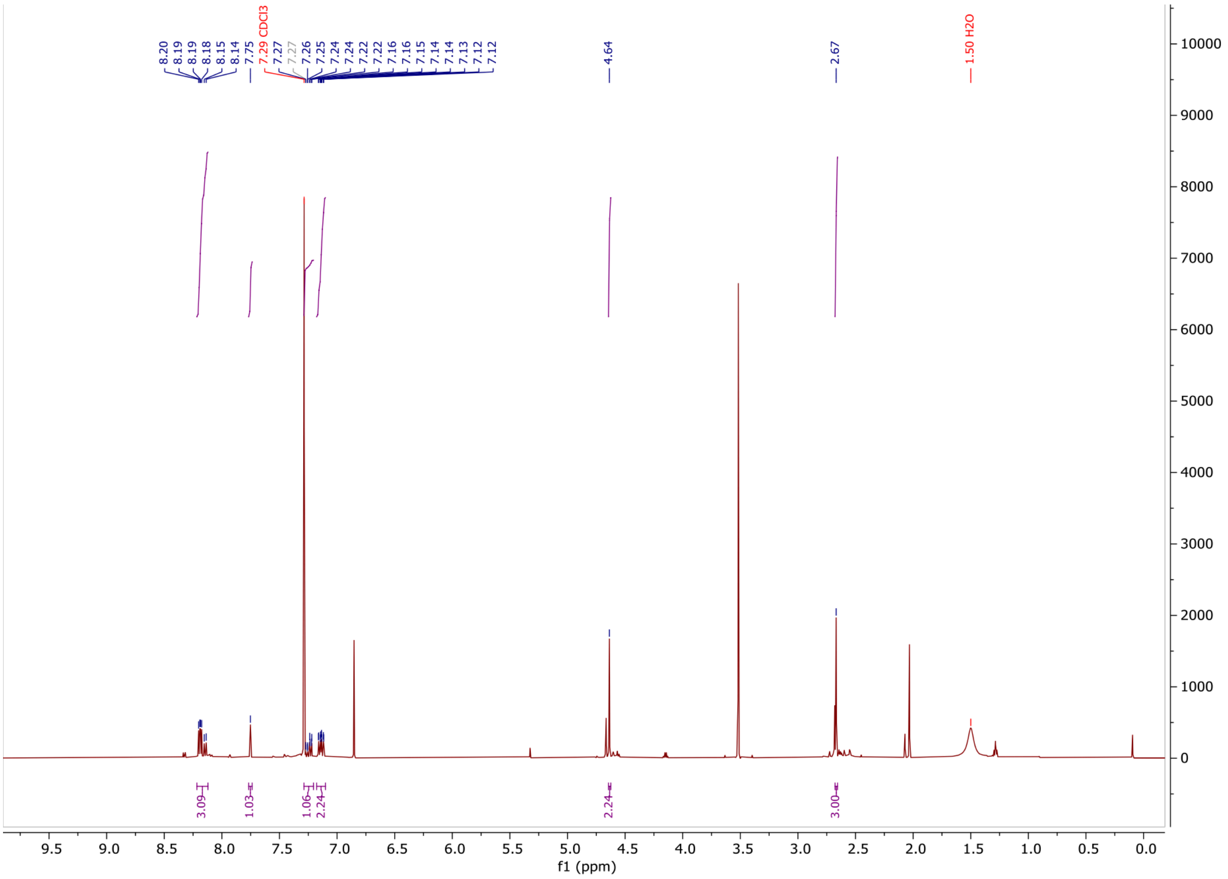

Figure S16. ^{1}H NMR of **12b** ($CDCl_3$).

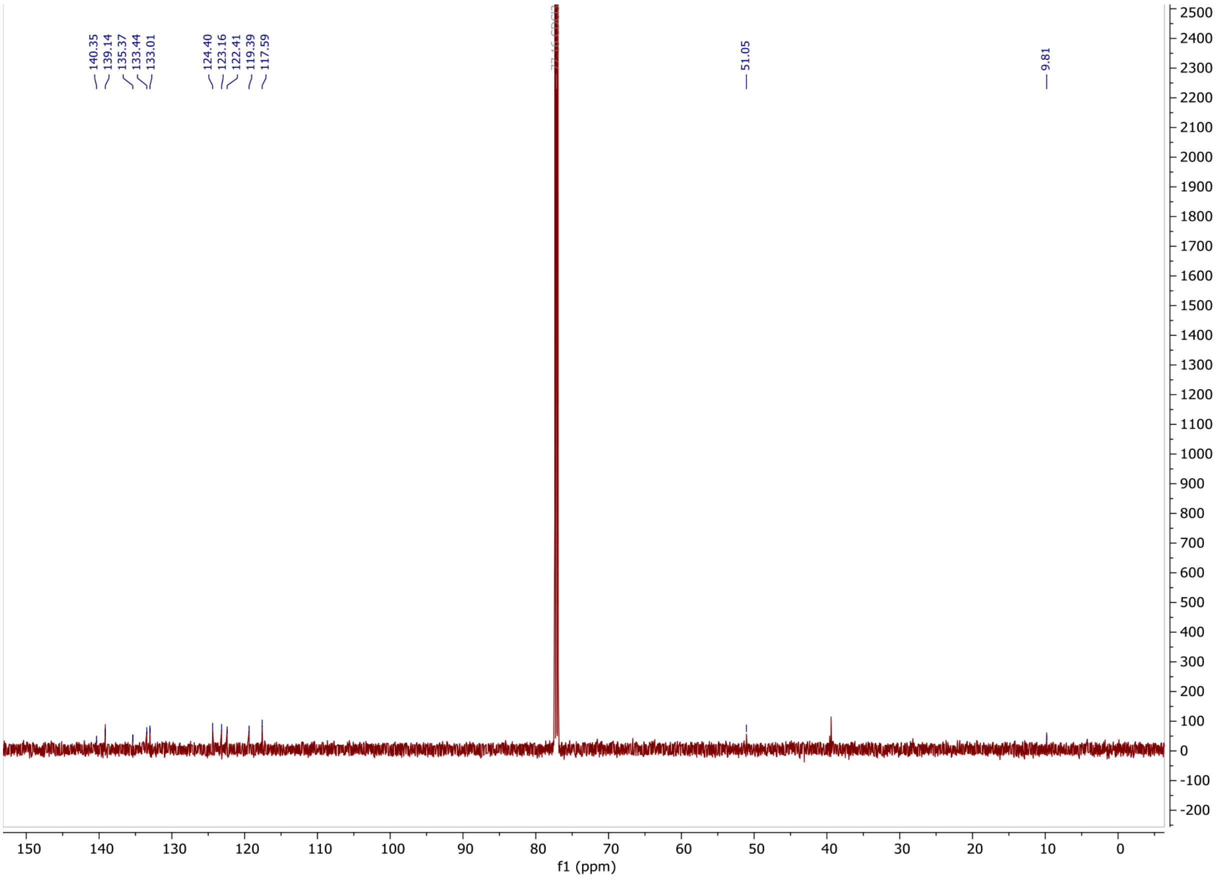

Figure S17. ^{13}C NMR of **12b** ($CDCl_3$).

13b diethyl 2-acetamido-2-((1-methylazulen-6-yl)methyl)malonate

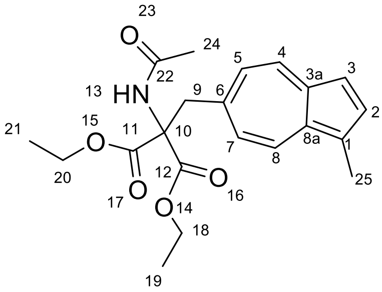

Chemical Formula: $C_{21}H_{25}NO_5$
Exact Mass: 371.1733

To a solution of NaH (160 mg, 4.0 mmol, 60 % in mineral oil) in DMF (10 mL) were added diethyl acetamidomalonate (900 mg, 4.0 mmol) in DMF (10 mL) and compound **12b** (823 mg, 3.5 mmol) in CH_2Cl_2 (8 mL) successively. After stirring for 4 h at rt, the solvent was removed under reduced pressure and the residue was dissolved in CH_2Cl_2 (50 mL). The organic phase was washed with water (50 mL), the aqueous phase was extracted with CH_2Cl_2 (50 mL) and the combined organic phases were dried over $MgSO_4$. After evaporating to dryness, the residue was subjected to column chromatography (petroleum ether:ethyl acetate 10:1) to yield title compound **13b** (623.1 mg, 42 % over two steps). **R_f** (petroleum ether:ethyl acetate 2:1): 0.50; **$UV_{\lambda max}$** (CH_3CN:H_2O): 229, 281, 286, 350 nm; **^{1}H NMR** (600 MHz, $CDCl_3$) δ 8.06 (dd, J = 9.8, 4.8 Hz, 2H, H-4, 8), 7.70 (d, J = 2.9 Hz, 1H, H-2), 7.27 (overlapped, 1H, H-3), 6.74 (ddd, J = 15.2, 9.9, 1.5 Hz, 2H, H-5, 7), 6.53 (s, 1H, H-13), 4.34 – 4.27 (m, 4H, H-18, 20), 3.80 (s, 2H, H-9), 2.64 (s, 3H, H-25), 2.04 (s, 3H, H-24), 1.32 (t, 6H, H-19, 21); **^{13}C NMR** (150 MHz, $CDCl_3$) δ 12.8 (C-25), 14.2 (C-19,21), 23.2 (C-24), 43.3 (C-9), 63.0 (C-18,20), 67.6 (C-10), 117.0 (C-3), 123.3 (C-7), 124.1 (C-5), 126.8 (C-1), 132.5 (C-8), 135.0 (C-4), 135.5 (C-8a), 138.1 (C-2), 139.7 (C-3a), 145.4 (C-6), 167.5 (C-11,12), 169.5 (C-22); **ES-MS (ES+):** calculated for $[M+H]^+$: 372.43, found at $[C_{21}H_{25}NO_5H]^+$: 372.2; **HRMS (ESI):** calculated for $C_{21}H_{25}NO_5^+$ $[M+H]^+$: 372.1805; found: 372.1889.

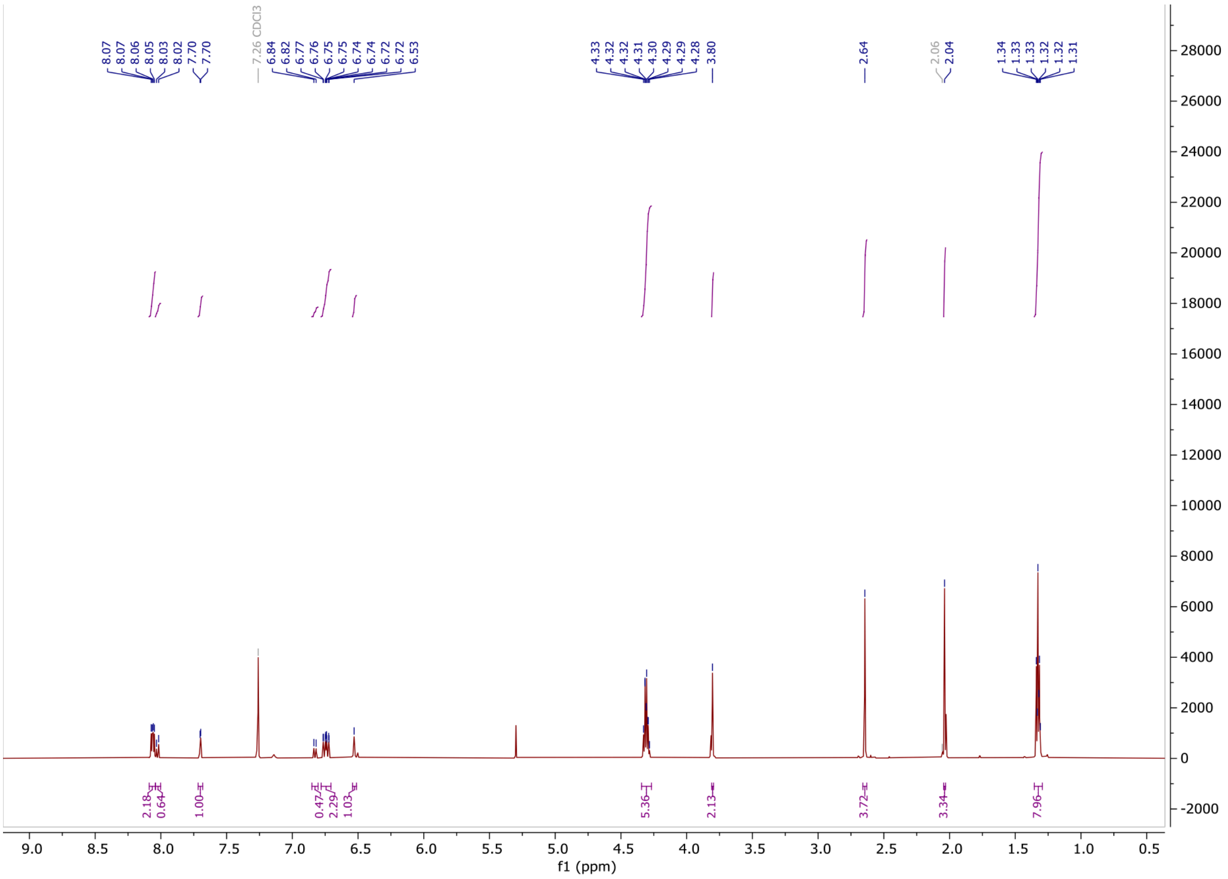

Figure S18. ^{1}H NMR of **13b** ($CDCl_3$).

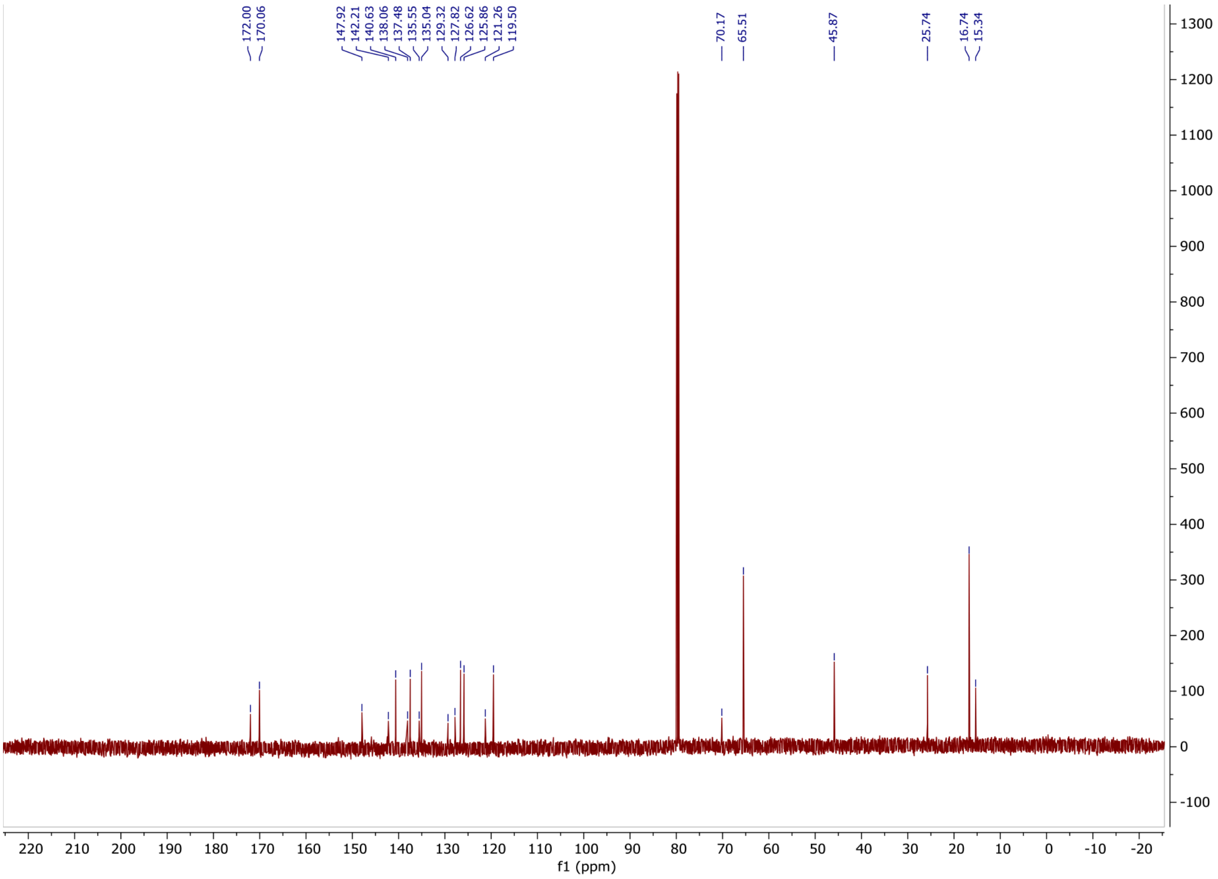

Figure S19. ^{13}C NMR of **13b** ($CDCl_3$).

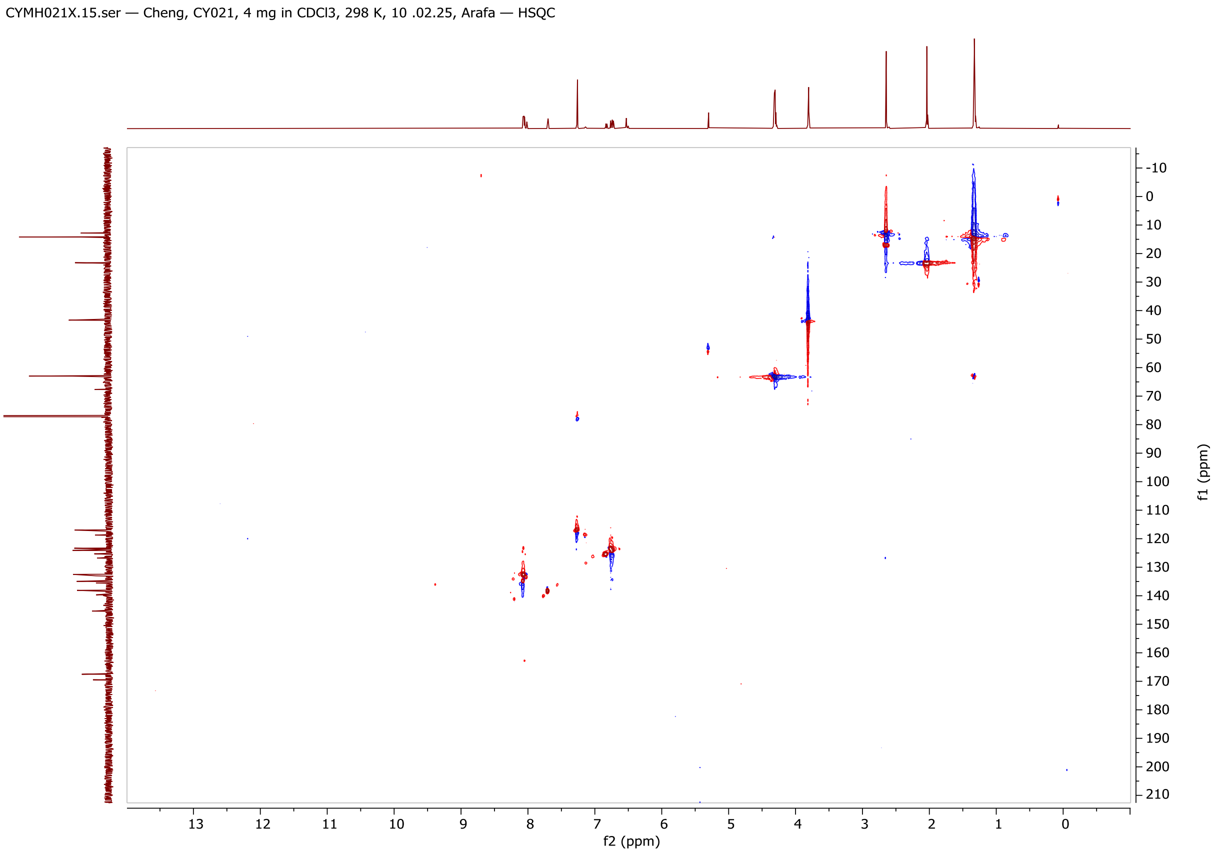

Figure S20. HSQC spectrum of **13b** ($CDCl_3$).

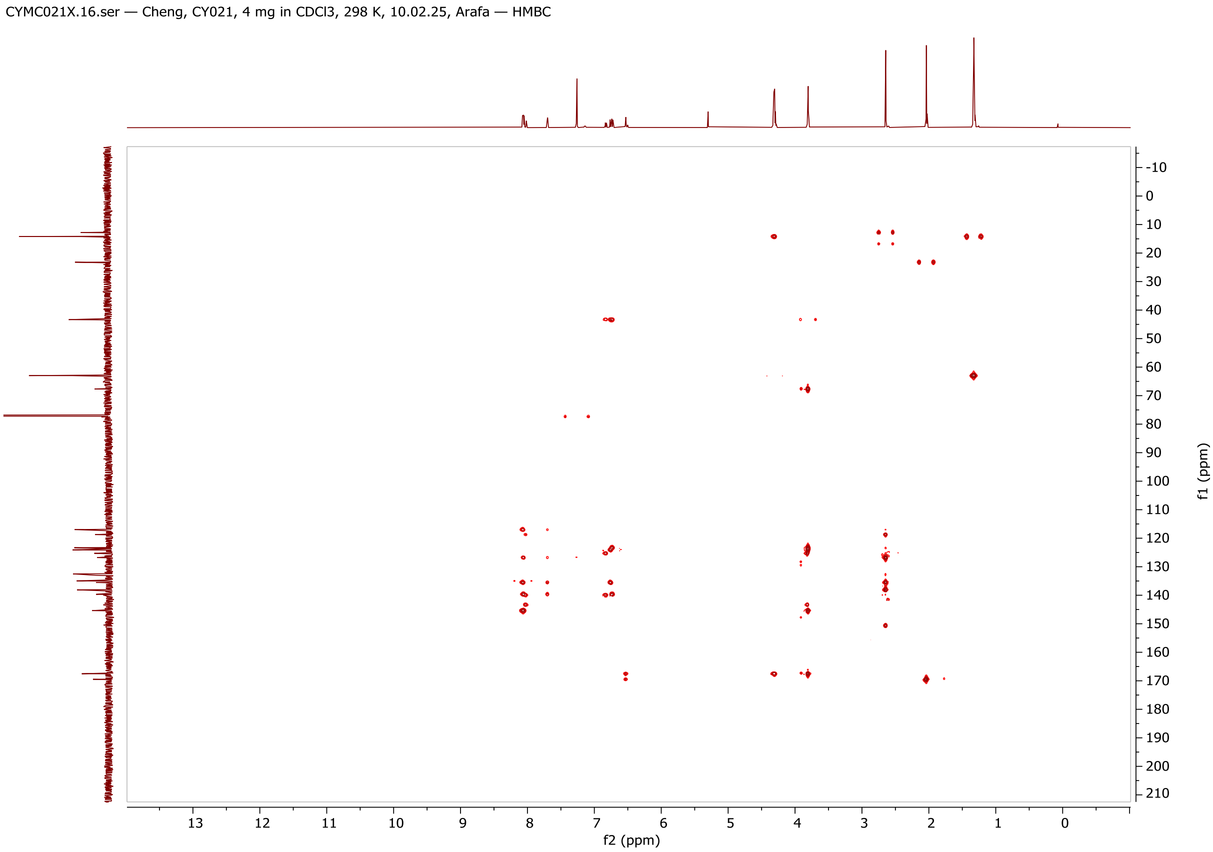

Figure S21. HMBC spectrum of **13b** ($CDCl_3$).

14b *N*-acetyl-β-(6-(1-methyl)azulenyl)alanine

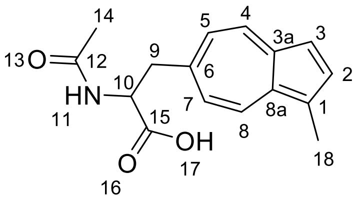

Chemical Formula: $C_{16}H_{17}NO_3$
Exact Mass: 271.1208

To a solution of compound **13b** (200 mg, 0.53 mmol) in THF/water (3.5 mL/1 mL) was added LiOH·H_2O (90 mg, 2.2 mmol) at rt. The resulting purple solution was stirred at 50 °C for 36 h and the THF was removed under reduced pressure. The blue residue was dissolved in water (10 mL) acidified to pH 3, extracted with ethyl acetate (3 × 20 mL) and dried with $MgSO_4$. The solvent was evaporated, and the residue subjected to column chromatography (petroleum ether:ethyl acetate 3:1 → 1:1 with all solvents containing 0.5 % formic acid) to yield title compound **14b** (136 mg, 95 %). **R_f** (petroleum ether:ethyl acetate 1:1 + 1 % FA):0.40; **$UV_{\lambda max}$** (CH_3CN:H_2O): 230, 281, 286, 350 nm; **^{1}H NMR** (600 MHz, MeOD) δ 8.14 (dd, J = 15.5, 9.9 Hz, 2H, H-5,7), 7.65 (d, J = 3.7 Hz, 1H, H-2), 7.23 (d, J = 3.7 Hz, 1H, H-3), 7.07 – 6.98 (m, 2H, H-4, 8), 4.79 (dd, J = 9.4, 5.2 Hz, 1H, H-10), 3.43 – 3.34 (m, 1H, H-9a), 3.13 – 3.05 (m, 1H, H-9b), 2.63 (s, 3H, H-18), 1.90 (s, 3H, H-14); **^{13}C NMR** (150 MHz, MeOD) δ 12.6 (C-18), 22.3 (C-9), 44.3 (C-14), 54.8 (C-10), 117.7 (C-3), 123.7 (C-7), 124.4 (C-5), 127.3 (C-1), 133.5 (C-8), 136.1 (C-4), 136.7 (C-8a), 138.4 (C-2), 141.0 (C-3a), 148.4 (C-6), 173.1 (C-12,15); **ES-MS (ES+):** calculated for $[M+H]^+$: 272.31, found at $[C_{16}H_{17}NO_3]^+$: 272.2; **HRMS (ESI):** calculated for $C_{16}H_{17}NO_3^+$ $[M+H]^+$: 272.1281; found: 272.1343.

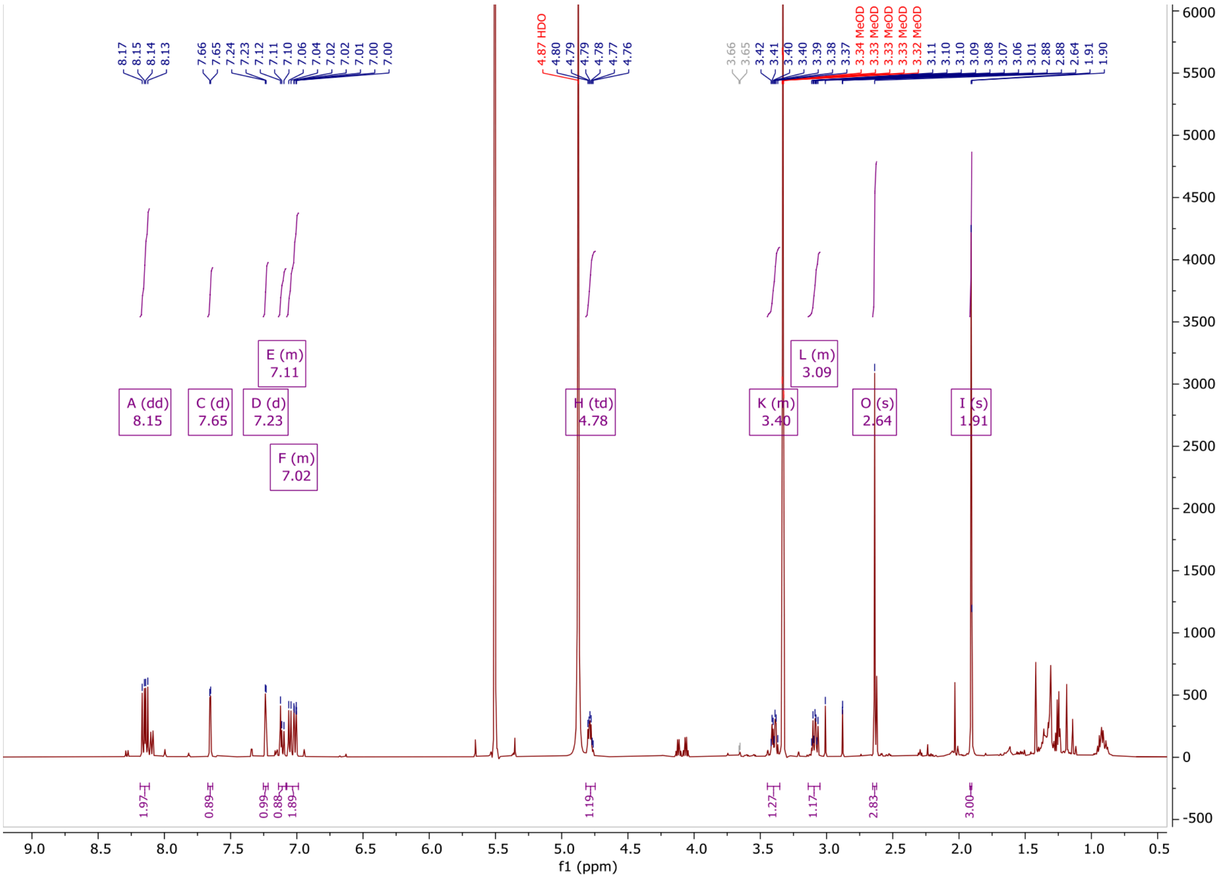

Figure S22. ^{1}H NMR of **14b** (methanol-d$_4$).

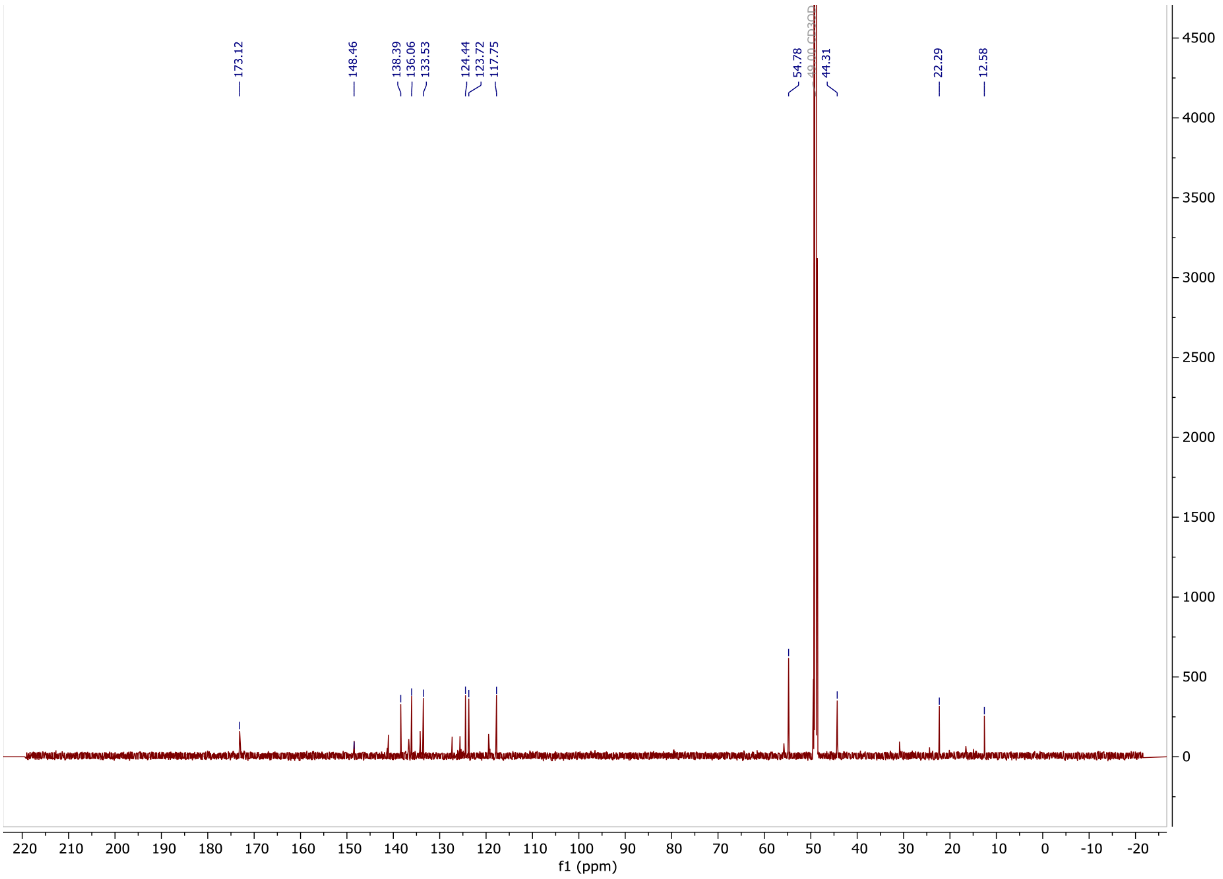

Figure S23. ^{13}C NMR of **14b** (methanol-d$_4$).

Determination of Enantiomeric Excess

The procedure was followed as described by *P. Marfey*,[4] except the amino acids were dissolved in DMSO. 50 µL of the racemic (15 mM) or *L*-β-(6-azulenyl)alanine (50 mM) were placed in separate 1.5 mL tubes. To each solution was added 100 µL of a 1% acetone solution of 1-fluoro-2-4-dinitrophenyl-5-*L*-alanine amide (FDAA, Marfey's reagent) and 20 µL of aqueous 1M $NaHCO_3$. The resulting solution was heated to 40 °C for 1 h under constant stirring. The reaction mixtures were allowed to cool to room temperature before 10 µL of 2 M HCl was added. The solvent was removed, and the residue dissolved in DMSO (500 µL). 50 µL of this solution was dissolved in 150 µL acetonitrile, filtered and submitted to HPLC analysis. The analysis was done at 281 nm close to the λ_{max} for both diasteromers.

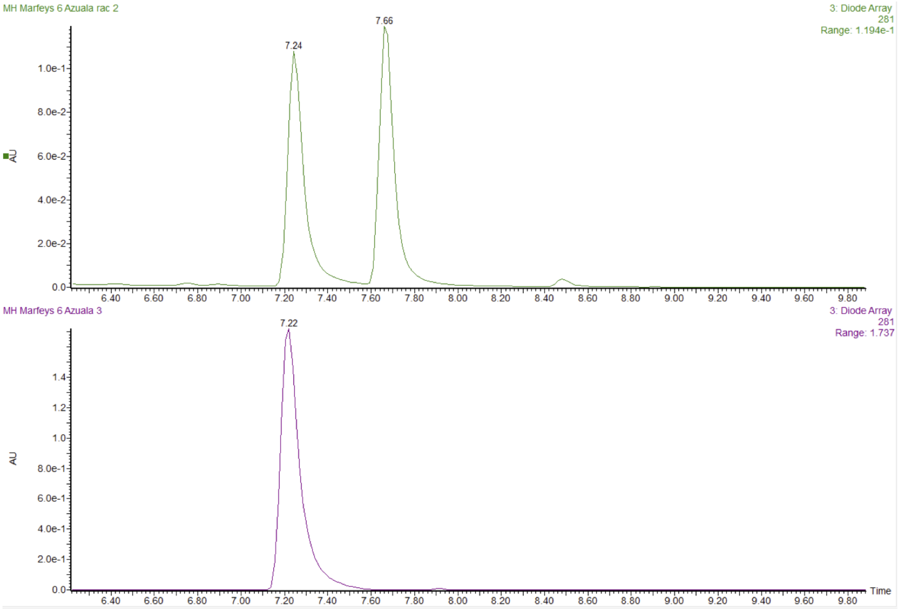

Figure S24. HPLC- analysis of derivatisation products of racemic D/L-β-(6-azulenyl)alanine (Top) and L-β-(6-azulenyl)alanine (Bottom) with Marfey's reagent. Detection at 281 nm.

2.1 Feeding Experiments and Isolation of Mutasynthetic Compounds.

General fermentation and feeding protocols

P. grisea ΔpyiA[5] spores were plated from a stock solution onto DPY agar plates and allowed to grow for 7 days at 28 °C. After sufficient growth, spores and mycelia were collected with double distilled sterile water and used for the inoculation of liquid DPY media. The amino acids were dissolved in double distilled water (final concentration of 10 mg/ml) and filter sterilized. The acetylated derivatives were dissolved in aqueous 0.1M NaOH made up in double distilled water (final concentration: 10 mg / mL) and filter sterilized. Generally, the *P. grisea ΔpyiA* knock-out strains collected from one plate (Ø 60 mm) were inoculated into 10 × 500 mL Erlenmeyer flasks containing 100 mL of DPY media and each flask directly supplemented with either the L-amino acid (200 µL) or acetylated racemic amino acid (400 µL). The feeding was repeated three times in 24h intervals. 7 days after inoculation the cultures were vacuum filtered through a Buchner funnel equipped with a paper filter to remove the mycelium from the fermentation broth. The aqueous liquid was then transferred into a separating funnel and extracted twice with equal volumes of ethyl acetate. The combined organic layers were dried over $MgSO_4$, filtered through folded filter papers, and evaporated to dryness. The residue was dissolved in HPLC grade MeOH to a concentration of 10 mg/mL, filtered through cotton, and submitted to analytical HPLC or 20 mg/mL and purified by mass-directed HPLC.

In parallel to each experimental feeding experiment, a positive control was run using the native substrate *O*-Methyl-L-tyrosine, and a negative control with the supplementation of water was run. In the positive control, pyrichalasin H **1a** itself is produced (see Figure S2 and S4). In the negative control incorporation of endogenous L-phenylalanine occurs and cytochalasin H **1b** is observed at low level (see Figure S2 and S3).

Feeding of *N*-acetyl-*β*-(6-azulenyl)alanine 14a to *P. grisea ΔpyiA*

200 mg of *N*-acetyl-β-(6-azuleno)alanine **14a** was dissolved in 0.1 M aqueous NaOH made up in double distilled water (final concentration: 10 mg / mL) and filter sterilized. 400 µl of this solution was fed to *P. grisea ΔpyiA*[5,6] inoculated into 20 × 500 mL baffled or non-baffled Erlenmeyer flasks containing 100 mL sterile DPY liquid media on shakers at 110 rpm at 25 °C. The feeding was repeated at 24 h intervals three times to a final concentration of 0.6 mM. On the 7th day the cultures were vacuum filtered through a Buchner funnel equipped with a paper filter to remove the mycelium from the fermentation broth. The aqueous liquid was transferred into a separating funnel and extracted twice with equal volumes of ethyl acetate. The combined organic layers were dried over $MgSO_4$, gravity filtered through folded filter papers, and evaporated to dryness. The residue was dissolved in HPLC grade MeOH to a concentration of 20 mg/ml, filtered through cotton, and purified by mass-directed HPLC.

Feeding of L-tryptophan, L-β-(6-azulenyl)alanine 6a, L-β-(1-azulenyl)alanine 5 and *N*-acetyl-β-(6-azulenyl)alanine 14b to *P. grisea ΔpyiA*

10 mg of L-tryptophan (purchased from Carl Roth) was dissolved in double distilled water (final concentration: 10 mg/ml). 200 µl of this solution was directly fed to *P. grisea ΔpyiA* inoculated into 2 × 500 mL baffled Erlenmeyer flasks containing 100 mL sterile DPY liquid media on shakers at 110 rpm at 25 °C. The feeding was repeated at 24 h intervals three times and a final concentration 0.39 mM. On the 7th day the cultures were extracted individually using the method described above. The residue was dissolved in HPLC grade MeOH to a concentration of 10 mg/ml, filtered through cotton, and submitted to analytical HPLC.

200 mg of *N*-acetyl-β-(6-(1-methyl)azulenyl)alanine **14b** was dissolved in 0.1 M aqueous NaOH made up in double distilled water (final concentration: 10 mg / mL) and filter sterilized. 400 µl of this solution was directly fed to *P. grisea ΔpyiA* inoculated into 2 × 500 mL baffled Erlenmeyer flasks containing 100 mL sterile DPY liquid media on shakers at 110 rpm at 25 °C. The feeding was repeated at 24 h intervals three times to a final concentration of 0.6 mM. On the 7th day the cultures were extracted individually using the method described above. The residue was dissolved in HPLC grade MeOH to a concentration of 10 mg/ml, filtered through cotton, and submitted to analytical HPLC.

20 mg of L-β-(6-azulenyl)alanine **6a** was dissolved in double distilled water (ultra sonication needed, final concentration: 5 mg/mL) and filter sterilized. 200 µl of this solution was directly fed to *P. grisea ΔpyiA* inoculated into 2 × 250 mL non-baffled Erlenmeyer flasks each containing 50 mL sterile DPY liquid media on shakers at 110 rpm at 25 °C. The feeding was repeated at 24 h intervals three times and to a final concentration of 0.37 mM. On the 7th day the cultures were extracted individually using the method described above. The residue was dissolved in HPLC grade MeOH to a concentration of 10 mg/ml, filtered through glass wool, and submitted to analytical HPLC.

40 mg of L-β-(1-azulenyl)alanine **5** was dissolved in double distilled water (ultra sonication needed, final concentration: 5 mg /mL) and filter sterilized. 200 µl of this solution was directly fed to *P. grisea ΔpyiA* inoculated into 2 × 250 mL non-baffled Erlenmeyer flasks each containing 50 mL sterile DPY liquid media on shakers at 110 rpm at 25

°C. The feeding was repeated at 24 h intervals three times to a final concentration of 0.37 mM. On the 7th day the cultures were extracted individually using the method described above. The residue was dissolved in HPLC grade MeOH to a concentration of 10 mg/ml, filtered through cotton, and submitted to analytical HPLC.

Feeding of L-β-(1-azulenyl)alanine 5 and L-β-(6-azulenyl)alanine 6a to *Chaetomium globosum*.
C. globosum DSM-1962, was obtained from the DSMZ Leibniz institute (Braunschweig, Germany) and cultivated according to the methods of Tan and coworkers.[7] L-β-(1-azulenyl)alanine **5** was prepared and purified according to Kalesse and coworkers.[8] The amino acid was dissolved in double distilled water (ultra sonication needed, final concentration: 5 - 10 mg / mL) and filter sterilized. The strain was allowed to grow on PB-Agar for 10-14 days at 27 °C until sufficient sporulation was achieved. The spores were collected using double distilled water and introduced into liquid CD media enriched with 0.1% (*w/v*) yeast extract. The inoculated media was then divided into 250 mL non-baffled Erlenmeyer flasks, to a final volume of 50 mL of inoculated liquid media. The cultures were allowed to grow for 24 h at 160 rpm and 28 °C before the addition of L-β-(1-azulenyl)alanine **5** to a final concentration of 2 mM. Following the addition, the cultures were allowed to grow for an additional 4 days under the same conditions. Afterwards, the cultures were filtered through a Buchner funnel equipped with a paper filter to remove the mycelium from the fermentation broth, and the fermentation broth was transferred into a separating funnel. The aqueous phase was extracted twice with equal volumes of ethyl acetate. The combined organic layers were dried over $MgSO_4$, filtered through a folded filter paper, and evaporated to dryness. The crude residue was dissolved in HPLC grade MeOH to a concentration of 20 mg/ml and submitted to analytical LCMS.

2.2 Feeding Experiments LCMS data

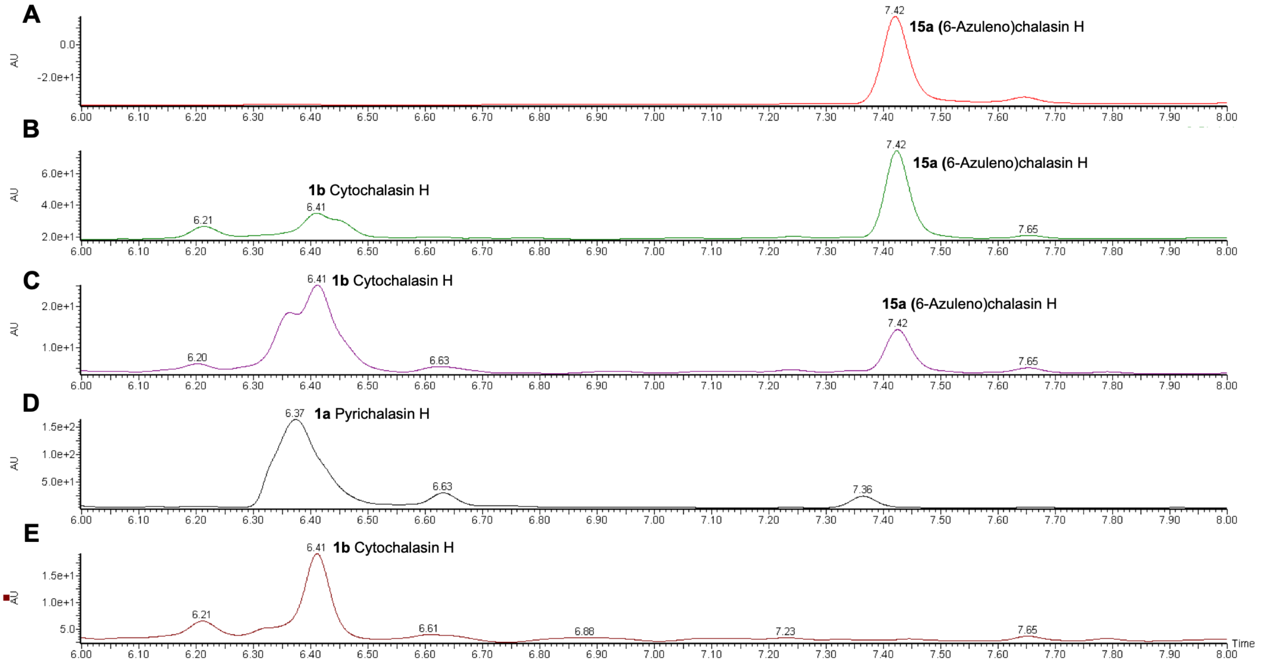

Figure S25. LCMS analysis of feeding experiments to *P. grisea ΔpyiA*. **A**, Purified **15a** (DAD, 200-600 nm); **B**, Extract of *P. grisea ΔpyiA* supplemented with **14a** (DAD, 200-600 nm); **C**, Extract of *P. grisea ΔpyiA* supplemented with **6a** (DAD, 200-600 nm); **D,** Extract of *P. grisea ΔpyiA* supplemented with **2b** (DAD, 200-600 nm); **E**, Extract of *P. grisea ΔpyiA* supplemented with H_2O (DAD, 200-600 nm).

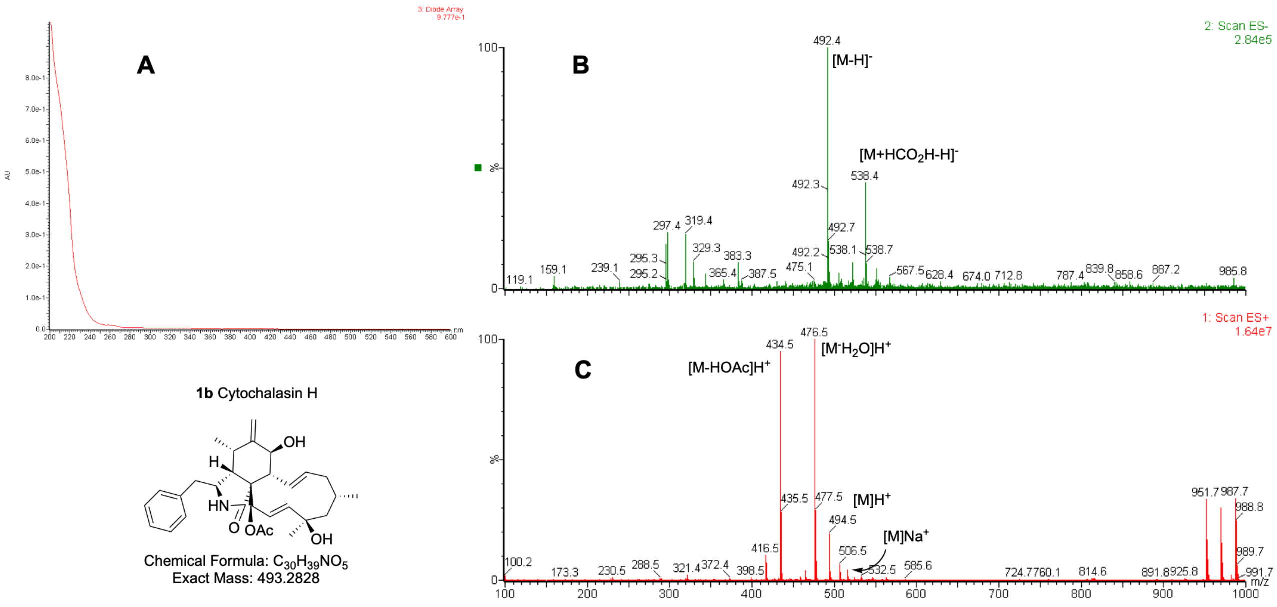

Figure S26. UV and MS data for Cytochalasin H **1b**, RT 6.41 min. **A**, UV absorbtion; **B**, ES- ionisation; **C**, ES+ ionisation.

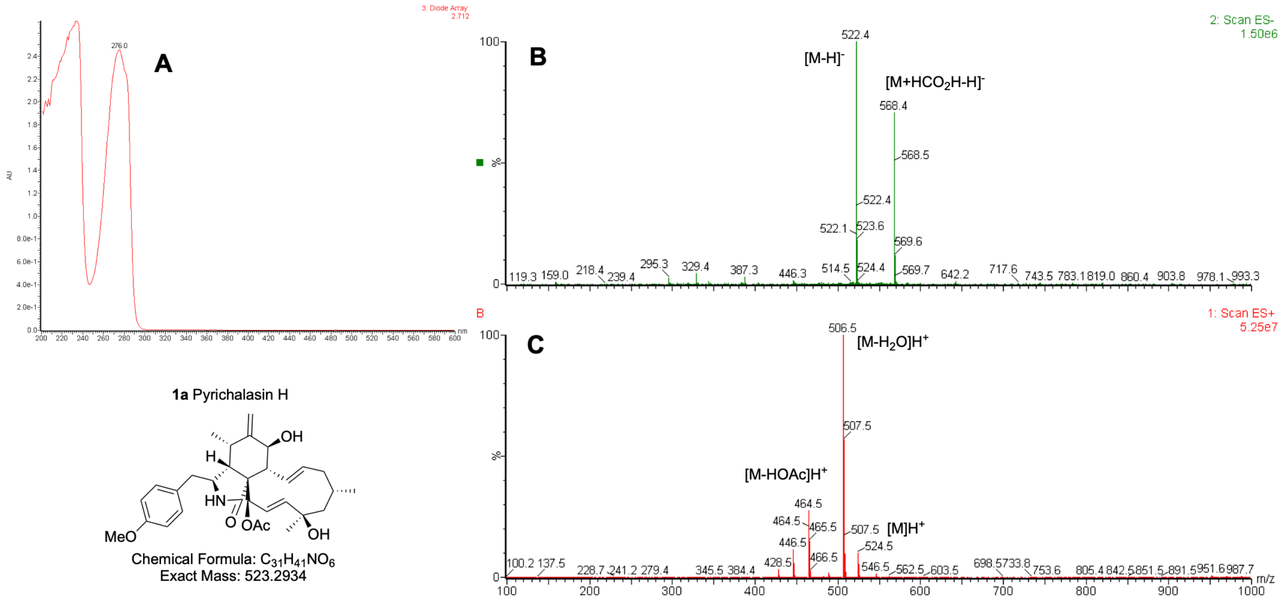

Figure S27. UV and MS data for Pyrichalasin H **1a**, RT 7.35 min. **A**, UV absorbtion; **B**, ES- ionisation; **C**, ES+ ionisation.

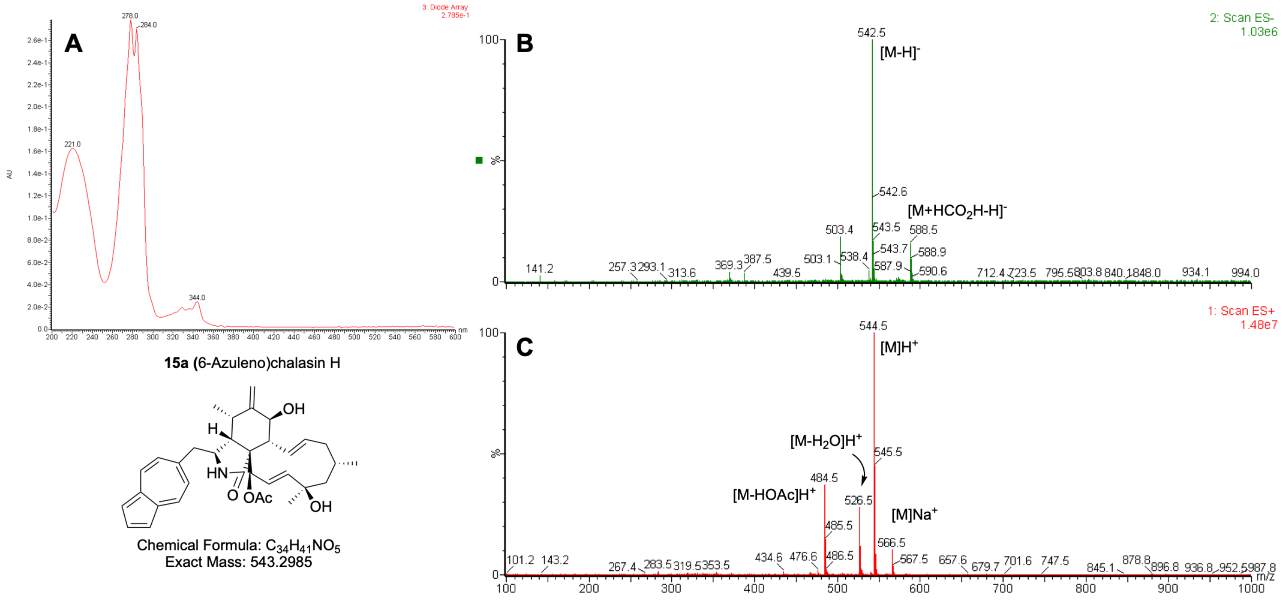

Figure S28. UV and MS data for (6-azuleno)chalasin H **15a**, RT 7.42 min. **A**, UV absorbtion; **B**, ES- ionisation; **C**, ES+ ionisation.

Single Mass Analysis

Tolerance = 50.0 PPM / DBE: min = -1.5, max = 50.0
Element prediction: Off
Number of isotope peaks used for i-FIT = 3

Monoisotopic Mass, Even Electron Ions
78 formula(e) evaluated with 7 results within limits (all results (up to 1000) for each mass)
Elements Used:
C: 0-40 H: 0-50 N: 0-1 O: 0-5 Na: 0-1

Hauser QTof Premier HAB321
MU 229 289 (2.967) AM (Cen,4, 70.00, Ht,10000.0,556.28,0.70,LS 10)

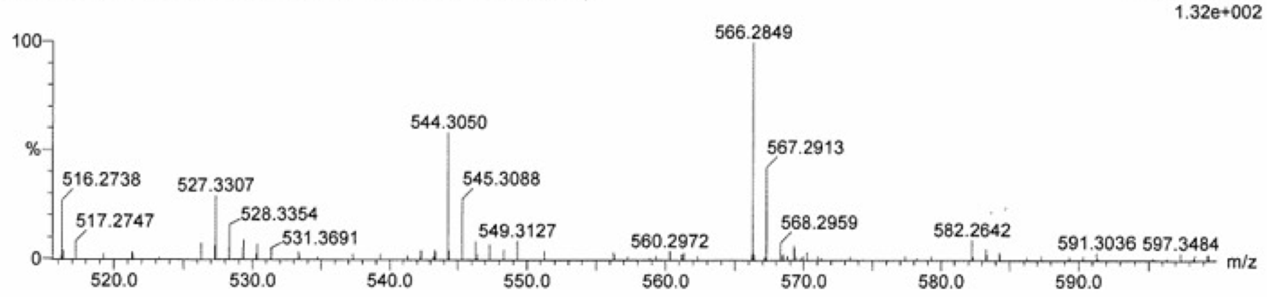

Mass	Calc. Mass	mDa	PPM	DBE	i-FIT	i-FIT (Norm)	Formula
Minimum:				-1.5			
Maximum:		5.0	50.0	50.0			
544.3050	544.3039	1.1	2.0	11.5	13.6	1.7	C32 H43 N O5 Na
	544.3063	-1.3	-2.4	14.5	13.2	1.3	C34 H42 N O5
	544.2980	7.0	12.9	20.5	13.2	1.3	C39 H39 N Na
	544.3191	-14.1	-25.9	15.5	14.1	2.2	C36 H43 N O2 Na
	544.3216	-16.6	-30.5	18.5	14.2	2.3	C38 H42 N O2
	544.2852	19.8	36.4	19.5	15.1	3.1	C37 H38 N O3
	544.2828	22.2	40.8	16.5	15.7	3.8	C35 H39 N O3 Na

Figure S29. HRMS analysis for (6-azuleno)chalasin H **15a**.

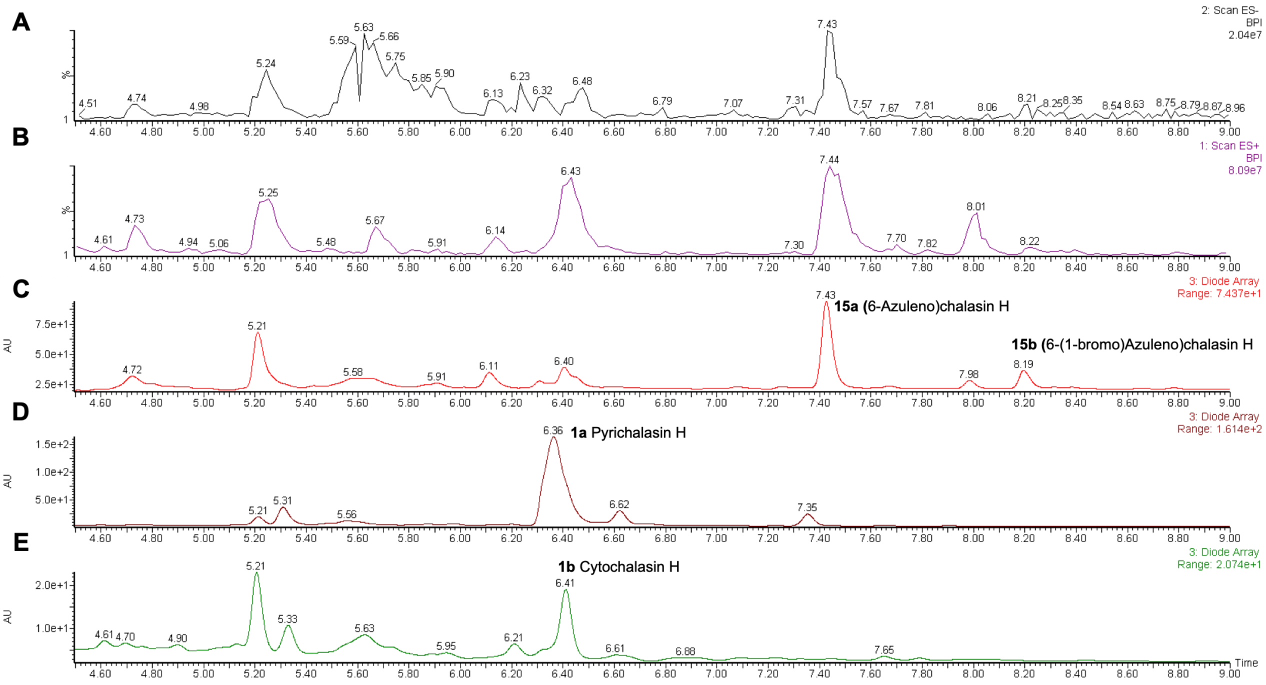

Figure S30. LCMS analysis of feeding experiments to *P. grisea ΔpyiA*. **A**, ES- total ion current for chromatogram C; **B**, ES+ total ion current for chromatogram C; **C**, Extract of *P. grisea ΔpyiA* supplemented with **6a** using baffled flasks (DAD, 200-600 nm); **D,** Extract of *P. grisea ΔpyiA* supplemented with **2b** (DAD, 200-600 nm); **E**, Extract of *P. grisea ΔpyiA* supplemented with H_2O (DAD, 200-600 nm).

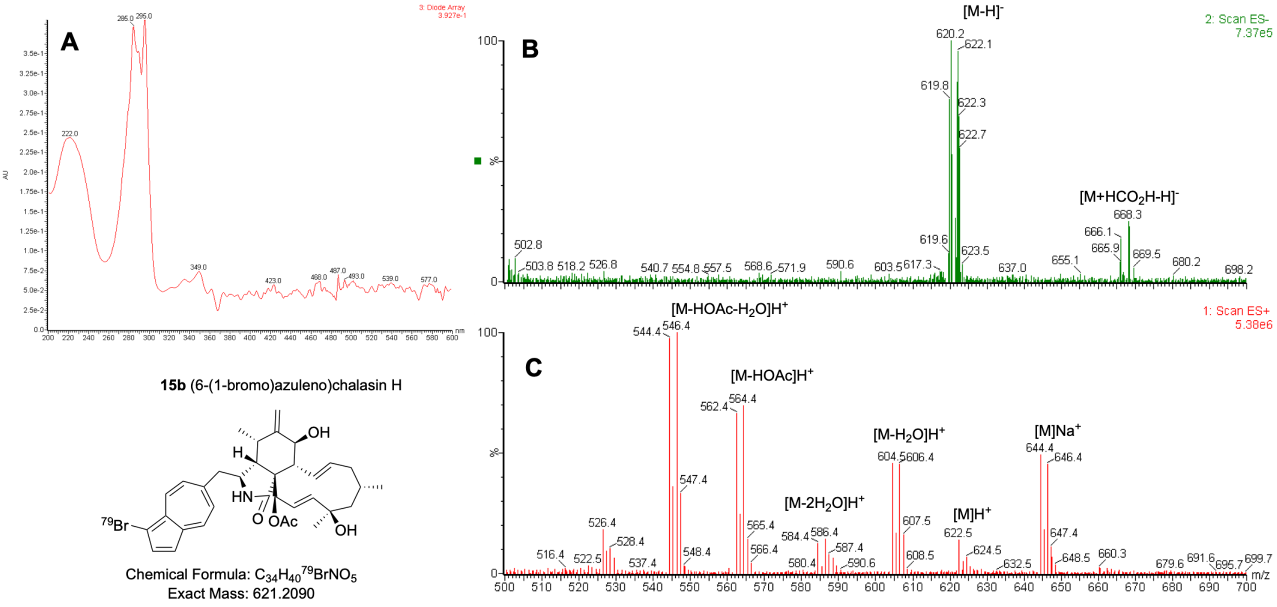

Figure S31. UV and MS data for (6-(1-bromo)azuleno)chalasin H **15b**, RT 8.19 min. **A**, UV absorbtion; **B**, ES- ionisation; **C**, ES+ ionisation.

Single Mass Analysis
Tolerance = 20.0 PPM / DBE: min = -1.5, max = 50.0
Element prediction: Off
Number of isotope peaks used for i-FIT = 3

Monoisotopic Mass, Even Electron Ions
344 formula(e) evaluated with 6 results within limits (all results (up to 1000) for each mass)
Elements Used:
C: 0-70 H: 0-100 N: 0-1 O: 0-5 Na: 0-1 Br: 0-1

Hauser QTof Premier HAB321
MU 246 360 (3.403) AM (Cen,4, 70.00, Ht,10000.0,554.26,0.70,LS 10) 1: TOF MS ES- 1.20e+002

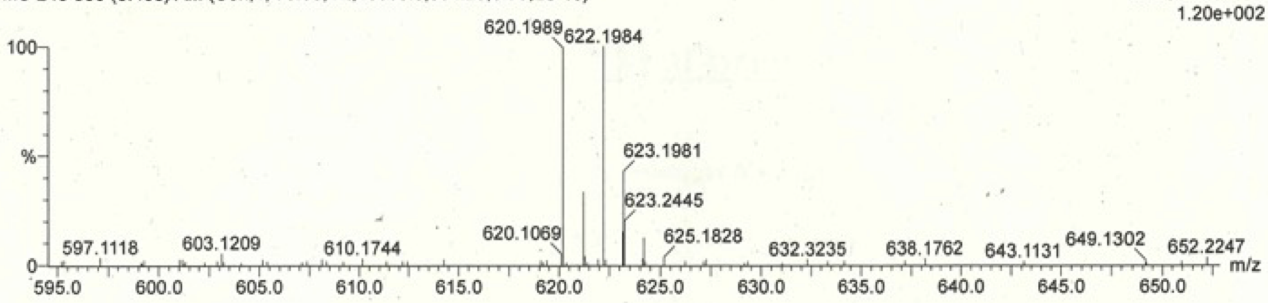

Mass	Calc. Mass	mDa	PPM	DBE	i-FIT	i-FIT (Norm)	Formula
Minimum:				-1.5			
Maximum:		5.0	20.0	50.0			
620.1989	620.1990	-0.1	-0.2	32.5	27.7	9.9	C45 H27 N O Na
	620.1988	0.1	0.2	12.5	18.6	0.8	C32 H40 N O5 Na Br
	620.2012	-2.3	-3.7	15.5	18.8	1.0	C34 H39 N O5 Br
	620.2014	-2.5	-4.0	35.5	28.0	10.2	C47 H26 N O
	620.1953	3.6	5.8	24.5	20.1	2.3	C41 H35 N Br
	620.1929	6.0	9.7	21.5	20.4	2.6	C39 H36 N Na Br

Figure S32. HRMS analysis for (6-(1-bromo)azuleno)chalasin H **15b**.

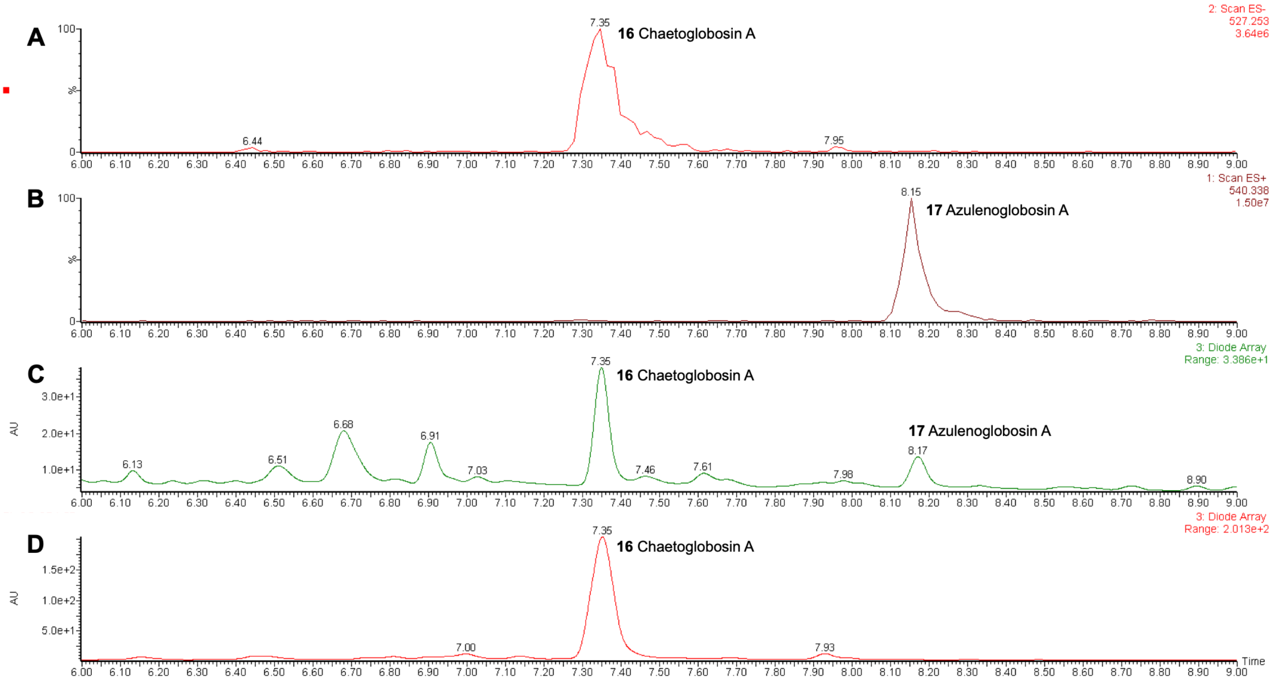

Figure S33. Feeding experiments with *Chaetomium globosum*. **A**, Extracted ion chromatogram ES- *m/z* 527.3 for *C. globosum* supplemented with L-β-(1-azulenyl)alanine **5**; **B**, Extracted ion chromatogram ES- *m/z* 540.34 for *C. globosum* supplemented with L-β-(1-azulenyl)alanine **5**; **C**, Extract of *C. globosum* supplemented with L-β-(1-azulenyl)alanine **5** (DAD, 200-600 nm); **D**, Extract of *C. globosum* supplemented with H_2O (DAD, 200-600 nm).

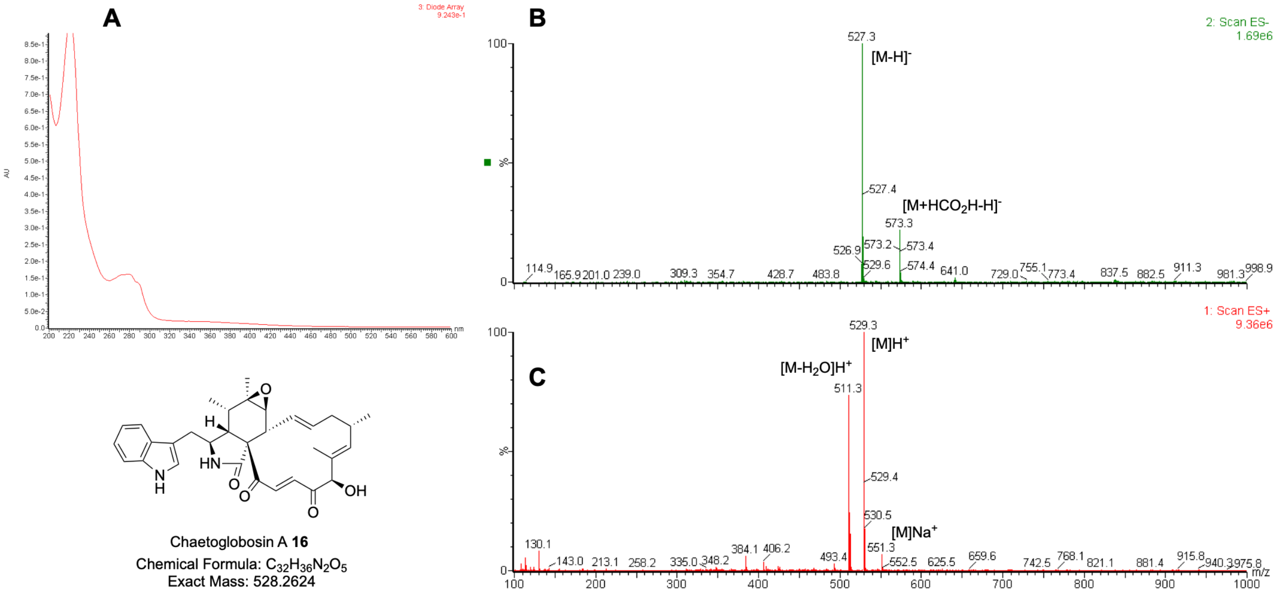

Figure S34. UV and MS data for Chaetoglobosin A **16**, RT 7.35 min. **A**, UV absorbtion; **B**, ES- ionisation; **C**, ES+ ionisation.

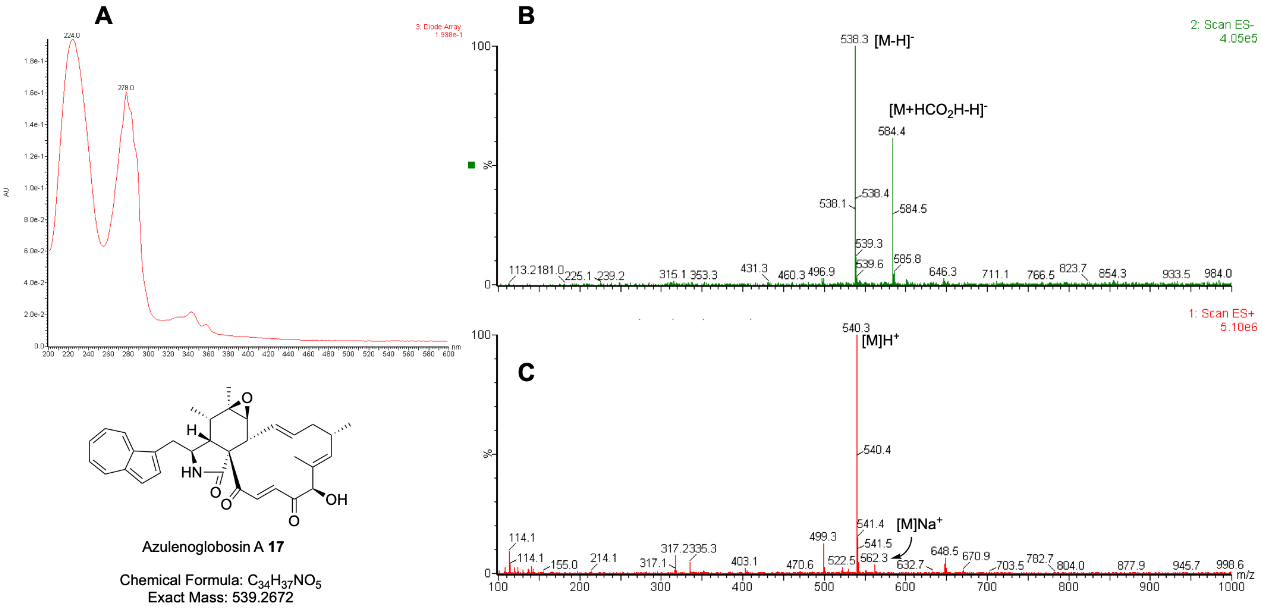

Figure S35. UV and MS data for Azulenoglobosin A **17**, RT 8.17 min. **A**, UV absorbtion; **B**, ES- ionisation; **C**, ES+ ionisation.

Single Mass Analysis

Tolerance = 50.0 PPM / DBE: min = -1.5, max = 50.0
Element prediction: Off
Number of isotope peaks used for i-FIT = 3

Monoisotopic Mass, Even Electron Ions
168 formula(e) evaluated with 8 results within limits (all results (up to 1000) for each mass)
Elements Used:
C: 0-75 H: 0-160 N: 0-1 O: 0-5 Na: 0-1

Hauser QTof Premier HAB321
MU 262 349 (3.580) AM (Cen,4, 95.00, Ht,10000.0,556.28,0.70,LS 5) 1: TOF MS ES+ 2.00e+002

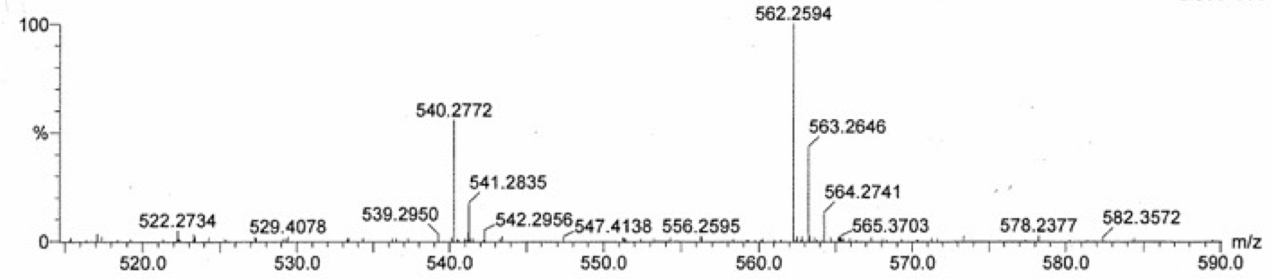

Minimum:				-1.5			
Maximum:		5.0	50.0	50.0			

Mass	Calc. Mass	mDa	PPM	DBE	i-FIT	i-FIT (Norm)	Formula
562.2594	562.2593	0.1	0.2	19.5	31.0	1.5	C36 H36 N O5
	562.2569	2.5	4.4	16.5	31.5	2.0	C34 H37 N O5 Na
	562.2535	5.9	10.5	28.5	31.0	1.5	C43 H32 N
	562.2511	8.3	14.8	25.5	31.2	1.8	C41 H33 N Na
	562.2722	-12.8	-22.8	20.5	31.6	2.2	C38 H37 N O2 Na
	562.2746	-15.2	-27.0	23.5	31.7	2.2	C40 H36 N O2
	562.2382	21.2	37.7	24.5	32.9	3.4	C39 H32 N O3
	562.2358	23.6	42.0	21.5	33.5	4.0	C37 H33 N O3 Na

Figure S36. HRMS analysis for Azulenoglobosin A **17**.

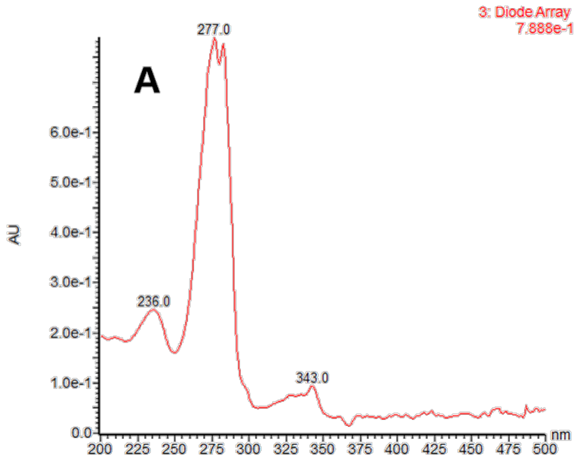

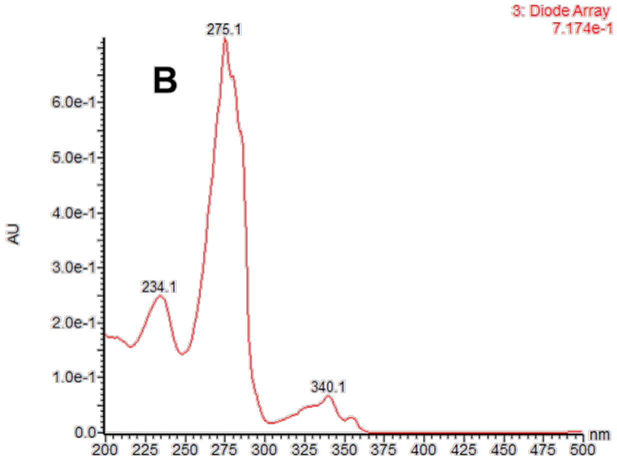

6a L-β-(6-azulenyl)alanine

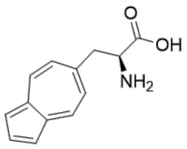

5 L-β-(1-azulenyl)alanine

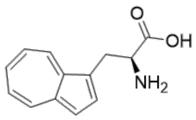

Figure S37. UV data for: **A**, L-β-(6-azulenyl)alanine **6a**; **B**, L-β-(6-azulenyl)alanine **5.**

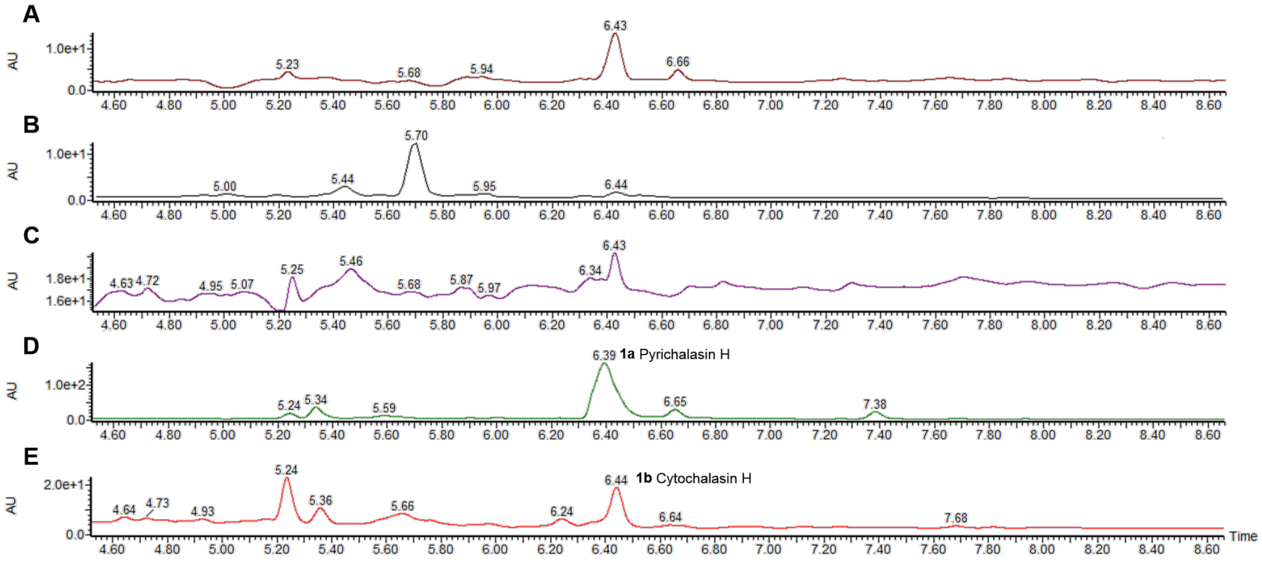

Figure S38. LCMS analysis of feeding experiments to *P. grisea ΔpyiA*. **A**, Extract of *P. grisea ΔpyiA* supplemented with **14b** (DAD, 200-600 nm); **B**, Extract of *P. grisea ΔpyiA* supplemented with **5** (DAD, 200-600 nm); **C**, Extract of *P. grisea ΔpyiA* supplemented with L-Tryptophan using baffled flasks (DAD, 200-600 nm); **D,** Extract of *P. grisea ΔpyiA* supplemented with **2b** (DAD, 200-600 nm); **E**, Extract of *P. grisea ΔpyiA* supplemented with H_2O (DAD, 200-600 nm)**.**

2.3 NMR Characterization of new Natural Products

15a (6-azuleno)chalasin H.

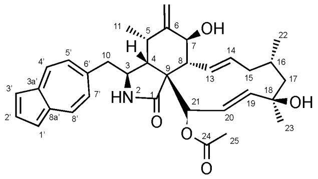

Chemical Formula: $C_{34}H_{41}NO_5$
Exact Mass: 543.2985

UV$_{\lambda max}$ (MeOH): 235, 278, 284, 344 nm; **HRMS (ESI):** calculated for $C_{34}H_{42}NO_5$ $[M+H]^+$: 544.3063; found: 544.3050

Position	δ_H	M	J_{H-H}/ Hz	δ_C	HSQC	HMBC H to C	H-H COSY
**1**	-	-	-	176.8	-	-	-
**2**	5.49	brs	-	-	-	3, 4, 9	-
**3**	3.36	ddd	10, 4.5, 3.8	56.9	CH	-	4, 10a, 10b
**4**	2.20	dd	3.8, 7.4	53.3	CH	-	5, 3
**5**	2.84	m	-	35.5	CH	-	4, 11
**6**	-	-	-	150.3	-	-	-
**7**	3.84	d	10.5	72.4	CH	-	8, 12a, 12b
**8**	2.95	dd	10.5, 9.8	49.9	CH	1, 7, 9, 13, 14	7, 13
**9**	-	-	-	54.4	-	-	-
**10a**	2.84	dd	10.0, 13.0	54.3	CH_2	3, 5'	3, 10b
**10b**	3.05	dd	13.0, 4.5			5'	3, 10a
**11**	1.06	d	6.6	16.6	CH_3	4, 5, 6	5
**12a**	5.14	brs	-	117.1	CH_2	5, 7	5, 7, 12b
**12b**	5.36	brs	-			5, 7	5, 7, 12a
**13**	5.75	dd	15.6, 9.8	129.7	CH	8, 15	8, 14
**14**	5.42	ddd	15.4, 10.3, 5.1	141.7	CH	-	13, 15a, 15b
**15a**	1.79	m	-	45.4	CH_2	-	13, 16, 15b
**15b**	2.03	m	-			-	13, 15, 15a
**16**	1.83	m	-	31.1	CH	-	22
**17a**	1.62	dd	-	56.4	CH_2	18, 19, 22	16, 17b
**17b**	1.92	dd	3.5, 14.5-			-	16, 17a
**18**	-	-	-	77.0	-	-	-
**19**	5.57	dd	16.6, 2.3	140.9	CH	21	20, 21
**20**	5.89	dd	16.6, 2.6	128.5	CH	18, 21	19, 21
**21**	5.65	dd	2.5, 2.0	80.0	CH	4, 8, 9, 19, 20, 24	20, 19
**22**	1.07	d	6.5	29.1	CH_3	15, 16, 17	16
**23**	1.37	s	-	33.8	CH_3	17, 18, 19	-
**24**	-	-	-	173.0	-	-	-
**25**	2.27	s	-	23.6	CH_3	24	-
**6’**	-	-	-	149.9	-	-	-
**5’/7’**	6.99	d	9.9	126.5	2 × CH	10, 4’/8’	4'/8'
**4’/8’**	8.27	d	9.8	138.6	2 × CH	3'/1', 3a'/8a', 6'	5’/7’
**3a’/8a’**	-	-	-	141.7	-	-	-
**3’/1’**	7.39	d	3.7	121.5	2 × CH	6’, 4’/8’	2'
**2’**	7.88	t	3.8	139.6	CH	4’/8’	3'/1'

Table S 1: Summarized NMR signals and 2D-correlations for azulenochalasin H **15a** recorded in $CDCl_3$ at 600 MHz/ 150 MHz.

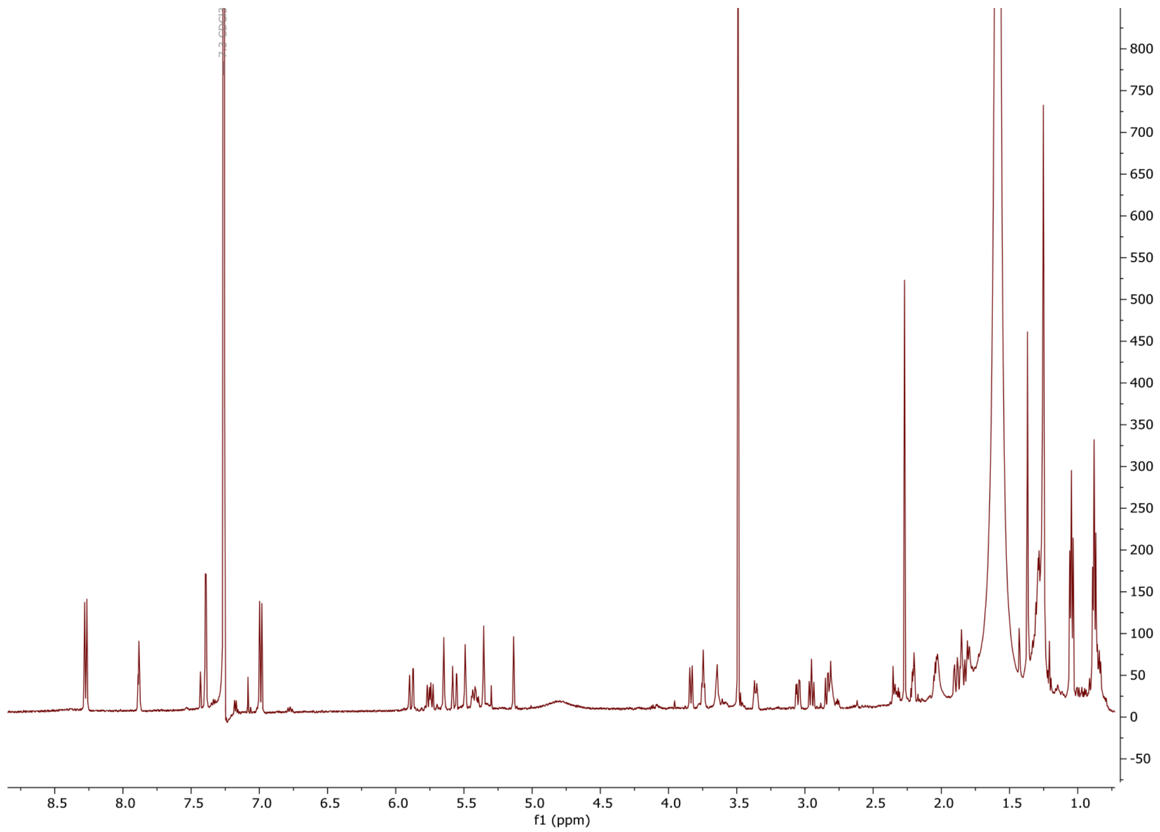

Figure S39. ^{1}H NMR of azulenochalasin H **15a.**

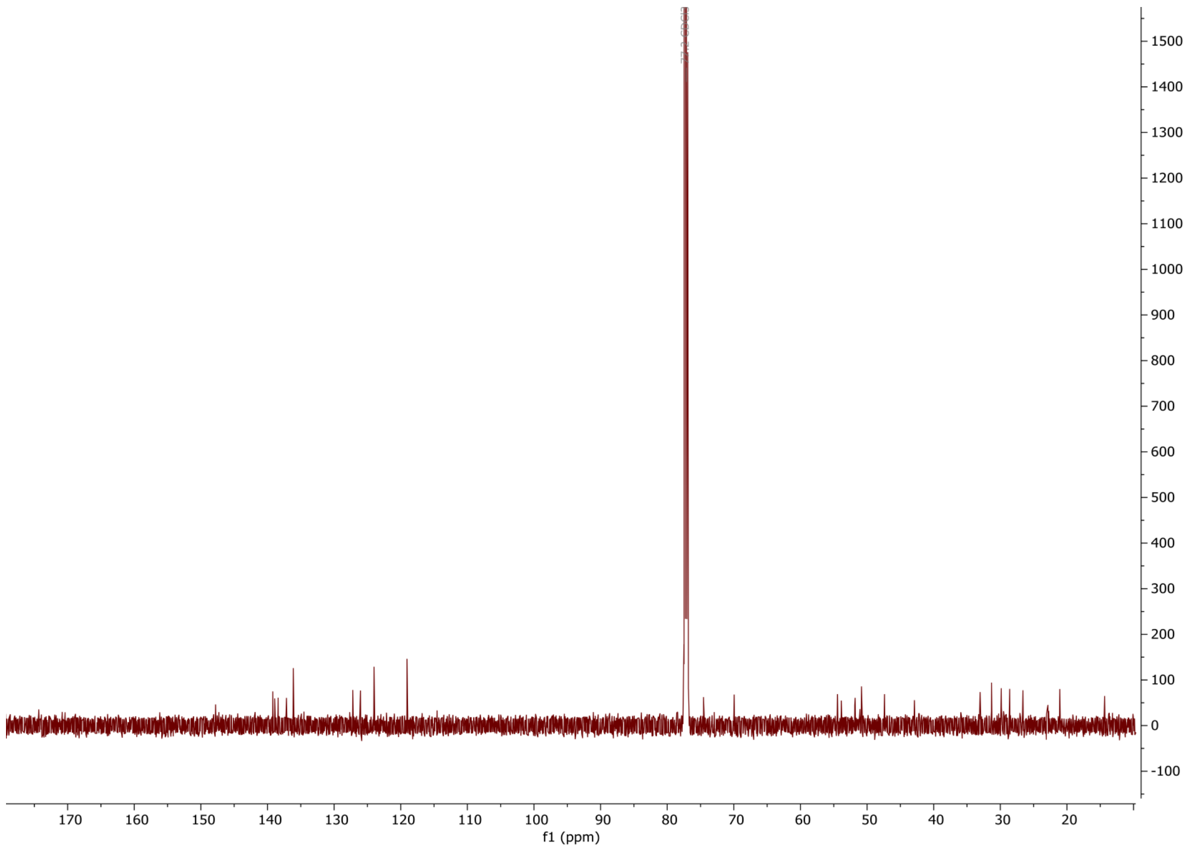

Figure S40. ^{13}C NMR of azulenochalasin H **15a.**

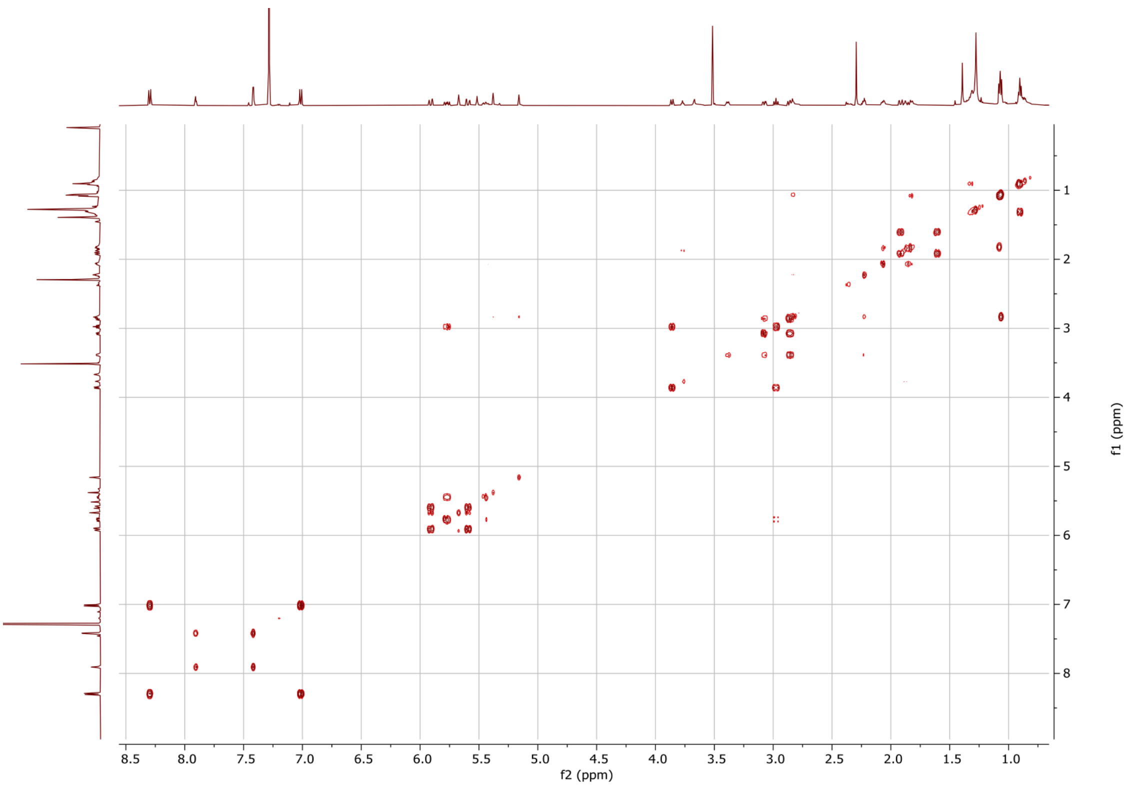

Figure S41. COSY NMR of azulenochalasin H **15a.**

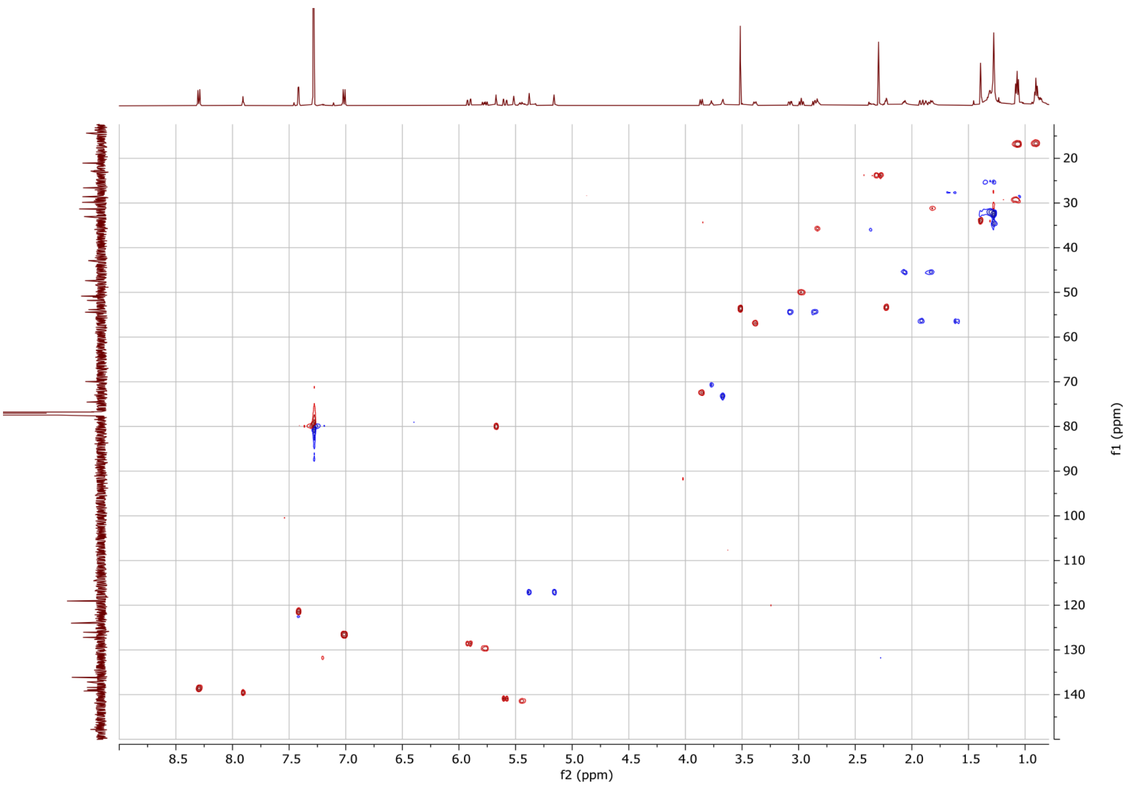

Figure S42. HSQC NMR of azulenochalasin H **15a.**

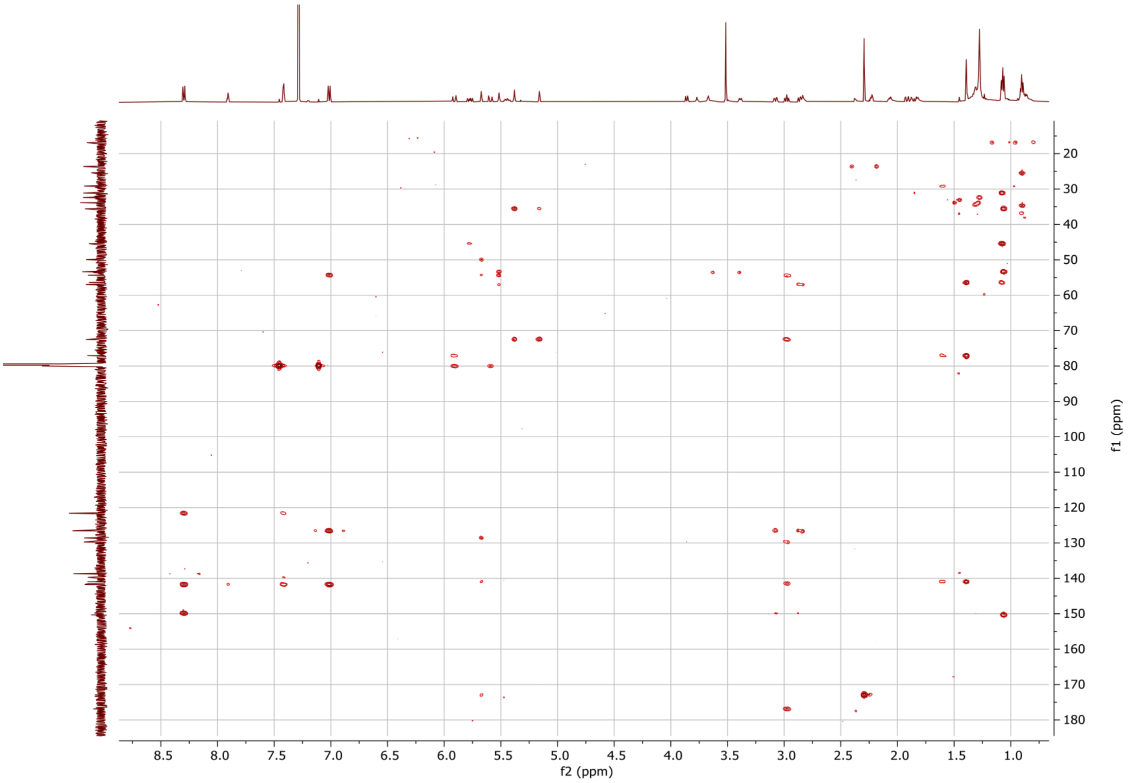

Figure S43. HMBC NMR of azulenochalasin H **15a.**

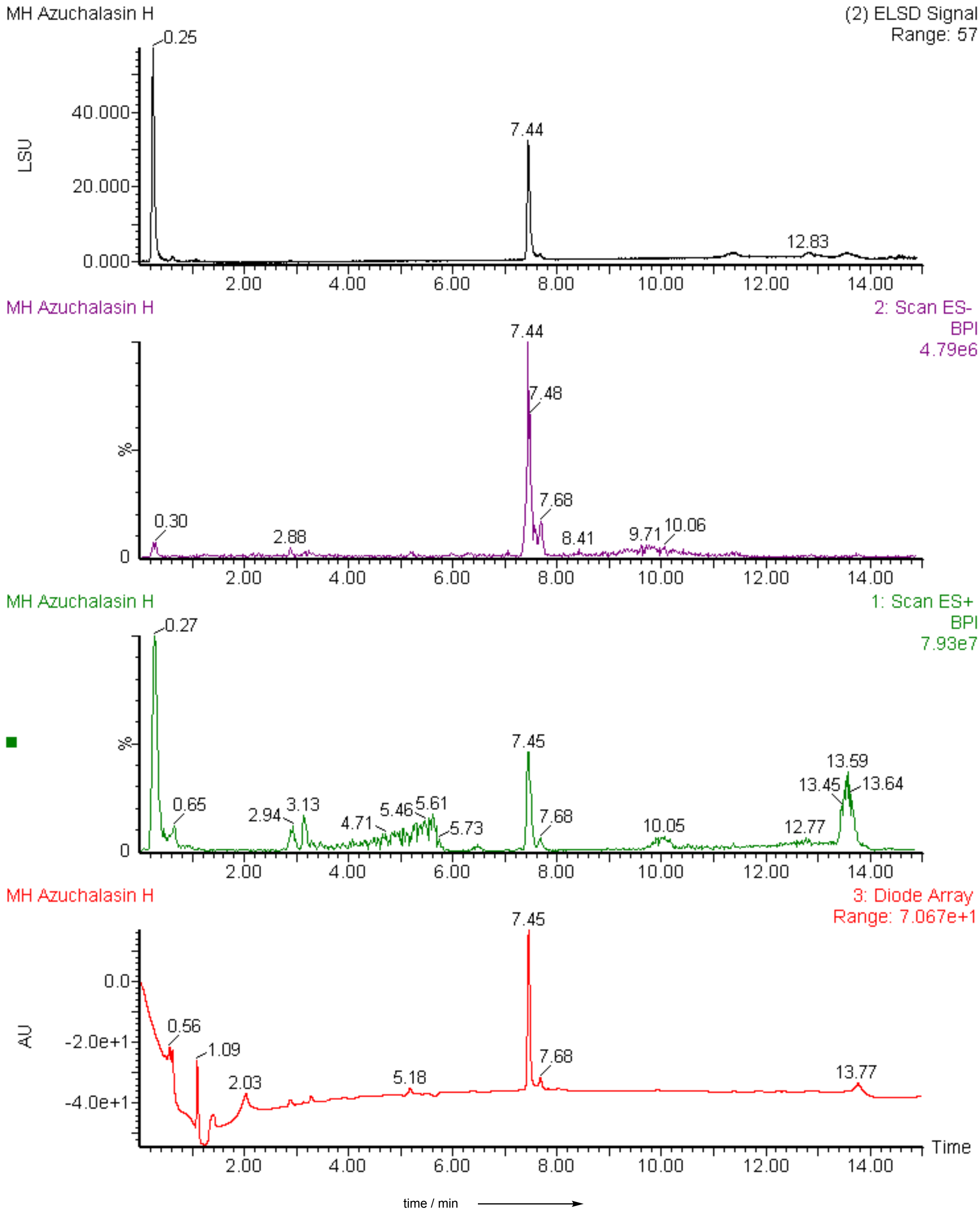

Figure S44. LCMS analysis of purified **15a.** From top: evaporative light scattering detector; Total ion current, ES-, (100 - 1000 *m/z*); Total ion current, ES+, (100 - 1000 *m/z*); Diode array detector (200 - 600 nm).

15b (6-(1-bromo)azuleno)chalasin H.

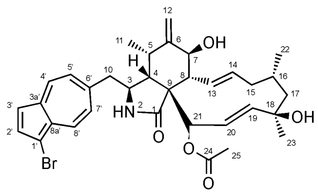

Chemical Formula: $C_{34}H_{40}BrNO_5$
Exact Mass: 621.2090

UV$_{\lambda max}$ (MeOH): 222, 285, 295, 349 nm; **HRMS (ESI):** calc. for $C_{34}H_{39}{}^{79}BrNO_5$ $[M-H]^-$: 620.2012; found: 620.1989

Position	δ$_H$	M	J_{H-H} / Hz	δ$_C$ HSQC/HMBC	HSQC	HMBC H to C	H-H COSY
**1**	-	-	-	176.8	-	-	-
**2**	5.49	brs	-	-	-	-	-
**3**	3.38	m	-	56.8	CH	-	4, 10a, 10b
**4**	2.23	dd	3.8, 7.4	53.2	CH	-	3, 5
**5**	2.83	m	-	35.5	CH		4, 11
**6**	-	-	-	150.3	-	-	-
**7**	3.86	d	10.7	72.3	CH	-	8
**8**	2.97	dd	10.7, 9.6	49.9	CH	1, 7, 9, 13, 14	7, 13
**9**		-	-	51.9	-	-	-
**10a**	2.89	dd	13.0, 9.6	54.4	CH_2	3, 6'	3, 10b
**10b**	3.07	dd	13.0, 4.7			3, 6'	3, 10a
**11**	1.04	d	6.7	16.2	CH_3	4, 5, 6	5
**12a**	5.16	brs	-	117.3	CH_2	5,7	5, 7, 12b
**12b**	5.38	brs	-			5,7	5, 7, 12a
**13**	5.78	dd	15.5, 9.6	129.8	CH		8, 14
**14**	5.45	ddd	15.4, 10.3, 5.0	141.8	CH	8(w)	13, 15a, 15b
**15a**	1.84	m	-	45.4	CH_2	16(w)	14,16, 15b
**15b**	2.07	m	-			16(w)	14, 15a
**16**	1.82	m	-	31.2	CH		-
**17a**	1.62	m	-	56.4	CH_2	16, 23	17b
**17b**	1.92	dd	3.4, 14.5			-	16, 17a
**18**	-	-	-	77.1	-	-	-
**19**	5.60	dd	16.5, 2.3	141.1	CH	21	20, 21
**20**	5.90	dd	16.6, 2.3	128.6	CH	21, 18	19, 21
**21**	5.68	d	2.5	80.0	CH	8, 19, 20, 24	19, 20
**22**	1.08	d	6.5	29.2	CH_3	15, 16, 17	16
**23**	1.39	s	-	33.9	CH_3	17, 18, 19	-
**24**	-	-	-	173.1	-	-	-
**25**	2.30	s	-	23.4	CH_3	24	-
**6'**	-	-	-	151.6	-	-	-
**7'**	7.09	d	10.1	127.1	CH	10, 5', 8a'	8'
**8'**	8.30	d	10.1	138.1	CH	1', 3a', 6'	7'
**8a'**	-	-	-	136.8	-	-	-
**1'**	-	-	-	107.8	-	-	-
**2'**	7.82	d	4.1	140.4	CH	8a', 3a'	3'
**3'**	7.35	d	4.1	120.4	CH	1', 8a', 4'	2'
**3a'**	-	-	-	141.6	-	-	-
**4'**	8.20	d	9.7	139.3	CH	3', 8a', 6'	5'
**5'**	7.04	d	9.7	127.4	CH	10, 7', 3a'	4'

Table S2: Summarized NMR signals and 2D-correlations for **15b** recorded in $CDCl_3$ at 600 MHz / 150 MHz.

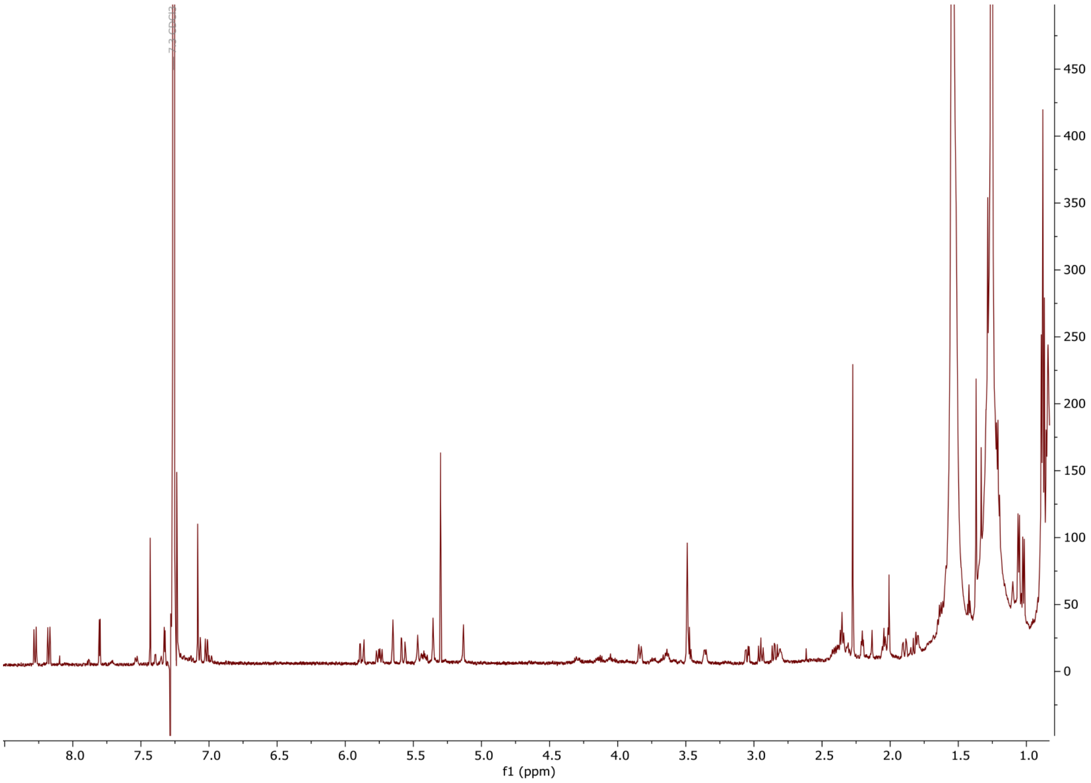

Figure S45. ^{1}H NMR spectrum of (6-(1-bromo)azuleno)chalasin H **15b**.

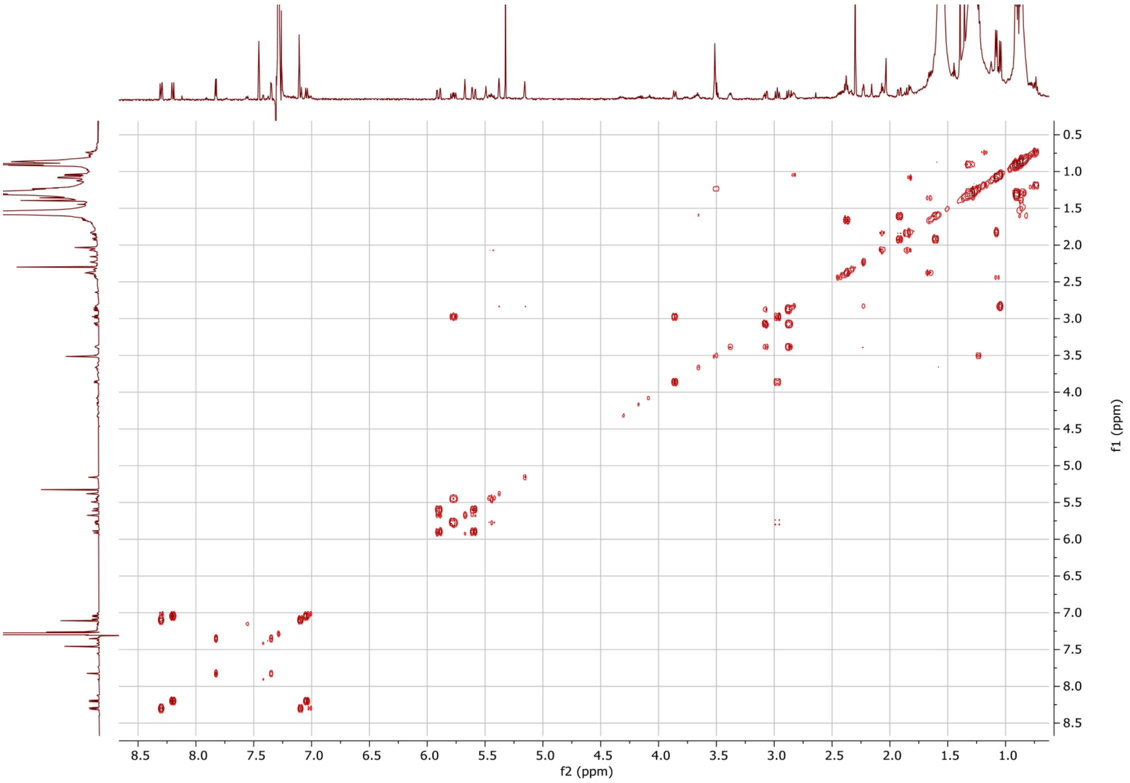

Figure S46. COSY NMR spectrum of (6-(1-bromo)azuleno)chalasin H **15b**.

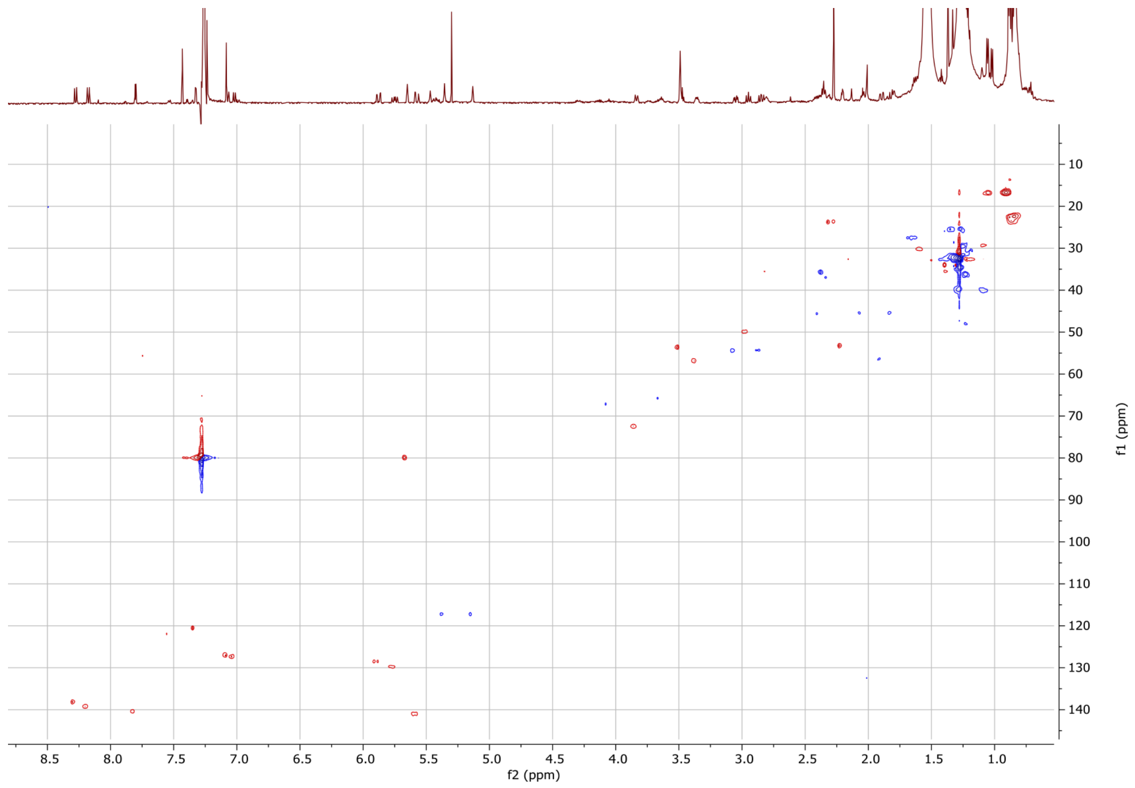

Figure S47. HSQC spectrum of (6-(1-bromo)azuleno)chalasin H **15b**.

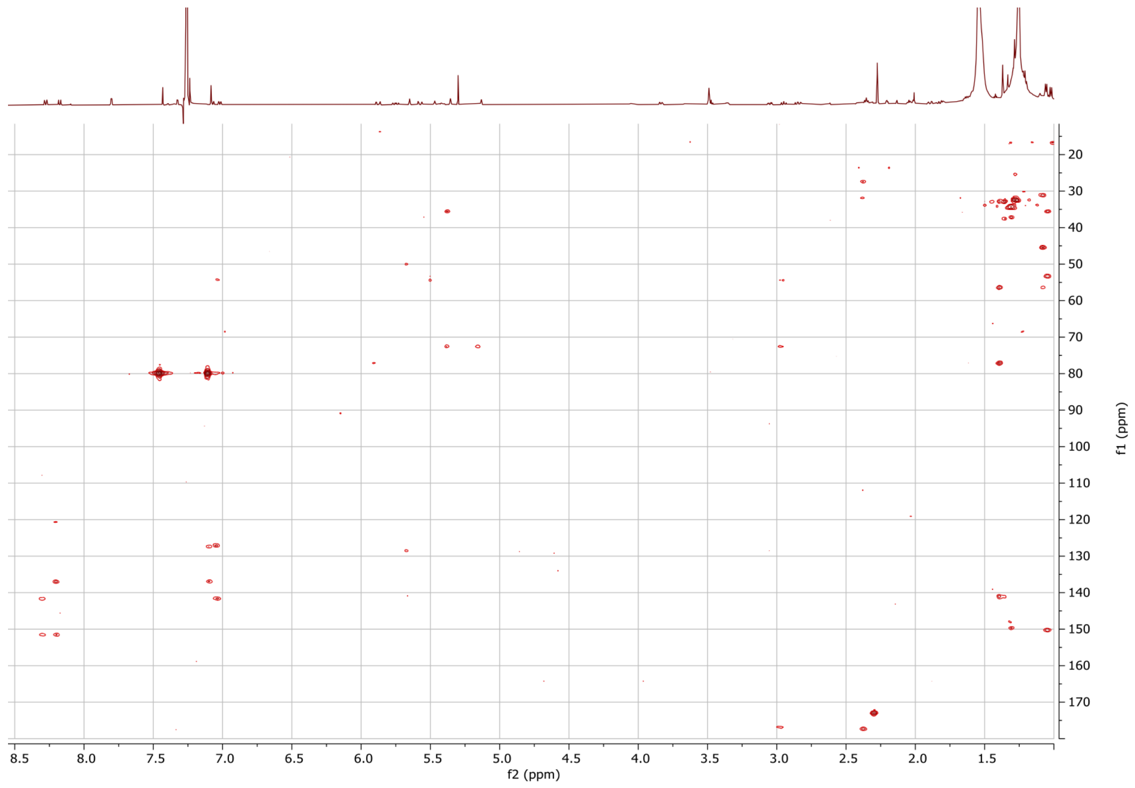

Figure S48. HMBC spectrum of (6-(1-bromo)azuleno)chalasin H **15b**.

Fluorescence Measurements for 14a and 15b

Compound **15a** and **15b** were diluted in MeOH at a concentration of 1 mg/mL and added onto a 96-well black/clear bottom plate. The plate was placed into a microplate reader (BioTek - Agilent Technologies, Santa Clara, USA) and the absorption spectra in a range of 300 nm to 700 nm in 10 nm steps were measured. Based on estimated absorption maxima of **15a** (340 nm) and **15b** (350 nm), the emission spectra were determined in a range of 300/20 nm to 700 nm in 10 nm steps using an excitation of 340/10 nm.

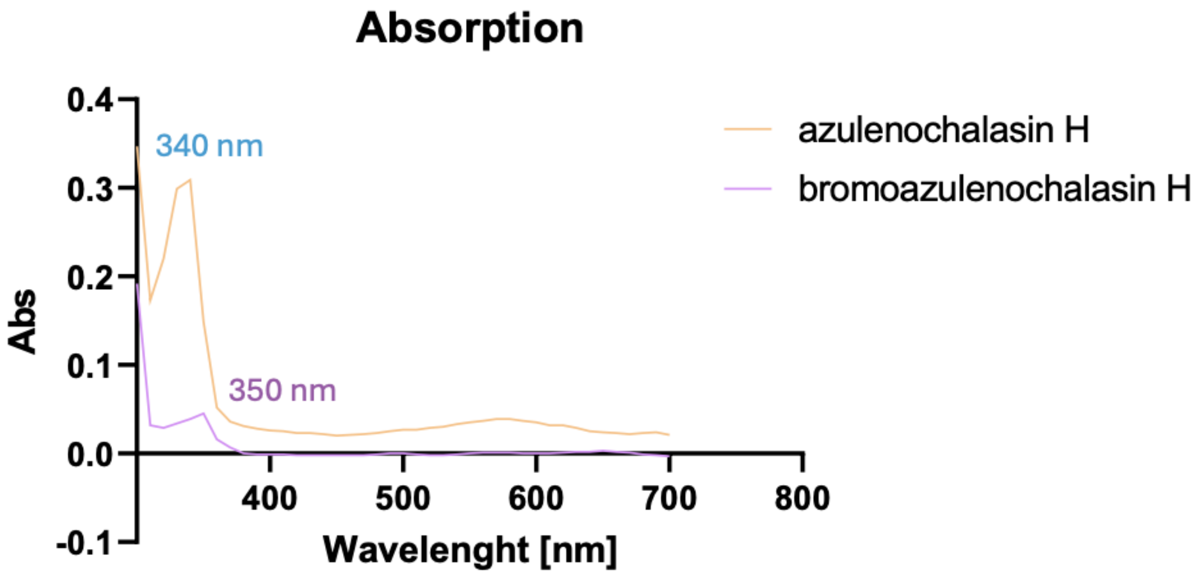

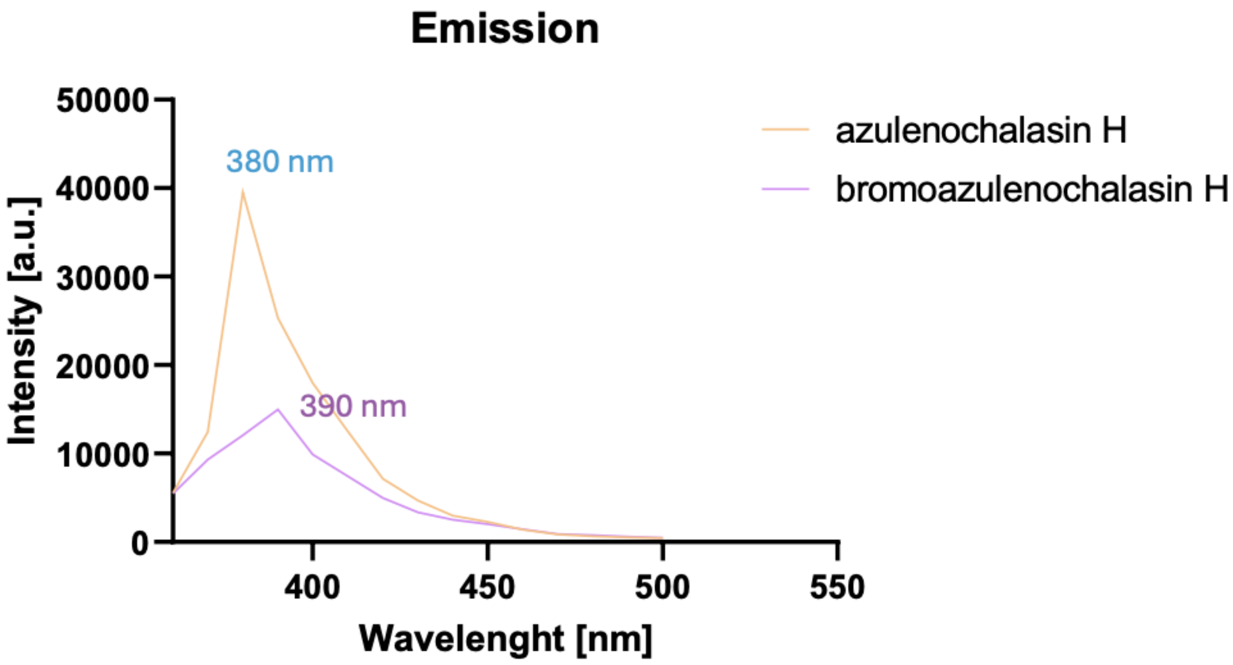

3. Biological Testing

Cell Culture

The mammalian cell line L929 (mouse fibroblast, DSMZ ACC 2) was cultured in Dulbecco's modified minimum essential medium (DMEM, Life Technologies, Carlsbad, USA) containing 10% fetal bovine serum (FBS, Life Technologies). The human osteosarcoma cell line (U-2OS, ATCC HTB-96) was maintained in DMEM (Life Technologies, Carlsbad, USA) supplemented with 10% FBS (Sigma- Aldrich, St. Louis, MO, USA), 1% L-glutamine (Life Technologies, Carlsbad, USA), 1% sodium-pyruvate (Life Technologies, Carlsbad, USA), 1% minimum essential medium nonessential amino acids (MEM NEAA, Life Technologies, Carlsbad, USA), and 1% Penicillin-Streptomycin (10.000 U/ml, Life Technologies, Carlsbad, USA). Both cell lines were routinely cultured at 37 °C and 7.5% CO_2.

Cytotoxicity Assay

The cytotoxicity of **15a** and **15b** was determined in mouse fibroblast L929 cells by a colorimetric tetrazolium dye MTT assay[9] described in detail by Becker and coworkers.[10] Briefly, L969 cells were seeded onto a 96-well plate at 50,000 cells/mL density and allowed to attach for 30 min. A serial dilution of **15a** and **15b** was prepared and added to the cells for an incubation period of five days. Cells were stained using the MTT dye and the half-maximum inhibitory concentration (IC_{50}) was determined based on the colour change of the dye from yellow to purple measured at 595 nm using a microplate reader (Tecan, Männedorf, Switzerland).

*Table S3: Determined cytotoxicity values (IC50) of **15a** and **15b** compared to positive control epothilone B in mouse fibroblast cell line L929 and cervix carcinoma cell line KB3.1.*

Compound	L929 [µM]	KB3.1 [µM]
**15a**	0.33 (0.179 µg/mL)	0.053 (0.0288 µg/mL)
**15b**	0.29 (0.181 µg/mL)	n.d.
**Pyrichalasin H**[5]	0.21 (0.109 µg/mL)	0.019 (0.010 µg/mL)
**Epothilone B** (positive control)	0.00095 (0.48 ng/mL)	0.000051 (0.025 ng/mL)

n.d. (not determined)

Actin Disruption Assay

To investigate the impact of **15a** and **15b** on the actin cytoskeleton, a well-established actin disruption assay, based on the protocol reported by Kretz et al.[11] was performed. In brief, human osteosarcoma cells (U-2OS) were seeded at a density of 20,000 cells on fibronectin-coated (25 µg/mL diluted in phosphate buffered saline – PBS, Life Technologies, Carlsbad, USA) coverslips and allowed to spread over night under cell culture conditions. Pre-warmed cell culture medium was supplemented with **15a** and **15b** at concentrations corresponding to the previously determined IC_{50} values ($1 \times IC_{50}$ = low dose; $5 \times IC_{50}$ = high dose). Cells were treated for 1 h under cell culture conditions. Subsequently, cells were fixed with 4% pre-warmed para-formaldehyde (PFA) diluted in PBS for 20 min. To test for reversibility of the affected actin network upon high dose treatment, cells were washed thrice in pre-warmed PBS and cultured in compound-free medium for 1 h prior to fixation. Fixed cells were washed thrice in pre-warmed PBS and permeabilized using 0.1% Triton X-100 (Bio-Rad Laboratories, Hercules, USA) diluted in PBS for 1 min. After additional washing steps with PBS, cells were stained for filamentous actin (F-actin) using AttoFluor-488-coupled phalloidin for 1 h at room temperature. Coverslips were washed thrice in PBS before mounting in ProLong Diamond Antifade Mountant (Invitrogen, Carlsbad, USA) containing DAPI for nuclear DNA staining. Cell images were acquired using an inverted microscope (Nikon eclipse Ti2, Tokio, Japan) equipped with a 60 × Nikon oil immersion objective (Plan Apofluar, 1.4 NA), a pco.edge back-illuminated sCMOS camera (Excelitas Technologies, Mississauga, ON, Canada), and a pE-4000 (CoolLED, Andover, UK) as light source. The microscope system was operated by and images recorded with NIS-Elements software (Nikon, Tokio, Japan). The images were processed further using FiJi (Image J, NIH, Bethesda, MD, USA).

4. References

1. Leino, T. O.; Baumann, M.; Yli-Kauhaluoma, J.; Baxendale, I. R.; Wallén, E. A. A. *J. Org. Chem*., 2015, **80**, 11513–11520.
2. S. E. Estdale, R. Brettle, D. A. Dunmur and C. M. Marson, *J. Mater. Chem.*, 1997, **7**, 391–401.
3. T. Takayasu and M. Nitta, *J. Chem. Soc., Perkin Trans. 1*, 1997, **0**, 3537–3542.
4. Marfey, P., *Carlsberg Res. Commun.* 1984, **49**, 591.
5. C. Wang, C. Lambert, M. Hauser, A. Deuschmann, C. Zeilinger, K. Rottner, T. E. B. Stradal, M. Stadler, E. J. Skellam and R. J. Cox, *Chem. Eur. J.*, 2020, **26**, 13578–13583.
6. C. Wang, V. Hantke, R. J. Cox and E. Skellam, *Org. Lett.*, 2019, **21**, 4163–4167.
7. H. M. Ge, W. Yan, Z. K. Guo, Q. Luo, R. Feng, L. Y. Zang, Y. Shen, R. H. Jiao, Q. Xu and R. X. Tan, *Chem. Commun.*, 2010, **47**, 2321–2323.
8. E. Stempel, R. F.-X. Kaml, N. Budisa and M. Kalesse, *Bioorg. Med. Chem.*, 2018, **26**, 5259 - 5269. DOI:10.1016/j.bmc.2018.03.037.
9. T. Mosmann, *J. Immunol. Methods*, 1983, **65**, 55–63..
10. K. Becker, A.-C. Wessel, J. J. Luangsa-ard and M. Stadler, *Biomolecules*, 2020, **10**, 805.
11. R. Kretz, L. Wendt, S. Wongkanoun, J. J. Luangsa-ard, F. Surup, S. E. Helaly, S. R. Noumeur, M. Stadler and T. E. B. Stradal, *Biomolecules*, 2019, **9**, 73.
